# If you rise, I fall: Equality is prevented by the misperception that it harms advantaged groups

**DOI:** 10.1126/sciadv.abm2385

**Published:** 2022-05-06

**Authors:** N. Derek Brown, Drew S. Jacoby-Senghor, Isaac Raymundo

**Affiliations:** 1 University of California, Berkeley, Berkeley, CA, USA.; 2 Columbia University, New York, NY, USA.

## Abstract

Nine preregistered studies (*n* = 4197) demonstrate that advantaged group members misperceive equality as necessarily harming their access to resources and inequality as necessarily benefitting them. Only when equality is increased within their ingroup, instead of between groups, do advantaged group members accurately perceive it as unharmful. Misperceptions persist when equality-enhancing policies offer broad benefits to society or when resources, and resource access, are unlimited. A longitudinal survey of the 2020 U.S. voters reveals that harm perceptions predict voting against actual equality-enhancing policies, more so than voters’ political and egalitarian beliefs. Finally two novel-groups experiments experiments reveal that advantaged participants’ harm misperceptions predict voting for inequality-enhancing policies that financially hurt them and against equality-enhancing policies that financially benefit them. Misperceptions persist even after an intervention to improve decision-making. This misperception that equality is necessarily zero-sum may explain why inequality prevails even as it incurs societal costs that harm everyone.

## Teaser

Advantaged groups misperceive equality as harming their resource access, which predicts voting against equality policies.

## INTRODUCTION

Members of societally advantaged groups frequently support the concept of equality (*1*, *2*) and yet use their advantaged position to implement policies that perpetuate inequality (*3*). This tendency persists even as inequality threatens the prosperity of disadvantaged and advantaged groups alike. Racial inequality costs the U.S. economy an estimated $16 trillion in lost gross domestic product (GDP) (*4*), failing to hire job seekers with a criminal history results in an annual loss of $78 billion in U.S. GDP (*5*), and the persistent gender pay gap restrains the global economy by about $160 trillion (*6*). These overall economic costs harm advantaged group members as well (*7*). Why then do the members of society who hold the greatest power to create change instead allow inequalities to continue, or worsen, even as they exact a toll on everyone? We argue that it is because advantaged group members misperceive that equality necessarily comes at a cost to their group.

Decades of research make it clear that people closely attend to how well-off they are compared to others and behave in ways that maximize their relative advantages (*8*, *9*). As a result, people often perceive situations to be zero-sum, even in situations that are not (*10*–*12*). For instance, the “fixed pie bias” leads negotiators to see their interests as unavoidably opposed to those of their counterpart, even when there exist opportunities to improve the well-being of one or both parties without harming either (*13*–*15*). People even construe everyday transactions—such as buying food or purchasing a car—as resulting in a winner and a loser (*16*). These beliefs can cause policy-makers and voters to perceive that new policies will negatively affect them more than they will benefit others, even when the opposite is true (*17*). The result is that societies often continue to suffer under suboptimal public policies (*18*).

We posit that this same focus on relative advantages leads people to misperceive the effects of equality. Researchers have long known that people prefer an unequal distribution of resources, such that greater amounts of resources are allocated to ingroup members than to outgroup members (*19*–*21*). This preference often persists even when a more equal distribution of resources would benefit one’s ingroup. For instance, people will choose to receive fewer resources if that choice secures their group’s relative advantage over an outgroup (i.e., Vladimir’s choice) (*22*). Building upon this research, we propose a new explanation for this phenomenon—that people fundamentally misperceive losses of relative advantage as losses in absolute terms. We hypothesize that advantaged group members perceive policies that reduce disparities between groups as reducing resource access for the advantaged group, even when this is not the case. We conversely predict that advantaged group members perceive policies that worsen or maintain disparities between groups as increasing resource access for the advantaged group, even when this is not so. As a result, advantaged groups are likely to oppose non–zero-sum increases in resource equality on the basis of mistaken assumptions that they are protecting their access to these resources.

In contrast to our hypothesis, past work often involves two approaches that make it difficult to determine whether perceptions of harm are at all inaccurate. First, past work has often focused on the argument that people who are ideologically opposed to equality perceive that it symbolically harms their group. Prejudice toward disadvantaged outgroups (*23*), political conservatism (*24*), support for the idea that society is zero-sum (*25*), ideological support for the status quo (*26*), and preference for social hierarchies between groups (*27*, *28*) have all been associated with feeling threatened by equality policies. Although we, too, expect that antiegalitarian ideologies increase perceptions that equality-enhancing policies harm advantaged groups, we predict that misperceptions also occur independently of these ideologies.

A second common approach in prior work is a focus on dynamics that are plausibly zero-sum. In some such studies, materially better outcomes for disadvantaged groups do, in fact, make advantaged groups materially worse off (*29*). For example, researchers have studied whether participants are willing to hire fewer members of an advantaged group to hire more disadvantaged group members [e.g., (*30*, *31*)]. In other studies, scholars have examined zero-sum perceptions on symbolic dimensions, which are inherently difficult to disprove. For example, research has shown that majority group members feel that their status is threatened by the increasing size of minority groups (*32*) or shown that white Americans and men generally perceive reduced prejudice toward Black Americans and women as corresponding with increased bias toward their own groups (*33*, *34*). This extant work does not consider the possibility that policies can objectively increase equality between two groups in a materially non–zero-sum manner.

We address this issue in the present paper by asking participants to evaluate policies that are definitionally non–zero-sum, wherein one group’s gain is not symmetric to another groups’ loss (*35*). Specifically, we assess their perceptions of policies that change disadvantaged groups’ access to a material resource without changing advantaged groups’ access to that same resource (e.g., increasing the number of jobs available to disabled people without changing the number of jobs available to nondisabled people). We therefore define misperceptions as viewing equality-enhancing policies as harmful—or, conversely, inequality-perpetuating policies as beneficial—to advantaged group members’ access to that particular resource (e.g., jobs) when no such change has occurred.

Nine preregistered laboratory and field studies involving numerous social contexts tested the hypothesis that members of societally advantaged groups perceive increased equality as harmful to their resource access even when it is not. In studies 1 to 5, participants read various equality-enhancing policies that would provide additional resources (e.g., salary and jobs) to a disadvantaged group (e.g., Latino Americans, people with a disability, women, and ex-felons) without changing the resources provided to the advantaged ingroup (e.g., white Americans, people without a disability, men, and non-felons). We explored whether advantaged group members misperceived these equality-enhancing policies as harmful to their group’s resource access: when compared against inequality-enhancing and status quo–preserving policies that affected them identically (studies 1a and 1b), when proposing to increase equality between groups but not when increasing equality within their ingroup (study 2), when explicitly framed as benefitting society as a whole (study 3), and when resources (study 4) or access to resources (study 5) are explicitly limited or unlimited. Studies 1 to 5 used the same basic materials and procedures. Pertinent changes are detailed for each experiment.

We additionally tested whether the misperception that equality harms their resource access explains advantaged group members’ opposition to implementing equality-enhancing policies. Study 6 tested the ecological validity of our hypothesis, specifically whether registered voters’ perceptions that an equality-enhancing policy on the November 2020 California general election ballot was harmful to their resource access predicted their likelihood of voting against the policy, above and beyond their political and egalitarian ideologies. We then used a novel-groups paradigm in studies 7 and 8 to definitively test whether advantaged group members’ misperceptions persist even when the groups are arbitrarily defined, the context is undeniably non–zero-sum, and participants’ policy decisions directly financially affect them. In study 7, we examined whether participants misperceived a win-win equality-enhancing policy as harmful to the advantaged ingroup’s chances of receiving money compared to a lose-lose inequality-enhancing policy and whether these misperceptions predicted voting against greater equality. Finally in study 8, we explored whether established means of improving deliberative decision-making [i.e., joint evaluation (*36*)] would help advantaged group members perceive an equality-enhancing policy more favorably.

See Table 1 for study details and hypothesized outcome for all studies. Across all studies, we measured five prominent forms of ideological opposition to equality to rule them out as explanatory mechanisms (see tables S2 to S28). Data, code, and preregistrations for all studies have been made available on Open Science Framework (OSF; https://osf.io/ksf4q/).

**Table 1.** List of hypothesized effects and variables across experiments. In the hypothesized effects column, arrows indicate the predicted relationship to scale midpoint; different symbols indicate predicted differences between conditions. NGP, novel group paradigm; SDO, social dominance orientation; ZSB, global zero-sum beliefs. DV, dependent variable. ✔ denotes significant outcome (*P* < 0.05). ✘ denotes nonsignificant outcome.

			**Outcome significance**			**Ideological control variables**					
**Study**	**Hypothesized** **Effects**	**Additional** **Study features**	**Perceived** **Resource** **Access**	**Policy** **Support**	**Vote**	**SDO**	**Poli** **Orient**	**ZSB**	**Explicit** **prej.**	**Status** **Threat**	**Symbolic** **Threat**
**1a**	Equality ↓ Inequality↑ Status quo ↑		✔			✔	✔	✔	✔		
**1b**	Equality ↓ Inequality ↑	All representational disparities; conservatively worded DV	✔			✔	✔	✔	✔	✔	✔
**2**	Intergroup equality ↓ Ingroupequality ↑	Policies specify average resources; advantage explicitly unearned	✔			✔	✔	✔	✔		
**3**	Equality benefits society ↓ Inequalityharms society ↑		✔			✔	✔	✔	✔		
**4**	Limited or unlimited resources: Equality ↓ Inequality ↑		✔			✔	✔	✔	✔		
**5**	Limited resource access equality ↓ ↓Unlimited resource access equality ↓	Subset analyses with strict attention check	✔			✔	✔	✔	✔		
**6**	Real-world equality policy ↓	Longitudinal field survey of registered voters (correlational)	✔	✔	✔	✔	✔	✔	✔		
**7**	Win-win equality ↓ Lose-lose inequality ↑	NGP; voting determined compensation	✔	✘	✘	✔	✔	✔	✔		
**8**	Unharmful equality ↓ Unharmful equality presented jointly with harmfulequality ↑	NGP; voting determined compensation; advantage explicitly unearned	✘	✔	✔	✔	✔	✔	✔		

## RESULTS

### Study 1a

We tested whether advantaged group members perceive equality-enhancing policies as more harmful to their resource access compared to inequality-enhancing policies (studies 1a and 1b) or policies that conserve the status quo (study 1a), even when policies have the same impact on their group. In study 1a (https://osf.io/9y876/), we presented 594 white (non-Hispanic) participants with descriptions of three different real-world inequalities, randomly selected from a set of six inequalities, between an advantaged ingroup and a disadvantaged outgroup. Each vignette described a different inequality context to ensure that our findings were not specific to any particular disparity or group. These included both monetary (e.g., salary) and representational inequalities (e.g., jobs) (see Materials and Methods).

For each inequality, participants first read a description of the disparity between an advantaged and disadvantaged group (e.g., “According to a recent report, in 2018, White homebuyers received roughly $386.4 billion in mortgage loans from banks, while Latino homebuyers only received around $12.6 billion in mortgage loans overall”). In the equality-enhancing policy condition, participants read proposals to increase resource access for the disadvantaged group and not change resource access for the advantaged group (e.g., “Several banks propose increasing the total amount of mortgage loans to Latino homebuyers by $7.3 billion and not changing the total amount of mortgage loan funding to White homebuyers”). The policy would thereby increase proportional equality in access to a given resource between the two groups. In the inequality-enhancing policy condition, participants read proposals to decrease resource access for the disadvantaged group and not change resource access for the advantaged group (e.g., “Several banks propose decreasing the total amount of mortgage loans to Latino homebuyers by $7.3 billion and not changing the total amount of mortgage loan funding to White homebuyers”). In the status quo policy condition, participants read proposals that no changes be made to address the disparity (e.g., “However, several banks propose not changing mortgage loan funding over the next year”). We then measured perceived changes to advantaged ingroup resource access (e.g., “How do you think this proposal will affect White homebuyers’ chances of getting mortgage funding from these banks next year”) using a 7-point Likert scale (−3 = greatly harm, 0 = no effect, +3 = greatly improve). Crucially, the resources available to the advantaged group were actually identical across conditions. We defined misperceptions as rating equality-enhancing policies as reducing resource access for the advantaged group and inequality-enhancing or status quo policies as increasing their resource access, even when this was not the case.

**Fig. 1.**
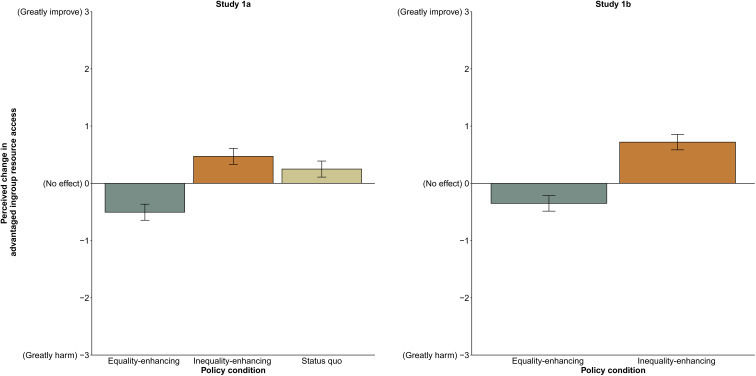
Perceptions of how policies affect the advantaged ingroup’s access to resources, studies 1a (*n* = 594) and 1b (*n* = 399). Means are adjusted on the basis of the participant and vignette random effects included in the linear mixed model. Error bars indicate 95% CIs around the mean.

As predicted, participants misperceived equality-enhancing policies as more harmful to their advantaged ingroup’s resource access than policies that maintained the status quo [*b* = 0.75, SE = 0.087, *t*(590.68) = 8.62, *P* < 0.001, 95% confidence interval (CI) [0.58, 0.93]] or worsened inequality [*b* = 0.98, SE = 0.087, *t*(590.22) = 11.16, *P* < 0.001, 95% CI [0.80, 1.15]]. Participants misperceived inequality-enhancing policies as more beneficial to their resource access relative to policies that preserved the status quo [*b* = −0.22, SE = 0.09, *t*(590.55) = −2.54, *P* = 0.011, 95% CI [−0.39, −0.05]]. As indicated by the 95% CIs around each condition mean in Fig. 1, participants misperceived equality-enhancing policies as significantly harmful to the advantaged ingroup’s resource access (*M* = −0.50, SD = 1.05), whereas participants misperceived inequality-enhancing (*M* = 0.47, SD = 1.23) and status quo–preserving policies (*M* = 0.25, SD = 0.96) as improving their access to resources.

For this and all other experiments, we measured five forms of ideological opposition to equality: political conservatism, social dominance orientation (*37*), belief that society is zero-sum (*38*), system-justifying beliefs (*39*), and explicit prejudice (*40*). Although these ideological beliefs occasionally correlate with perceived ingroup resource access, all results persist when controlling for ideological beliefs. We also found no strong or consistent evidence that any ideology moderates our effects. In short, the results are not merely explained by variation in ideological support or opposition. For succinctness, we present the ideology results for all causal experiments in tables S2 to S28.

### Study 1b

We ran study 1b to address three alternative explanations for these results. First, it is possible that participants interpreted our dependent variable as assessing how the advantaged group’s resource access changed relative to the disadvantaged group’s access. We adjusted the wording of this question to clarify its intended meaning—how the advantaged group’s resource access changed relative to the advantaged group’s access in the past (e.g., “How do you think this proposal will affect White homebuyers’ chances of buying a home next year compared to their chances of buying a home from these banks in the past?”). Second, participants could have perceived policies that altered financial resources (e.g., mortgage loans) as also altering the value of those financial resources and, thus, as altering purchasing power (i.e., causing inflation or deflation). Study 1b therefore exclusively used policies that focused on representational disparities (e.g., homeownership). Finally it is possible that our effect is present only among participants who feel that equality leads to symbolic losses for the advantaged group. We therefore measured both symbolic and group status threats in this study.

We randomly assigned 399 white (non-Hispanic) participants to read either an equality-enhancing policy condition or an inequality-enhancing policy condition (https://osf.io/9q35s/). All unspecified details were identical to study 1a. As predicted, participants misperceived equality-enhancing policies (*M* = −0.35, SD = 1.07) as more harmful to their advantaged ingroup’s resource access than inequality-enhancing policies [*M* = 0.72, SD = 1.75, *b* = 1.07, SE = 0.098, *t*(397) = 10.97, *P* < 0.001, 95% CI [0.88, 1.26]] (see Fig. 1). This effect remained significant when controlling for ideological beliefs, including symbolic and group status threats. Furthermore, symbolic and status threats did not moderate results, indicating that the effect is not driven by the feeling that equality incurs symbolic costs (see tables S4 and S5). Studies 1a and 1b indicate that advantaged group members misperceive reductions, preservation, and increases in their relative advantage (i.e., inequality) as changing their resource access in an absolute sense.

### Study 2

Are there contexts in which participants perceive non–zero-sum reductions of inequality more accurately? Social identity theory suggests that people are motivated to both minimize ingroup differences and maximize intergroup differences (*41*). Perhaps participants would perceive equality as less harmful when it occurs within their ingroup (e.g., reducing a disparity between white Americans) compared to when it occurs between an ingroup and an outgroup (e.g., reducing a disparity between white Americans and Black Americans). Such a result would help explain why racially homogeneous countries enact more equality-enhancing policies than racially heterogeneous countries (*42*, *43*).

Study 2 additionally accounted for two alternative explanations for our results. First, we specified that there was no justification for the disparity. This accounted for the possibility that participants spontaneously infer that certain people have earned their position in society and, thus, believe that equality would be detrimental to those who seemingly deserve their societally advantaged position. Second, we informed participants of each groups’ average resource access instead of the absolute amount available to each group. This accounted for the possibility that participants might infer that more equitable resource access would (for some unknown reason) increase the number of advantaged group members seeking out the resource, thereby reducing the average amount of resources available for their advantaged ingroup.

We randomly assigned 387 white (non-Hispanic) male participants to one of two conditions (https://osf.io/efz6j/). Participants in the ingroup equality-enhancing policy condition read about a disparity between equally deserving ingroup members (e.g., “According to a 2019 report, while most White homebuyers in a neighborhood received an average of $273,000 in mortgage loans from banks, comparable White homebuyers in the same neighborhood received an average of $249,000 in mortgage loans. There was no available explanation for this gap”). Participants in the intergroup equality-enhancing policy condition read about the same disparity, but between advantaged ingroup members (e.g., white homebuyers) and equally deserving yet disadvantaged outgroup members (e.g., Latino homebuyers). Participants then read a policy that would not change the average amount of resources provided to advantaged people but would increase the average amount of resources provided to disadvantaged people [e.g., “Several banks propose increasing mortgage loans by an average of $24,000 to the group of White homebuyers (Latino homebuyers) who tend to receive less and not changing the total amount of mortgage loan funding to the other White homebuyers”].

**Fig. 2.**
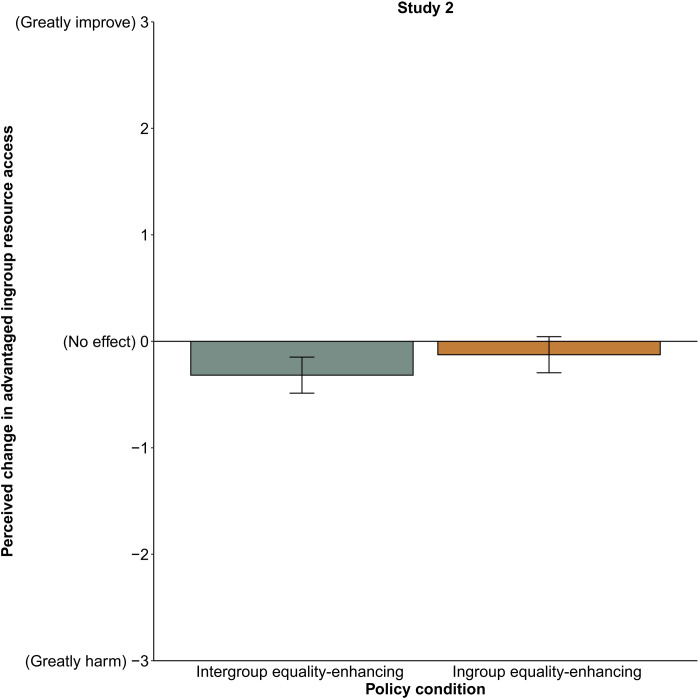
Perceptions of how policies affect the advantaged ingroup’s access to resources, study 2 (*n* = 387). Means are adjusted on the basis of the participant and vignette random effects included in the linear mixed model. Error bars indicate 95% CIs around the mean.

Participants misperceived intergroup equality-enhancing policies (*M* = −0.32, SD = 0.99) as more harmful to advantaged people’s resource access than ingroup equality-enhancing policies [*M* = −0.13, SD = 1.25), *b* = 0.19, SE = 0.096, *t*(385) = 2.00, *P* = 0.046, 95% CI [0.004, 0.38]]. As indicated by the 95% CIs around the mean in Fig. 2, participants more accurately perceived that disparity reductions within their ingroup would not harm advantaged people’s resource access. Thus, participants perceive policies that make everyone more equal as unharmful when the definition of “everyone” is restricted to people like them.

### Study 3

Although we designed our materials to specify that the advantaged group’s fate would be unchanged by the policy, in real life, society as a whole often benefits from increased equality and suffers when inequality worsens (*44*). Moreover, experts have argued that win-win policies, which are designed to simultaneously benefit everyone, are a way to increase advantaged group members’ support for equality-enhancing policies (*45*). Nonetheless, we tested whether advantaged group members view polices that increase equality and provide material societal benefits as more harmful than policies that decrease equality and incur societal costs.

Using stimulus templates from study 1a, we randomly assigned 393 white (non-Hispanic) participants to one of two conditions (https://osf.io/t4knh/). We told participants that either equality-enhancing policies would ultimately stimulate increased resources for everyone or inequality-enhancing policies would ultimately depress resources for everyone.

**Fig. 3.**
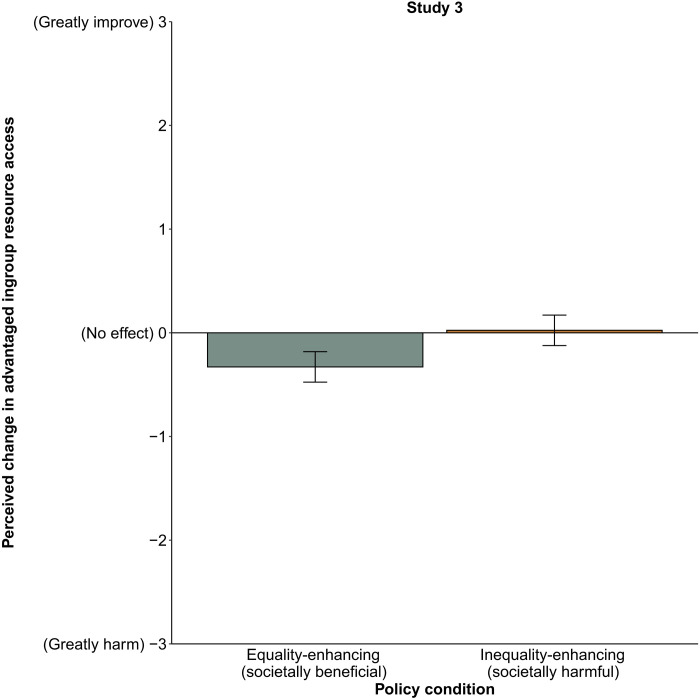
Perceptions of how policies affect the advantaged ingroup’s access to resources, study 3 (*n* = 393). Means are adjusted on the basis of the participant and vignette random effects included in the linear mixed model. Error bars indicate 95% CIs around the mean.

As predicted, participants misperceived societally beneficial equality-enhancing policies (*M* = −0.33, SD = 1.04) as more harmful to their advantaged ingroup’s resource access than societally harmful inequality-enhancing policies [*M* = 0.02, SD = 1.32; *b* = 0.35, SE = 0.098, *t*(390.61) = 3.61, *P* < 0.001, 95% CI [0.16, 0.54]]. This result (see Fig. 3) could explain why real-life inequalities endure despite the immense societal costs they produce.

### Study 4

We tested whether the misperception that equality-enhancing policies harm advantaged groups’ resource access depends on seeing resources as limited. Limited resources would mean policies have an opportunity cost of reduced future resource access for the advantaged group. Perhaps if advantaged group members knew that resources were boundless, then they might view equality-enhancing policies as non–zero-sum and, thus, as unharmful to them. Similarly, if resources would not need to be taken from one group to give to another, then advantaged group members might no longer view inequality-enhancing policies as benefitting them. However, we posit that advantaged group members primarily attend to whether their relative advantages over the outgroup increase or decrease. We therefore predicted that participants would view any equality-enhancing policy as more harmful to their resource access than inequality-enhancing policies regardless of whether resources were limited or unlimited.

We randomly assigned 393 white (non-Hispanic) participants to one of four conditions in an experiment using a 2 (equality-enhancing policy vs. inequality-enhancing policy) x 2 (limited resources vs. unlimited resources) between-subjects design (https://osf.io/x5etb/). In the limited resources condition, participants were explicitly told the resources for the proposal were finite. In the unlimited resources condition, participants were told that far more than enough resources were available to realize the policy.

**Fig. 4.**
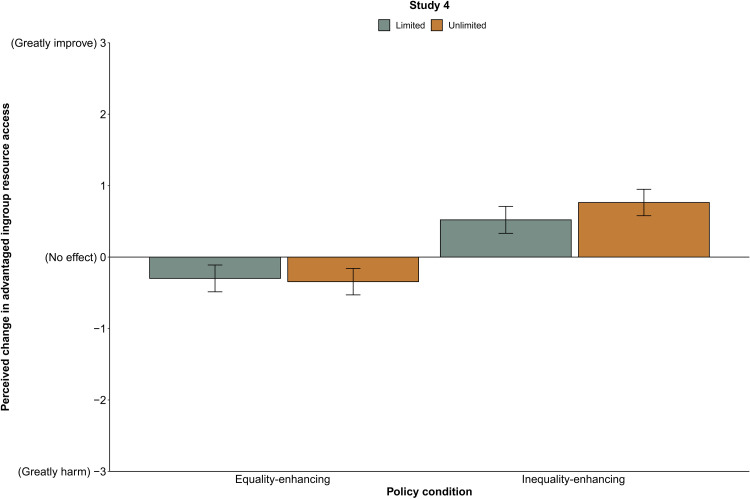
Perceptions of how policies affect the advantaged ingroup’s access to resources, study 4 (*n* = 393). Means are adjusted on the basis of the participant and vignette random effects included in the linear mixed model. Error bars indicate 95% CIs around the mean.

As predicted, there was a significant main effect of policy condition, *b* = 0.82, SE = 0.13, *t*(389.08) = 6.27, *P* < 0.001, 95% CI [0.56, 1.08], such that participants misperceived equality-enhancing policies (*M* = −0.32, SD = 1.05) as more harmful to the advantaged ingroup’s resource access than inequality-enhancing policies (*M* = 0.64, SD = 1.22). There was no significant main effect of resource condition, *b* = −0.05, SE = 0.13, *t*(388.93) = −0.35, *P* = 0.73, 95% CI [−0.29, 0.21], and no significant interaction between policy and resource conditions, *b* = 0.29, SE = 0.18, *t*(388.91) = 1.57, *P* = 0.12, 95% CI [−0.07, 0.65]. Participants’ understanding that there are more than enough resources to go around does nothing to diminish their misperception that equality causes harm to their group or that inequality benefits them (Fig. 4).

### Study 5

Just how persistent are these misperceptions? Would advantaged group members misperceive equality-enhancing policies as harming their access to resources even when directly told that the policies would not? We additionally preregistered using a strict attention check asking participants to recite the effect policies would have on each group involved. We thereby accounted for the possibility that our effects could be due to participants not reading or misreading the policies.

We randomly assigned 399 white (non-Hispanic) participants to one of two conditions. Across both conditions, we presented participants an equality-enhancing policy that would increase the amount of resources provided to the disadvantaged group and not change the amount of resources to the advantaged group (https://osf.io/x5etb/). In the limited access condition, we explicitly told participants that the equality-enhancing policy would limit the advantaged group’s access to the resource (e.g., “These banks will provide a limited number of mortgage loans, and the proposal will cause some White applicants to not receive funding”). In the unlimited access condition, we told participants that the equality-enhancing policy would allow for anyone in the advantaged group to access the resource (e.g., “Anyone who wants a mortgage loan can receive one”). Participants also completed an attention check, entering the exact amount by which the policy would change resources to each group.

**Fig. 5.**
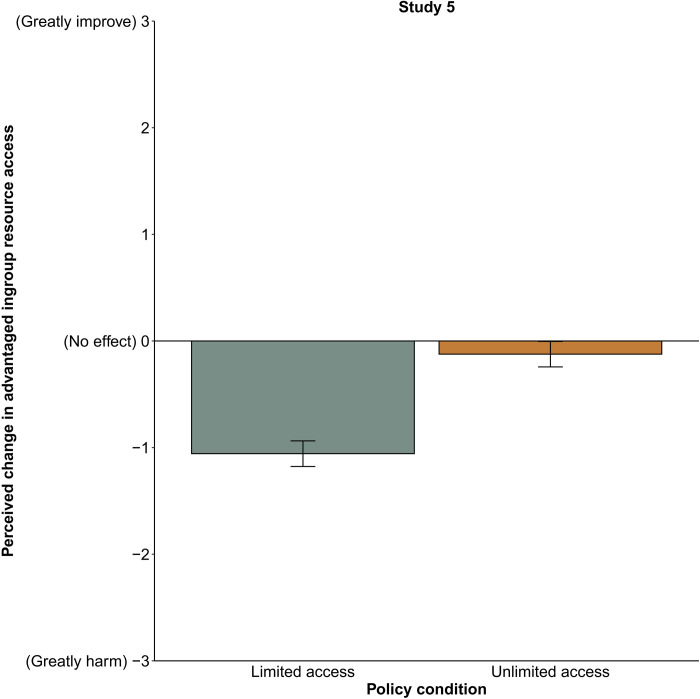
Perceptions of how policies affect the advantaged ingroup’s access to resources, study 5 (*n* = 399). Means are adjusted on the basis of the participant and vignette random effects included in the linear mixed model. Error bars indicate 95% CIs around the mean.

Participants perceived equality-enhancing policies that limited resource access (*M* = −1.06, SD = 1.12) as more harmful to the advantaged ingroup’s resource access than those that provided unlimited resource access [*b* = 0.93, SE = 0.08, *t*(396.01) = 11.12, *P* < 0.001, 95% CI [0.77, 1.10]]. However, participants misperceived equality-enhancing policies as harming the advantaged ingroup’s resource access even when access to resources was unlimited and, thus, should be perceived as improving access (*M* = −0.12, SD = 0.95, 95% CI [−0.24, −0.004]; see Fig. 5). Strikingly, even participants who correctly answered attention checks of key policy details misperceived equality-enhancing policies that provide unlimited access to resources as harming their resource access (*M* = −0.18, SE = 0.85, 95% CI [−0.30, −0.054]). These results suggest that participants’ misperception that equality harms advantaged ingroup resource access persists even when such concerns are directly addressed.

Across studies 1 to 5, we demonstrate that advantaged group members misperceive equality-enhancing policies as necessarily harming their resource access. Conversely, they misperceive the status quo– and inequality-enhancing policies as beneficial to their ability to access resources. These effects persist regardless of whether equality-enhancing policies produce broad societal benefits, whether available resources are limited or unlimited, and whether equality-enhancing policies explicitly do not limit the advantaged groups’ access to resources.

### Study 6

We next examined how pernicious perceptions of harm can be in preventing real-world progress toward a more equitable society within the context of the 2020 U.S. election. Specifically, we designed a two-part longitudinal study to investigate whether perceived changes in advantaged ingroup resource access predicted white and Asian Californians’ opposition to California Proposition 16, which proposed removing the ban on affirmative action in public employment and public university admissions decisions. The longitudinal design also allowed us to test whether increased perception over time that Proposition 16 harms ingroup resource access would predict reduced support and reduced voting in favor of the policy. In addition, we examined whether perceived harm to resource access predicts policy support above and beyond political orientation and antiegalitarian ideologies (https://osf.io/x5w87/).

We recruited white (non-Hispanic) and Asian registered California voters to participate in surveys at two different time points leading up to the November 2020 election. White and Asian Californians are advantaged in this context in that they constitute a greater proportion of public university students and public sector employees than they represent of the overall California population (*46*, *47*). We recruited 821 people (*n* = 411 white; *n* = 316 East Asian; *n* = 94 South Asian) to participate in the first survey (T1), 648 (*n* = 323 white; *n* = 251 East Asian; *n* = 74 South Asian) of whom also participated in the second survey (T2). Participants read the summary of Proposition 16 as it appeared on the ballot and then reported perceived advantaged ingroup resource access, policy support, and voting preference. Participants responded to the same ideological belief measures as in studies 1 to 5. Most participants identified as politically liberal (65.9%).

As predicted, white and Asian participants overall perceived Proposition 16 as harmful to their resource access [*M* = −0.26, SD = 1.36; one-sample *t*(820) = −5.56, *P* < 0.001, 95% CI [−0.36, −0.17]]. We also found a significant relationship between perceived advantaged ingroup resource access and policy support [*b* = 0.62, SE = 0.04, *t*(819) = 14.21, *P* < 0.001, 95% CI [0.53, 0.71]]. The more participants perceived Proposition 16 as harming their group’s chance of gaining placement in public employment, public education, and public contracting positions, the less they supported it. Similarly, perceived advantaged ingroup resource access significantly predicted voting preference. A one-point decrease in perceived ingroup resource access predicted 0.66 greater log odds of voting against Proposition 16 (odds ratio = 1.93, 95% CI [1.70, 2.21], SE = 0.07, *z* = 9.85, *P* < 0.001), meaning that a participant was 93.4% more likely to vote against Proposition 16. Importantly, perceived ingroup resource access significantly predicted policy support and voting preference controlling for— and more strongly than— numerous ideological variables (i.e., social dominance orientation, system-justifying beliefs, global zero-sum beliefs, explicit prejudice, and political orientation; see Table 2).

**Table 2.** The effect of perceived advantaged ingroup resource access on policy support remained significant, controlling for ideological beliefs, study 6 (*n* = 821). Ideological beliefs were explicit prejudice, SDO, system-justifying beliefs (SJB), ZSB, and political orientation. Similarly, the effect of perceived advantaged ingroup resource access on voting preferences remained significant, controlling for ideological beliefs. Vote preference was coded such that 0 = against and 1 = in favor. See tables S14 to S16 for results separated by participant race (white, East Asian, and South Asian).

	**Dependent variable**											
	**Policy support**						**Vote preference**					
Predictors	Estimates	SE	df	*t*	*P*	95% CI	Odds ratios	95% CI	Estimates	SE	*z*	*P*
(Intercept)	0.13	0.05	813	2.37	0.018	[0.02, 0.24]	1.51	[1.28, 1.78]	0.41	0.08	4.91	<0.001
Perceived ingroup resource access	0.50	0.04	813	11.91	<0.001	[0.41, 0.58]	1.86	[1.61, 2.14]	0.62	0.07	8.48	<0.001
Explicit prejudice	0.26	0.04	813	6.24	<0.001	[0.18, 0.35]	1.41	[1.24, 1.61]	0.34	0.07	5.13	<0.001
SDO	−0.23	0.06	813	−3.84	<0.001	[−0.35, −0.11]	0.83	[0.69, 1.00]	−0.19	0.10	−1.96	0.05
SJB	0.09	0.06	813	1.44	0.15	[−0.03, 0.20]	1.07	[0.89, 1.29]	0.070	0.10	0.74	0.46
ZSB	0.18	0.05	813	3.64	<0.001	[0.08, 0.27]	1.27	[1.09, 1.48]	0.24	0.08	3.10	0.002
Political orientation	0.21	0.04	813	4.89	<0.001	[0.13, 0.29]	1.28	[1.12, 1.45]	0.24	0.07	3.66	<0.001
Observations	820						820					
*R* ^2^	0.36											
*R*^2^ Tjur							0.271					

Changes in perceived ingroup resource access between T1 and T2 significantly predicted changes in both policy support and voting preference. Increased perceptions that Proposition 16 would harm advantaged ingroup resource access predicted reduced support [*b* = 0.30, *t*(643) = 6.86, *P* < 0.001, 95% CI [0.21, 0.38]]. Although most participants (84.7%) did not change their voting preference between T1 and T2 (see Materials and Methods), for those who did, a one-unit decrease in perceived ingroup resource access predicted 0.39 greater log odds of changing a yes vote to a no vote (SE = 0.10, *P* < 0.001). However, a one-unit change in perceived ingroup resource access was not significantly associated with an increase in the log odds of changing from a no to a yes vote (log odds = 0.19; SE = 0.15, *P* = 0.20). Again, these effects remained significant even when controlling for ideological variables collected at T1 (see tables S17 and S21).

Although affirmative action policies, in practice, do not necessarily reduce advantaged groups’ access to positions (*48*, *49*), we cannot definitively state what the objectively correct response should have been for our participants. Therefore, we do not label these participants’ responses as a misperception as in our other studies. In addition, this study does not precisely distinguish people of different Asian ethnic backgrounds (e.g., Korean, Vietnamese, Filipino, Indian, etc.). The extent to which people of different Asian backgrounds perceive themselves to be advantaged in this context is dependent on a host of factors, and heterogeneity is to be expected (*50*, *51*). To begin to acknowledge this heterogeneity, we include subgroup analyses by participant race/ethnicity in the Supplementary Materials (see tables S14 to S16, S18 to S20, and S22 to S24).

Despite these limitations, this study does lend ecological validity to our hypotheses. White, East Asian, and South Asian California voters perceived Proposition 16 as harmful to their group’s ability to access employment and educational opportunities, and this perception predicted reduced support and reduced voting preference for this equality-enhancing policy. This perception of reduced resource access also appears to be distinct from well-studied forms of antiegalitarianism. Perceived harm to resource access predicted support and voting preference more strongly than explicit prejudice, opposition to equality, system-justifying beliefs, global zero-sum beliefs, or political orientation. Our effects also emerged despite a disproportionately liberal participant sample, foreshadowing the fact that Proposition 16 would ultimately be struck down despite a majority liberal California electorate (*52*). Collectively, these findings exemplify that the observed effect is uniquely and, perhaps, primarily predictive of behavior in real-world settings and is not merely explained by antiegalitarianism.

With consistent results across studies 1 to 6, we aimed to answer three lingering questions. First, would the same results emerge when assigning participants to a novel and arbitrary advantaged group, suggesting that our effect informs social identity theory? Second, does the misperception that equality-enhancing policies harm the ingroup causally predict lower behavioral support for policies? Third, could an intervention to improve deliberative decision-making undo this misperception? Namely, would the observed effects persist if we provided economic incentive to vote for equality or asked participants to jointly consider unharmful equality-enhancing policies with truly harmful ones?

Studies 7 and 8 used a novel-groups paradigm in which we arbitrarily assigned participants to a fictitious advantaged group. This paradigm allowed us to rule out the influence of prevailing attitudes and idiosyncratic relationships between existing advantaged and disadvantaged groups in America. As in studies 1 to 5, we designed equality-enhancing policies that either would not change advantaged group resource access or would increase it. We specifically created these new policies to account for the alternative hypothesis that our effects are due to participants’ expectation of unintended spillover effects (e.g., inflation reducing resource value). Participants’ support or opposition toward these policies also determined the expected financial value of their participation. Whereas many previous novel group studies demonstrate that people prefer to allocate more resources to the ingroup (*19*, *53*), we tested the extent to which resource allocation is predicted by the misperception that equality harms the ingroup. In sum, this paradigm allowed us to test how advantaged group members’ misperceptions of harm could lead to the rejection of more equitable outcomes.

### Study 7

We drew inspiration from study 3 and predicted that advantaged group members would misperceive a policy that increased equality and benefited everyone (i.e., win-win equality-enhancing) as more harmful to their resource access than a policy that both worsened inequality and hurt everyone (i.e., lose-lose inequality-enhancing). Unlike study 3, which described equality as indirectly benefitting everyone, equality directly increased participants’ resource access in study 7. We thereby tested whether participants would still misperceive greater equality as harming resource access even when directly financially incentivized to vote in favor of it.

We randomly assigned 496 U.S. participants to one of two conditions using a 2-cell between-subjects design (https://osf.io/mntjw/). The experiment ostensibly involved two teams, the Rattlers and the Eagles, completing a problem-solving challenge (*54*, *55*). We told participants that they would be assigned to a team according to a personality test. In reality, the test was bogus, and we assigned all participants to the Rattlers team. We informed participants that Rattlers had received more bonuses (126 bonuses) than had Eagles (79 bonuses) in previous weeks. We also informed all participants that we recruited a set number of participants each week. This detail ensured that any increase in the number of bonuses could only logically be interpreted as increasing one’s odds of receiving a bonus and, thus, as increasing the expected value of participation.

We then asked participants to consider a new proposal for the bonus distribution procedure that week. In the win-win equality-enhancing condition, participants read a proposal that would allocate bonuses more equally by providing 50 additional bonuses to Eagles and 5 additional bonuses to Rattlers (i.e., the advantaged ingroup) than in previous weeks. This policy benefitted both groups and made the number of Eagles bonuses nearly equal to the number of Rattlers bonuses (see the Supplementary Materials). In the lose-lose inequality-enhancing condition, participants read a proposal that would allocate bonuses according to whichever group received more bonuses in the previous week and, thus, provided 50 fewer bonuses to Eagles and 5 fewer bonuses to Rattlers. This policy harmed everyone and made the bonus distribution even more unequal. Participants then indicated perceived ingroup resource access, policy support, and their vote for or against the proposal. We truthfully informed participants that their vote would determine how many bonuses we provided that week. Therefore, participants’ odds of receiving a bonus increased if the equality-enhancing policy received more votes, and their odds decreased if the inequality-enhancing policy received more votes.

**Fig. 6.**
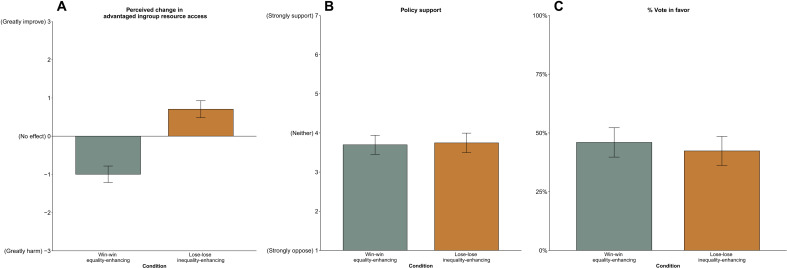
Study 7. Rattlers’ (**A**) perceived changes in advantaged ingroup resource access and (**B**) policy support toward and (**C**) frequency of voting in favor of an equality-enhancing procedure and inequality-enhancing procedure, study 7 (*n* = 496). Error bars indicate 95% CIs around the mean.

As predicted, participants misperceived the win-win equality-enhancing policy (*M* = −1.00, SD = 1.72) as more harmful to their chances of receiving a bonus than the lose-lose inequality-enhancing policy [*M* = 0.71, SD = 1.76, *t*(494) = −10.89, *P* < 0.001, 95% CI [−2.01, −1.39]]. Notably, we found no significant difference in support (*M* = 3.70, SD = 1.94) or voting for (45.98%) the policy that made participants better off and increased equality compared to support [*M* = 3.75, SD = 2.00, *t*(493) = −0.28, *P* = 0.78, 95% CI [−0.40, 0.30]] or voting for the policy that made them worse off and exacerbated inequality [42.34%; χ^2^(1, *n* = 496) = 0.52, *P* = 0.47] (see Fig. 6).

**Fig. 7.**
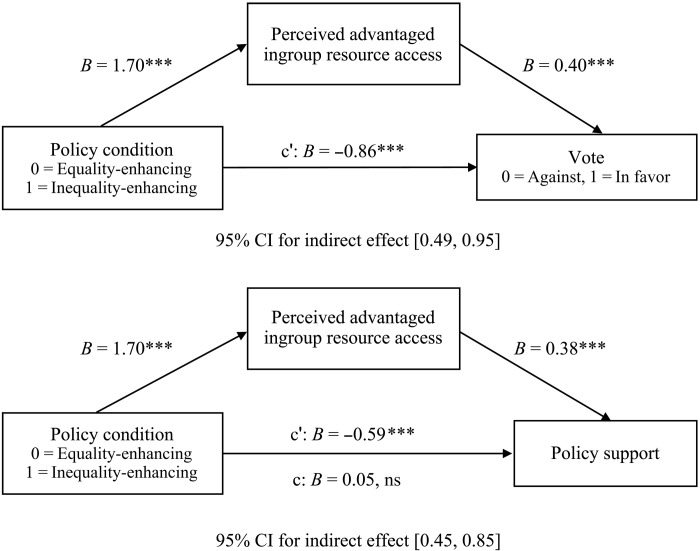
Perceived advantaged ingroup resource access mediated the effect of policy condition on vote and policy support. Mediation analysis was conducted using Hayes PROCESS Model 4 (10,000 bootstrapped samples). Mediation was observed if the bootstrapped 95% CIs of the indirect effect did not contain zero. c, total effect model; c′, direct effect model. Total effect model (c) is not available with dichotomous outcome variables. ***P < 0.001; ns, not significant.

Evidence suggests that participants’ misperceptions regarding their chance of receiving a bonus explained this pattern of support. Mediation analyses showed that perceived advantaged ingroup resource access accounted for a significant portion of the variance of the relationship between policy condition and support (*b* = 0.64, SE = 0.10, 95% CI [0.45, 0.85]), as well as the relationship between policy condition and vote (*b* = 0.69, SE = 0.12, 95% CI [0.49, 0.95]; see Fig. 7). Each one-unit increase in perceived advantaged ingroup harm predicted 9.32% lower policy support and 59.9% lower chance of voting for the policy, controlling for policy condition. Thus, the more participants misperceived the policy as harming their group’s ability to receive a bonus, the less they supported the policy and the more likely they were to vote against it. Although our analysis cannot rule out other untested mediators, the observed relationship provides evidence that advantaged group members are opposed to equality-enhancing policies that benefit them because they misperceive them as harmful (*56*).

### Study 8

Although direct financial incentive did not erase misperceptions of harm, we attempted one last intervention strategy. Past work suggests that simultaneously presenting unharmful equality-enhancing policies alongside truly harmful ones (i.e., joint evaluation) might produce more accurate perceptions compared to considering a single unharmful policy by itself (i.e., separate evaluation). This intervention strategy has been shown to activate more deliberative decision-making processes (*36*, *57*). While separate evaluation often leads people to focus on whether the ingroup or outgroup receives more, joint evaluation encourages people to focus on whether each option makes their ingroup better or worse off. As a result, joint evaluations often lead to more economically rational policy choices, even when another group benefits relatively more than one’s own group (*58*). This track record has led behavioral scientists to argue that joint evaluations should be used in deciding between different public policies (*59*). We tested whether advantaged group members would perceive an unharmful equality-enhancing policy more accurately when presented alongside a truly harmful equality-enhancing policy compared to when the same unharmful equality-enhancing policy is presented in isolation.

We recruited 492 U.S. participants into an experiment with a similar paradigm as study 7. Participants were again always assigned to the advantaged Rattlers team and informed that we recruited a set number of participants each week. This time, we also explicitly told participants that we randomly selected bonus recipients, ensuring that any bonus advantage held by the Rattlers was explicitly unearned. We then assigned participants to view either two joint policy options for allocating bonuses more equally across teams or a single policy option. In the joint policy condition, we made explicit that there are two ways to achieve equality: increasing resources to disadvantaged group members (option A) or decreasing resources to advantaged group members (option B). Specifically, we told participants that option A would provide 50 additional bonuses to Eagles and not change the number of bonuses to Rattlers (i.e., the unharmful policy). Option B would provide 50 fewer bonuses to Rattlers and not change the number of bonuses to Eagles (i.e., the harmful policy). In the single policy condition, participants read only the unharmful policy (https://osf.io/3h4ps/).

**Fig. 8.**
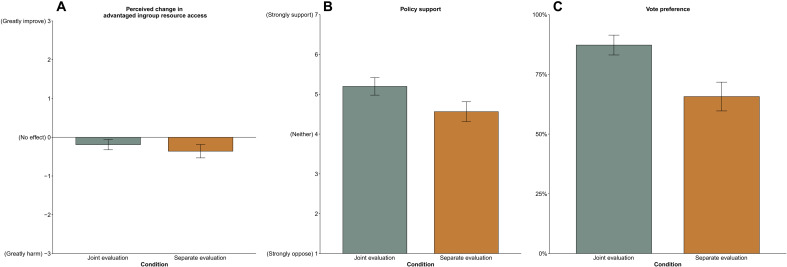
Study 8. Rattlers’ (**A**) perceived changes in advantaged ingroup resource access and (**B**) policy support toward and (**C**) frequency of voting in favor of an equality-enhancing bonus allocation procedure jointly or separately (*n* = 492). Error bars indicate 95% CIs around the mean.

Participants indicated greater support (*M* = 5.20, SD = 1.75) and voting (87.25%) in favor of the unharmful equality-enhancing policy when presented jointly than when presented as a single policy option [single support: *M* = 4.56, SD = 1.96; *t*(486) = 3.77, *P* < 0.001, 95% CI [0.30, 0.97]; single vote: 65.70%; χ^2^(1, *n* = 493) = 30.79, *P* < 0.001]. However, participants in the joint policy condition did not view the unharmful policy as significantly less harmful to their chances of receiving a bonus than in the single policy condition [*t*(491) = 1.52, *P* = 0.13, 95% CI [−0.049, 0.39]]. Troublingly, participants misperceived the unharmful equality-enhancing policy as reducing their chances of a bonus regardless of whether it was presented as a joint (*M* = −0.19, SD = 1.07) or as a single option (*M* = −0.36, SD = 1.37; see Fig. 8). Thus, although joint decision-making improved attitudinal and behavioral support for unharmful equality-enhancing policies, participants’ misperception that equality harms advantaged group resource access persevered.

## DISCUSSION

While most Americans believe that the United States must take steps to achieve greater equality, policies meant to do just that are often construed as discriminatory or threatening to members of the advantaged group (*2*). Unfortunately, these objections persist despite evidence that the pursuit of equality need not be zero-sum; equality can, and often does, buoy the fates of all members of a society (*44*). The present paper identifies what may be a primary roadblock in creating a more equitable society.

Across nine studies, we show that advantaged group members misperceive equality-enhancing policies as harming their access to resources, even when the policies do no such thing. We identify this misperception across various inequality contexts (e.g., mortgage lending, salary, and hiring), various group boundaries (e.g., race, gender, disability, and arbitrary group distinctions), and different types of resources (e.g., money and jobs). Advantaged group members also misperceive policies that maintain the status quo or magnify inequality as improving their resource access, even when the policies actually leave them no better off. This tendency for advantaged group members to think that equality necessarily incurs a cost to their group lingered even when equality-enhancing policies mutually benefited disadvantaged and advantaged groups in a win-win fashion. That is, advantaged group members misperceive having greater inequality and fewer resources available to their group as more advantageous than having greater overall resources that were shared more equally.

We also find that these harm perceptions can have profound implications for individuals’ attitudinal and behavioral opposition to policies that promote equality. During the 2020 election, California Proposition 16 proposed relegalizing the use of affirmative action policies in the public sector. We find that the more white and Asian voters perceived that California Proposition 16 would harm their access to resources, the less likely they were to express support or vote for Proposition 16, independent of their political leaning. Moreover, we find that behavioral opposition occurs even when harm perceptions are objectively false and the effects of equality-enhancing policies are unambiguously positive. In an experimental setting, advantaged group participants were just as likely to vote for an inequality-enhancing policy that financially harmed them as they were to vote for an equality-enhancing policy that financially benefitted them. These studies suggest that real-world opposition to equality is likely caused by unduly negative perceptions of policies that could reduce inequality and unduly positive perceptions of policies that exacerbate it.

Our evidence sheds new light on foundational theories of social psychology. Social identity theory posits that people tend to prefer relatively greater amounts of resources be allocated to their ingroup than to an outgroup (*19*). We find that unequal resource allocation is predicted by the misperception that reductions of relative advantage (i.e., greater equality) necessarily harm advantaged groups in absolute terms. This finding also serves as a novel explanation for why societally advantaged individuals are motivated to protect and maintain their ingroup’s dominant position in the society (*28*, *60*). Advantaged group members not only feel threat at losing superior status but also misperceive that equality reduces their access to resources even when access explicitly goes unchanged, or even increases. Our findings may be a key reason why ingroup favoritism proves so pernicious, both in the laboratory and real world.

In further support of our assertions, the observed harm misperceptions are not driven by ideological opposition to equality. We measured ideological beliefs across all studies (i.e., political orientation, social dominance, explicit prejudice, system justification, global zero-sum beliefs, and status and symbolic threats) and found no strong or consistent interactions between ideology and conditions. Our effects also remained significant even when controlling for all ideological beliefs (see tables S2 to S13). Illustrating the real-life implications of this, we found that the perception that California Proposition 16 was harmful to their racial group independently predicted white and Asian voters’ opposition to the policy, even more so than any ideology we measured (see tables S14 to S24). Our final two studies also confirm this takeaway, both by using novel groups to render political context and extant group prejudices irrelevant and by replicating that misperceptions of equality-enhancing policies predict opposition when controlling for participants’ ideological beliefs (see tables S25 and S27). Collectively, these findings suggest that advantaged group members’ misperceptions of harm operate alongside antiegalitarian ideologies and extant social narratives and group dynamics to undermine support for equality.

The misperception that equality is harmful is stubbornly persistent, resisting both reason and incentivization. Specifically, this misperception prevails when resource scarcity concerns are addressed by framing resources as unlimited (study 4) or when participants are directly informed that equality-enhancing policies would not limit the advantaged groups’ access to resources (study 5). The irrationality of this misperception is underscored by the fact that it persists when participants are encouraged to think more deliberatively about policies by jointly presenting truly unharmful and harmful equality-enhancing policies side by side (study 8). Perhaps, then, it is no surprise that participants continue to misperceive equality as harmful even when it financially benefits them (study 7). These grim results suggest that perceiving equality as harmful to advantaged groups is a powerful heuristic (*61*).

Practically, our findings speak to how remarkably widespread misperceptions of inequality are in, and perhaps beyond, American society. Past work has shown that historically advantaged group members have negative perceptions of particular policies or specific societal changes. For example, researchers have shown that white Americans see losing majority status as threatening and anxiety-inducing (*62*, *63*) or that white Americans report that diminishing anti-Black bias is associated with greater anti-white bias (*34*). Ours suggests that these findings may flow from a common source: the persistent and pernicious misbelief that equality itself is inherently zero-sum. This interpretation dovetails with growing evidence that individuals misperceive other aspects of inequality as well. For instance, Americans vastly underestimate racial economic inequality, optimistically perceiving the Black-white wealth gap as smaller than it actually is (*64*, *65*). Together, this emerging body of work suggests that inequality may endure primarily because people fundamentally misunderstand the reality of the disparities weighing down their society.

The results of the present research are cause for a mix of optimism and pessimism. Two of our manipulations significantly reduced perceptions that equality-enhancing policies necessarily harm advantaged groups. First, while advantaged group members misperceived a policy as harming resource access when it resolved an intergroup disparity (e.g., reducing a pay gap between men and women), participants accurately perceived the same policy as unharmful when it reduced inequality within their ingroup (e.g., reducing a pay disparity between men; study 2). On one hand, this finding might elicit a glimmer of hope for policy-makers who aim to circumvent negative responses to equality policies by eliciting a shared identity (e.g., “we are all American”). On the other hand, the evocation of shared—or superordinate—identities has limited effectiveness precisely because it depends on suppressing the importance of subordinate identities that people highly value (e.g., racial or gender identity) (*66*). Such limitations might be why highly racially homogeneous nations tend to implement more redistributive policies than more racially heterogeneous ones (*42*, *43*). The second manipulation that significantly reduced perceptions of harm was our assurance that resources for an equality-enhancing policy and advantaged group members’ access to those resources were both unlimited. It is worth underlining the obvious here: These situations are exceedingly rare, perhaps nonexistent. Moreover, advantaged group members who were given this assurance nonetheless strikingly continued to see equality-enhancing policies as harming their resource access. Even as these two studies suggest possible avenues for improving perceptions of equality, the feasibility and effectiveness of each are limited.

In a similarly ambivalent fashion, the results of study 8 foreshadow how one’s support for equality may sometimes be more positive than perceptions of how equality will affect one’s group. Specifically, although a joint evaluation intervention (*57*–*59*) successfully improved support and voting for an equality-enhancing policy, it did not reduce participants’ misperceptions of harm. Future research should explore whether the tenacity of this misperception could be the cause of some familiar forms of backlash, where progress outstrips public sentiment. In Silicon Valley, for example, diversity efforts have been increasingly implemented but have also been met with outcries from predominantly white employees that such policies are discriminatory toward them (*67*). This harm perception may also explain the prevalence of “window-dressing” policies. Organizations and policy-makers often brand themselves as valuing equality while simultaneously adopting policies that are merely symbolic and do little more than preserve the status quo (*68*). For instance, while legislation to protect the right to vote for historically disenfranchised groups—especially Black Americans—continues to stall in Washington D.C., symbolic concessions, such as making Juneteenth a federal holiday, were broadly supported and swiftly passed (*69*). In effect, outspoken support for equality was more present than the mettle to actually increase it. Our final study adds perspective to these historical passages. Even when advantaged group members are presented with two available options for achieving equality—either lifting up those at the bottom (at no cost) or dragging down those at the top—they stubbornly view either option as a sacrifice. So long as the interests of the advantaged group are held in higher consideration than the well-being of the disadvantaged, our studies suggest that existing levels of intergroup inequality are unlikely to be effectively addressed.

These findings are but a first step toward understanding zero-sum misperceptions in the context of equality. Future research would benefit from understanding how people in disadvantaged groups perceive equality-enhancing policies. While some research indicates that everyone is susceptible to perceiving the world through a zero-sum lens (*25*, *38*), it is possible that disadvantaged group members are motivated to construe equality-enhancing policies as non–zero-sum. Recent work shows that, whereas white Americans believe that they are hurt by university diversity policies that mutually benefit white and non-white applicants, Black Americans accurately see these polices as helping everyone (*12*). This divergence may have something to do with the fact that people frequently believe that others gain at one’s own expense but that one’s own gains do not come at the expense of others (*24*, *70*). However, it also remains an open question how disadvantaged group members perceive policies that reduce societal resources but increase equality or, conversely, polices that increase inequality while increasing societal resources. If disadvantaged groups, like advantaged groups, attend primarily to changes in relative advantage, then perverse policy incentives may also exist for people at the bottom rungs of society. Thus, while zero-sum thinking is likely a general phenomenon, group membership may shape the circumstances under which this lens is applied.

A critical next step for future research concerns how the negative effects of zero-sum equality perceptions can be averted or how we can make progress toward equality despite these misperceptions (*71*). Research on intergroup conflict and coalition building may provide a path forward. For instance, depending on others to achieve a common goal can strengthen cooperation between groups (*72*, *73*). Related work in negotiations illustrates that coalitions lead negotiators to identify compatible interests more readily (*74*). However, social inequality involves the critical complication of building coalitions between groups with unequal status, power, and dominance in society (*75*, *76*). Future research must therefore examine how advantaged groups can be convinced to relinquish their relative advantages even as doing so inherently feels similar to a material concession. Future research can thereby highlight new ways to capitalize on, rather than merely cope with, increasing societal diversity.

The current research provides sobering insight into the prevalence and consequences of misperceiving equality as zero-sum. As inequality in America, and around the world, continues to constrain the economic, psychological, and physical well-being of both the fortunate and unfortunate, we identify a reason why it persists—advantaged group members’ misperception that equality necessarily harms them and inequality necessarily benefits them. Our findings also return us to an enduring moral quandary that has echoed unresolved by American society: “If you can only be tall because somebody’s on their knees, then you have a serious problem” (*77*).

## MATERIALS AND METHODS

Studies 1 to 5 shared the same methodological template, as did studies 7 and 8. In every study, we measured political orientation, social dominance orientation (*37*), global zero-sum beliefs (*38*), system-justifying beliefs (*39*), and explicit prejudice (*40*) on a 1 (very unfavorable/strongly disagree) to 7 (very favorable/strongly agree) scale. See the Supplementary Materials for the complete materials used in each study.

### Study 1a

We recruited 597 white (non-Hispanic) U.S. citizens from Prolific to complete our study in exchange for $1.30. As preregistered (https://osf.io/9y876/), we excluded three participants for providing nonsensical responses to our open-response question (“Using at least 1-2 complete sentences, tell us why you would vote for or against the new bonus procedure.”). This resulted in a final sample of 594 participants (51.85% men, 46.29% women, and 1.85% nonbinary; *M*_age_ = 38.99, SD_age_ = 14.27; 62.63% politically liberal).

After providing informed consent, participants reported their ethnicity, age, gender, education level [self and parent(s)], employment status, disability status, and criminal history (see the Supplementary Materials). We next showed participants three vignettes randomly selected from a pool of six. Three vignettes described a monetary disparity and three described a representational disparity. Each vignette described a different inequality context to ensure that our findings were not specific to any particular disparity or group (e.g., “According to a recent report, in 2018, White homebuyers received roughly $386.4 billion in mortgage loans from banks, while Latino buyers only received around $12.6 billion in mortgage loans overall.”). The six contexts were as follows (advantaged group | disadvantaged group): mortgage lending discrimination (white homebuyers | Latino homebuyers), gender pay gap (men | women), disability employment gap (job seekers without a disability | job seekers with a disability), ex-prisoner employment gap (people with no criminal history | people with criminal history), start-up funding discrimination (white entrepreneur | Black entrepreneur), and university admission disparities (continuing-generation students|first-generation students). Participants only saw contexts for which they identified as members of the advantaged group, based on their responses to the initial demographic questions. For instance, only male participants viewed materials related to the gender pay gap.

We randomly assigned participants to one of three between-subjects conditions. In the equality-enhancing policy condition, participants read about policies that proposed to increase the disadvantaged group’s resource access and not change the advantaged group’s resource access, which would therefore reduce the disparity:

“Several banks propose increasing the total amount of mortgage loans to Latino homebuyers by $7.3 billion and not changing the total amount of mortgage loan funding to White homebuyers. Ultimately, these banks predict that this proposal will narrow the gap in mortgage loans between Latino and White homebuyers over the next year.”

In the status quo condition, participants read that no policy was proposed to address the disparity:

“However, several banks propose not changing mortgage loan funding over the next year.”

In the inequality-enhancing condition, participants read about policies that proposed to decrease the disadvantaged group’s resource access and not change the advantaged group’s resource access, which would therefore worsen the disparity:

“Several banks propose decreasing the total amount of mortgage loans to Latino homebuyers by $7.3 billion and not changing the total amount of mortgage loan funding to White homebuyers. Ultimately, these banks predict that this proposal will widen the gap in mortgage loans between Latino and White homebuyers over the next year.”

Participants indicated how each policy proposal would affect the advantaged ingroup’s resource access [“How do you think the proposed changes will affect [advantaged groups’] chances of [receiving resources]?”; 7-point Likert-scale with meaningful anchors: −3 (greatly harm), 0 (no effect), and +3 (greatly improve)].

### Study 1b

We recruited 401 white (non-Hispanic) U.S. citizens from Prolific to complete our study in exchange for $1.30. As preregistered (https://osf.io/9q35s/), we excluded two participants for not self-reporting their ethnicity as white (non-Hispanic). This resulted in a final sample of 399 participants (38.60% men, 60.15% women, and 1.25% nonbinary; *M*_age_ = 38.87, SD_age_ = 14.13; 60.55% politically liberal).

We again showed participants information regarding three of six possible vignettes. Unlike study 1a, the monetary vignettes were adapted so that all six vignettes described a representational disparity. We randomly assigned participants to one of two between-subjects conditions from study 1a: either an equality-enhancing policy condition or an inequality-enhancing policy condition.

Participants indicated how each policy proposal would affect the advantaged ingroup’s resource access using a newly worded dependent variable [“How do you think this proposal will affect [advantaged group’s] chances of [receiving resources] next year compared to their chances of [receiving resources] in the past?”; 7-point Likert-scale: −3 (greatly harm), 0 (no effect), and +3 (greatly improve)]. We measured group status threat and symbolic threat using three items matched to each policy vignette that participants read (*32*, *78*).

### Study 2

We recruited 396 white (non-Hispanic) men through Prolific to complete our study for $1.30. As preregistered (https://osf.io/efz6j/), we excluded nine participants for providing nonsensical responses to our open-response question. This resulted in a final sample of 387 (*M*_age_ = 38.78, SD_age_ = 13.14; 52.85% politically liberal).

We showed participants three vignettes but limited the vignette pool to the three that involved monetary disparities: mortgage lending discrimination (white homebuyers | Latino homebuyers), gender pay gap (men | women), and start-up funding discrimination (white entrepreneur | Black entrepreneur). We then adapted these vignettes to describe each disparity in terms of the average amount of resource received by members of each group.

We employed a 2-cell (intergroup equality-enhancing versus ingroup equality-enhancing policy) between-subjects design. Participants in the intergroup equality-enhancing policy condition read about a policy that would enhance equality between an advantaged ingroup and an equally deserving disadvantaged outgroup:

“According to a 2019 report, while White homebuyers in a neighborhood received an average of $273,000 in mortgage loans from banks, comparable Latino homebuyers in the same neighborhood received an average of $249,000 in mortgage loans. There was no available explanation for this gap.”

Participants in the ingroup equality-enhancing policy condition read about the same disparity, but both advantaged and disadvantaged parties were members of participants’ ingroup:

“According to a 2019 report, while White homebuyers in a neighborhood received an average of $273,000 in mortgage loans from banks, comparable White homebuyers in the same neighborhood received an average of $249,000 in mortgage loans. There was no available explanation for this gap.”

Participants in both conditions then read about a policy that proposed providing enough resources to the disadvantaged group to make the average amount of resources exactly equal (e.g., “Several banks propose increasing mortgage loans by an average of $24,000 to the group of White homebuyers who tend to receive less and not changing the total amount of mortgage loan funding to the other White homebuyers. Ultimately, these banks predict that this proposal will erase the gap in mortgage loans between these homebuyers over the next year”). Participants reported perceived advantaged group resource access using the same measure as in study 1a.

### Study 3

We recruited 399 white (non-Hispanic) U.S. citizens through Prolific to complete our study for $1.30. As preregistered (https://osf.io/t4knh/), we excluded six participants for providing nonsensical responses to our open-response question. This resulted in a final sample of 393 (48.09% men, 49.87% women, and 2.04% nonbinary; *M*_age_ = 40.21, SD_age_ = 14.60; 60.56% politically liberal).

We again showed participants three vignettes out of the pool of six used in study 1a. We used a 2-cell (societally beneficial equality-enhancing policy vs. societally harmful inequality-enhancing policy) between-subjects design. In the equality-enhancing condition, participants were explicitly told that the policy would stimulate broad benefits for society:

“Several banks propose increasing the total amount of mortgage loans to Latino homebuyers by $7.3 billion and not changing the total amount of mortgage loan funding to White homebuyers. Ultimately, these banks predict that this proposal will narrow the gap in mortgage loans between Latino and White homebuyers over the next year. These banks stated that this policy will have the additional effect of stimulating greater mortgage investment nationwide, increasing the total benefits for homebuyers of all racial groups.”

In the inequality-enhancing condition, participants were told the policy would incur broad societal costs:

“Several banks propose decreasing the total amount of mortgage loans to Latino homebuyers by $7.3 billion and not changing the total amount of mortgage loan funding to White homebuyers. Ultimately, these banks predict that this proposal will widen the gap in mortgage loans between Latino and White homebuyers over the next year. These banks stated that this policy will have the additional effect of reducing mortgage investment nationwide, decreasing the total benefits for homebuyers of all racial groups.”

Participants reported perceived advantaged ingroup resource access as in study 1a.

### Study 4

We recruited 399 white (non-Hispanic) U.S. citizens through Prolific to complete our study in exchange for $1.30. As preregistered (https://osf.io/x5etb/), we excluded six participants for providing nonsensical responses to our open-response question. This resulted in a final sample of 393 (50.64% men, 47.33% women, and 2.03% nonbinary; *M*_age_ = 37.84, SD_age_ = 12.92; 63.87% politically liberal).

We showed participants three vignettes out of the pool of six used in study 1a. We used a 2 (equality-enhancing policy vs. inequality-enhancing policy) x 2 (limited resources vs. unlimited resources)” between-subjects design. In the limited resources condition, we told participants the resources for the proposal were finite (e.g., “These banks reported there will be no change in profits for many years, and they will need to reorganize their budgets to fund these mortgage loans”). In the unlimited condition, we told participants that more than enough resources were available to realize the policy (e.g., “These banks reported there have been large and consistent increases in profits that will continue for many years, allowing them to fund mortgage loans for as many people as they want”). Participants reported perceived advantaged ingroup resource access using the same measure as in study 1a.

### Study 5

We recruited 403 white (non-Hispanic) U.S. citizens through Prolific to complete our study in exchange for $1.30. As preregistered (https://osf.io/vp3nq/), we excluded four participants for providing nonsensical responses to our free response question. This resulted in a final sample of 399 (38.34% men, 60.65% women, and 1.00% nonbinary; *M*_age_ = 37.72, SD_age_ = 13.72; 63.16% politically liberal).

We showed participants three vignettes out of the pool of six used in study 1a. We used a 2-cell (limited access versus unlimited access) between-subjects design. Both conditions described equality-enhancing policies. We told participants in the limited access condition that the policy would limit the advantaged group’s access to the resource (e.g., “These banks reported there will be no change in profits for many years, and they will need to reorganize their budgets to fund these mortgage loans. Therefore, these banks will provide a limited number of mortgage loans, and the proposal will cause some White applicants to not receive funding”). We told participants in the unlimited access condition that the policy would allow for anyone in the advantaged group to receive the resource (e.g., “These banks reported there have been large and consistent increases in profits that will continue for many years, allowing them to fund mortgage loans for as many people as they want. Therefore, anyone who wants a mortgage loan can receive one”). Participants also completed an attention check, entering the exact amount by which the policy would change resources to each group (e.g., “By how much are banks planning to increase mortgage funding to White [Latino] homebuyers?”). Participants reported perceived advantaged ingroup resource access as in study 1a.

### Study 6

We recruited 1016 California residents (517 non-Hispanic white and 499 Asian) from Prolific to participate in the time 1 (T1) survey, fielded between 12 October 2020 and 19 October 2020. As preregistered, we excluded 76 people who did not identify as white (non-Hispanic), 38 people who did not identify as Asian, 70 people who were not registered California voters, and 11 people who neither identified as white nor Asian nor were registered to vote in California. This resulted in a final T1 sample of 821 participants (411 Asian participants; 52.7% men and 46.4% women; *M*_age_ = 31.70, SD_age_ = 12.33). Six days later, we invited this sample of participants to complete the time 2 (T2) survey, fielded between 27 October 2020 and 2 November 2020. A total of 323 white (78.6% response rate; 48.3% men and 49.8% women; *M*_age_ = 37.85, SD_age_ = 14.61) and 325 Asian participants (79.3% response rate; 56.6% men and 43.1% women; *M*_age_ = 27.12, SD_age_ = 8.62) completed the follow-up survey. We preregistered both surveys (https://osf.io/x5w87/).

After providing consent, participants in both surveys read the summary of California Proposition 16 as presented on the official California ballot:

“Proposition 16. Allows Diversity as a Factor in Public Employment, Education, and Contracting Decisions, Legislative Constitutional Amendment. Permits government decision-making policies to consider race, sex, color, ethnicity, or national origin to address diversity by repealing constitutional provision prohibiting such policies. Fiscal Impact: No direct impact on state and local entities. The effects of the measure depend on the future choices of state and local government entities and are highly uncertain.”

After reading the Proposition 16 summary, participants indicated perceived advantaged ingroup resource access: “How do you think Proposition 16 will affect non-underrepresented people’s (e.g., non-Hispanic White, Asian/Asian-American) chances of gaining placement in public employment, public education, and public contracting positions in CA?” (−3 = greatly harm; 0 = no effect; +3 = greatly improve). Participants indicated policy support (“Overall, how much do you oppose or support Proposition 16?”; 1 = strongly oppose; 7 = strongly support) and their vote preference (“If you were voting today, how would you vote for CA Proposition 16?”; 0 = no; 1 = yes). Participants also reported explicit prejudice [“To what degree is your opinion of minorities (race, sex, color, ethnicity, and national origin) favorable or unfavorable?”]. We measured the same ideologies from study 1a during T1. We measured social, economic, and overall political orientation. These three items were strongly correlated (α = 0.92), and the pattern of results is identical when using a composite item in analyses. For simplicity, we report the analyses using only overall political orientation. At T2, we recollected perceived advantaged ingroup resource access, policy support, and vote preference as in T1. Between T1 and T2, most participants (*n* = 546; 84.7%) did not change their vote, whereas 9.15% (*n* = 59) participants changed their vote from a yes to a no and 6.20% (*n* = 40) participants changed their vote from a no to a yes.

### Study 7

We recruited 500 U.S. citizens from Prolific to participate in this study in exchange for $0.85. As preregistered (https://osf.io/mntjw/), we excluded four participants for providing nonsensical responses to our open-response question (“Using at least 1-2 complete sentences, tell us why you would vote for or against the new bonus procedure.”). This resulted in a final sample of 496 participants (47.98% men, 49.60% women, and 1.61% nonbinary; 49.60% white, 20.16% Asian, 13.91% Black, 7.86% Latinx, and 8.47% other; *M*_age_ = 33.20, SD_age_ = 10.81; 65.32% politically liberal).

We told participants that a personality test would assign them one of two teams—the Rattlers or the Eagles—and they would then complete a series of problem-solving tasks (*52*, *53*). However, this personality test was not actually used to determine team assignment. Instead, we assigned all participants to the Rattlers team. After learning about their team assignment, we told participants: “After teams complete the tasks each week, we select participants to receive a bonus for their efforts. In previous weeks, Rattlers have been selected to receive bonuses more often than Eagles.” We then asked participants to consider a new proposal for the bonus distribution procedure that week and randomly assigned them to one of two between-subjects conditions. In the win-win equality-enhancing policy condition, we told participants: “We are implementing a new procedure to allocate bonuses more equally across the groups. With this change, we will provide 50 additional bonuses to Eagles and only 5 more bonuses to Rattlers.” In the lose-lose inequality-enhancing policy condition, we told participants: “We are implementing a new procedure to allocate more bonuses to whichever group previously received more bonuses. With this change, we will provide 50 fewer bonuses to Eagles and only 5 fewer bonuses to Rattlers.” We also clearly indicated the number of bonuses each group was expected to receive before and after the new procedure in a figure shown alongside the text. We truthfully informed participants that their vote would determine how many bonuses we provided that week. Therefore, participants’ odds of receiving a bonus were increased if the equality-enhancing policy was passed and were decreased if the inequality-enhancing policy was passed.

After reading the policy, participants indicated how the proposal would affect their advantaged group’s resource access (“How does the new bonus procedure affect Rattlers’ chances of receiving a bonus?”; 7-point Likert scale from −3 = greatly harm to +3 = greatly improve), policy support (“How much do you oppose or support the new bonus procedure?”; 7-point Likert scale from 1 = strongly oppose to 7 = strongly support), and voting (“Should the new bonus procedure be implemented?”; 0 = no; 1 = yes). These three questions were presented in a counterbalanced order. As a manipulation check, participants then reported how much they identified with the Rattlers and Eagles: “I value the Rattlers [Eagles] group,” “I like the Rattlers [Eagles] group,” and “I feel connected to the Rattlers [Eagles] group” on a 0 to 100 slider scale. We averaged the three ingroup (i.e., Rattlers) and outgroup (i.e., Eagles) items to form ingroup identification (α = 0.86) and outgroup identification measures (α = 0.88). Participants more strongly identified with the Rattlers (*M* = 75.42, SD = 19.05) than the Eagles [(*M* = 48.08, SD = 21.82); *t*(495) = −22.79, *P* < 0.001], indicating that our group assignment procedure was successful.

### Study 8

We recruited 502 U.S. citizens from Prolific to participate in this study in exchange for $0.85. As preregistered (https://osf.io/3h4ps/), we excluded nine participants for providing nonsensical responses to our open-response question (“Using at least 1-2 complete sentences, tell us why you would vote for or against the new bonus procedure.”). This resulted in a final sample of 492 participants (46.65% men, 51.52% women, and 1.42% nonbinary; 50.3% white, 20.89% Asian, 12.17% Black, 9.13% Latinx, and 7.51% other; *M*_age_ = 34.37, SD_age_ = 12.16; 61.59% politically liberal).

This study largely followed the study 7 procedure. In the current study, all participants were informed that, each week, we recruited a set number of participants and randomly selected bonus recipients. We also used two new between-subjects conditions. In the joint policy condition, participants read:

“After teams complete the tasks each week, we randomly select participants to receive a bonus for their efforts. In previous weeks, Rattlers have been selected to receive bonuses more often than Eagles. We are considering implementing a new procedure to allocate bonuses more equally across the groups. We are considering two options: Option A is to provide 50 additional bonuses to Eagles and not change the number of bonuses to Rattlers. Option B is to provide 50 fewer bonuses to Rattlers and not change the number of bonuses to Eagles.”

In the single policy condition, participants only read:

“After teams complete the tasks each week, we randomly select participants to receive a bonus for their efforts. In previous weeks, Rattlers have been selected to receive bonuses more often than Eagles. We are considering implementing a new procedure to allocate bonuses more equally across the groups. With this change, we will provide 50 additional bonuses to Eagles and not change the number of bonuses to Rattlers.”

Participants then reported perceived advantaged ingroup resource access [“How does option A (additional Eagles bonuses) affect the Rattlers’ chances of receiving a bonus?”; 7-point Likert scale from −3 = greatly harm to +3 = greatly improve], policy support [“How much do you oppose or support option A (additional Eagles bonuses)?”; 7-point Likert-scale from 1 = strongly oppose to 7 = strongly support], and voting [joint: “Which bonus procedure should be implemented?”; 1 = option A (additional Eagles bonuses), 0 = option B (fewer Rattlers bonuses); single: “Should the new bonus procedure be implemented?”; 1 = option A: yes, 0 = option B: no]. As in study 7, participants reported their identification with the ingroup (α = 0.90) and outgroup (α = 0.82). Participants once again more strongly identified with the Rattlers (*M* = 76.92, SD = 18.13) than the Eagles [*M* = 50.31, SD = 21.15, *t*(495) = −23.44, *P* < 0.001].

### Statistical analyses

#### Studies 1 to 5

We dummy-coded the policy conditions such that the equality-enhancing condition was the intercept (i.e., 0) across all studies. Because all participants saw three vignettes, we fitted a linear mixed model (using the lmerTest R package) in each study, estimated using restricted maximum likelihood (ML). We included policy condition as the fixed effect and participant ID and vignette as random effects in each model. In study 1, we additionally reordered the factor level (using the relevel R package) to set the inequality-enhancing condition as the model intercept and to obtain the comparison between inequality-enhancing and status quo conditions (also see table S1 for Bonferroni post hoc comparisons). The full regression tables for each study are reported in tables S2 to S13. We also conducted analyses to assess order effects (see tables S29 to S34) and to assess whether our effect persisted across each vignette (see figs. S1 to S6).

#### Study 6

We first conducted a one-sample *t* test to determine whether California voters in our sample perceived California Proposition 16 as harmful to their ingroup. We then tested whether perceived advantaged ingroup resource access predicted policy support and voting preference at T1. We fitted a linear regression, estimated using ordinary least squares (OLS) to determine whether perceived advantaged ingroup resource access predicted policy support. We fitted a logistic regression model, estimated using ML, including perceived advantaged group resource access as the predictor variable and voting preference as the dependent variable. We also fitted separate linear regression (for policy support) and logistic regression model (for voting preference) that included ideological beliefs as control variables. All ideological belief measures were mean-centered.

We also examined whether changes in perceived advantaged ingroup resource access predicted changes in policy support and voting preference. We calculated difference scores for perceived ingroup resource access and policy support by subtracting T1 ratings from T2 ratings. Thus, positive scores indicated increases in perceived resource access, zero indicated no change, and negative scores indicated decreases in perceived resource access. We calculated change in vote preference by subtracting T1 from T2, creating three categories: no change (*n* = 635; 84.4%), change from yes to no (*n* = 65; 8.6%), and change from no to yes (*n* = 49; 6.5%). To assess whether changes in perceived advantaged ingroup resource access predicted changes in policy support, we fitted a regression model (estimated from OLS). To assess whether changes in perceived advantaged ingroup resource access predicted changes in voting preference, we fitted a multinomial logistic regression (nnet R package) with no change as the baseline and *P* values calculated using the Wald test. We also fitted a separate regression model and a multinomial logistic regression model including ideological beliefs (all mean-centered) as control variables. The full regression tables for this study are reported in tables S14 to S24. We report the results from a subgroup analysis by participant race in tables S14 to S16, S18 to S20, and S22 to S24.

#### Studies 7 and 8

We conducted an independent-sample *t* test to examine differences between conditions in perceived advantaged ingroup resource access and policy support. We analyzed the effect of policy condition on voting intention with a chi square analysis. We analyzed the effect of policy condition on perceived support with linear regression, estimated using ML. In study 7, we conducted Hayes PROCESS Model 4 using 10,000 bootstrapped samples to examine whether perceived advantaged ingroup resource access mediated the relationship between policy condition and policy support/voting (*79*). The full regression tables for these studies are reported in tables S25 to S28.

## Supplementary Materials

### This PDF file includes:

Tables S1 to S34 Figs. S1 to S6 Policy conditions for Studies 1 to 5, 7, and 8
