# Supplementary material for: If you rise, I fall: Equality is prevented by the misperception that it harms advantaged groups
Source: Sci Adv. 2022 May 6;8(18):eabm2385. doi: 10.1126/sciadv.abm2385 (PMC9075794; doi:10.1126/sciadv.abm2385)

Science Advances
AAAS

Supplementary Materials for

If you rise, I fall: Equality is prevented by the misperception that it harms advantaged groups

N. Derek Brown*, Drew S. Jacoby-Senghor, Isaac Raymundo

*Corresponding author. Email: d_brown@berkeley.edu

Published 6 May 2022, *Sci. Adv.* **8**, eabm2385 (2022)
DOI: 10.1126/sciadv.abm2385

This PDF file includes:

Tables S1 to S34
Figs. S1 to S6
Policy conditions for Studies 1 to 5, 7, and 8

Supplemental Tables

Table S1. *Study 1a post hoc comparisons*

Contrast	Estimate	*SE*	df	*t*	*p*
Equality – Status Quo	-0.754	0.0875	591	-8.61	< .001
Equality - Inequality	-0.976	0.0875	591	-11.16	< .001
Inequality - Status Quo	-0.222	0.0875	591	-2.54	.034

Note. Post hoc comparisons were calculated using the Bonferroni correction.

Table S2. *Study 1a regression results. The effect of policy condition on perceived advantaged ingroup resource access when accounting for ideological beliefs as model covariates.*

	**DV:** Perceived advantaged ingroup resource access											
	**Model 1**						**Model 2**					
Predictors	*Estimates*	*SE*	*df*	*t*	*p*	*95% CI*	*Estimates*	*SE*	*df*	*t*	*p*	*95% CI*
(Intercept | Equality-enhancing condition)	-0.50	0.07	44	-7.06	**<.001**	[-0.64, -0.36]	-0.47	0.07	42	-6.63	**<.001**	[-0.61, -0.33]
Status quo condition	0.75	0.09	591	8.61	**<.001**	[0.58, 0.93]	0.71	0.09	584	8.19	**<.001**	[0.54, 0.88]
Inequality-enhancing condition	0.98	0.09	590	11.16	**<.001**	[0.80, 1.15]	0.92	0.09	584	10.59	**<.001**	[0.75, 1.09]
Explicit prejudice							0.016	0.03	1693	0.53	.60	[-0.04. 0.08]
SDO							-0.094	0.04	590	-2.62	**.009**	[-0.16, -0.02]
SJB							0.10	0.04	584	2.6	**.01**	[0.03, 0.18]
ZSB							0.062	0.03	584	1.91	.057	[-0.001, 0.13]
Political orientation							0.051	0.03	592	1.88	.06	[-0.002, 0.10]
**Random Effects**												
σ^2	0.63						0.63					
$\tau_{00 \text{ResponseID}}$	0.55						0.52					
$\tau_{00 \text{vignette}}$	0.01						0.01					
ICC	0.47						0.45					
$N_{\text{Participant}}$	594						593					
N_{Vignette}	6						6					
Observations	1782						1779					
Marginal R^2	0.128						0.152					
Conditional R^2	0.536						0.537					

Note. Results are from linear mixed regression models. All ideological variables were mean-centered. SDO = social dominance orientation. SJB = system justifying beliefs. ZSB = global zero-sum beliefs. Participants indicated explicit prejudice towards the disadvantaged group in each policy vignette they were presented.

Table S3. *Study 1a moderation results. Interactive effects of policy condition x ideological beliefs on perceived advantaged ingroup resource access.*

	**DV:** Perceived advantaged ingroup resource access					
Predictors	*Estimates*	*SE*	*df*	*t*	*p*	*95% CI*
(Intercept | Equality-enhancing condition)	-0.44	0.07	42.70	-6.15	**<.001**	[-0.58, -0.30]
Status quo condition	0.67	0.09	575	7.82	**<.001**	[0.51, 0.84]
Inequality-enhancing condition	0.90	0.09	574	10.44	**<.001**	[0.73, 1.07]
Status quo condition X Explicit prejudice	-0.08	0.07	1696	-1.13	.26	[-0.23, 0.06]
Inequality-enhancing condition X Explicit prejudice	-0.10	0.08	1684	-1.25	.21	[-0.25, 0.05]
Status quo condition X SDO	0.02	0.09	581	0.20	.84	[-0.15, 0.19]
Inequality-enhancing condition X SDO	0.07	0.09	582	0.80	.43	[-0.10, 0.24]
Status quo condition X SJB	-0.09	0.10	574	-0.86	.39	[-0.28, 0.11]
Inequality-enhancing condition X SJB	0.04	0.10	575	0.40	.69	[-0.15, 0.23]
Status quo condition X ZSB	0.03	0.08	574	0.35	.73	[-0.13, 0.18]
Inequality-enhancing condition X ZSB	0.06	0.08	576	0.73	.47	[-0.10, 0.21]
Status quo condition X Political orientation	-0.18	0.06	579	-2.72	**.007**	[-0.30, -0.05]
Inequality-enhancing condition X Political orientation	-0.11	0.07	585	-1.71	.09	[-0.24, 0.02]
**Random Effects**						
σ^2	0.63					
$\tau_{00 \text{ResponseId}}$	0.5					
$\tau_{00 \text{vignette}}$	0.01					
ICC	0.45					
$N_{ResponseId}$	593					
N_{vignette}	6					
Observations	1779					
Marginal R^2	0.17					
Conditional R^2	0.54					

Note. Results are from linear mixed regression models. All ideological variables were mean-centered and entered as simultaneous moderators. SDO = social dominance orientation. SJB = system justifying beliefs. ZSB = global zero-sum beliefs. Participants indicated explicit prejudice towards the disadvantaged group in each policy vignette they were presented.

Table S4. *Study 1b regression results. The effect of policy condition on perceived advantaged ingroup resource access when accounting for ideological beliefs as model covariates.*

	**DV:** Perceived advantaged ingroup resource access											
	**Model 1**						**Model 2**					
Predictors	*Estimates*	*SE*	*df*	*t*	*p*	*95% CI*	*Estimates*	*SE*	*df*	*t*	*p*	*95% CI*
(Intercept | Equality-enhancing condition)	-0.35	0.069	397	-5.07	**<.001**	[-0.49, -0.21]	-0.34	0.07	391	-5.01	**<.001**	[-0.47, -0.21]
Inequality-enhancing condition	1.07	0.098	397	10.97	**<.001**	[0.88, 1.26]	1.05	0.10	393	10.87	**<.001**	[0.86, 1.24]
Symbolic threat							0.03	0.03	1087	0.78	.43	[-0.039, 0.09]
Status threat							-0.07	0.03	903	-2.85	**.004**	[-0.12, -0.022]
Explicit prejudice							0.09	0.04	1111	2.12	**.034**	[0.01, 0.18]
SDO							-0.04	0.05	419	-0.74	.46	[-0.13, 0.061]
SJB							0.01	0.05	393	0.22	.83	[-0.09, 0.11]
ZSB							0.07	0.04	392	1.64	.10	[-0.014, 0.16]
Political orientation							0.04	0.04	394	0.95	.34	[-0.04, 0.11]
**Random Effects**												
σ^2	0.66						0.65					
$\tau00_{\text{ResponseId}}$	0.73						0.7					
$\tau00_{\text{vignette}}$	<.001						<.001					
$N_{\text{ResponseId}}$	399						398					
N_{vignette}	6						6					
Observations	1197						1184					
Marginal R^2	0.302						0.342					

Note. Results are from linear mixed regression models. All ideological variables were mean-centered. SDO = social dominance orientation. SJB = system justifying beliefs. ZSB = global zero-sum beliefs. Participants indicated symbolic threat, status threat, and explicit prejudice towards the disadvantaged group in each policy vignette they were presented.

Table S5. *Study 1b moderation results. Interactive effects of policy condition x ideological beliefs on perceived advantaged ingroup resource access.*

	**DV:** Perceived advantaged ingroup resource access					
Predictors	*Estimates*	*SE*	*df*	*t*	*p*	*95% CI*
(Intercept | Equality-enhancing condition)	-0.34	0.07	386	-5.08	**<.001**	[-0.47, -0.21]
Inequality-enhancing condition	1.04	0.09	388	11.09	**<.001**	[0.86, 1.23]
Policy condition X Symbolic threat	0.05	0.07	1098	0.76	.448	[-0.08, 0.18]
Policy condition X Status threat	0.07	0.05	877	1.34	.179	[-0.03, 0.16]
Policy condition X Explicit prejudice	-0.03	0.09	1108	-0.32	.751	[-0.20, 0.14]
Policy condition X SDO	0.19	0.10	419	1.95	.051	[-0.002, 0.38]
Policy condition X SJB	-0.08	0.10	389	-0.83	.409	[-0.29, 0.12]
Policy condition X ZSB	0.30	0.09	387	3.52	**<.001**	[0.13, 0.47]
Policy condition X Political orientation	-0.14	0.08	397	-1.81	.071	[-0.29, 0.01]
**Random Effects**						
σ^2	0.65					
$\tau_{00\ \text{ResponseId}}$	0.65					
$\tau_{00\ \text{vignette}}$	<.001					
$N_{\text{ResponseId}}$	398					
N_{vignette}	6					
Observations	1184					
Marginal R^2	0.38					

Note. Results are from linear mixed regression models. All ideological variables were mean-centered and entered as simultaneous moderators. SDO = social dominance orientation. SJB = system justifying beliefs. ZSB = global zero-sum beliefs. Participants indicated symbolic threat, status threat, and explicit prejudice towards the disadvantaged group in each policy vignette they were presented.

Table S6. *Study 2 regression results. The effect of policy condition on perceived advantaged ingroup resource access when accounting for ideological beliefs as model covariates.*

	**DV:** Perceived advantaged ingroup resource access											
	**Model 1**						**Model 2**					
Predictors	*Estimates*	*SE*	*df*	*t*	*p*	*95% CI*	*Estimates*	*SE*	*df*	*t*	*p*	*95% CI*
(Intercept | Intergroup condition)	-0.32	0.09	9.82	-3.69	**.004**	[-0.49, -0.14]	-0.32	0.09	8.97	-3.73	**.005**	[-0.49, -0.14]
Ingroup condition	0.19	0.10	385	2.00	**.046**	[0.004, 0.38]	0.18	0.09	379	1.91	.058	[-0.004, 0.36]
Explicit prejudice							-0.03	0.04	1134	-0.72	.47	[-0.10, 0.04]
SDO							-0.10	0.04	410	-2.42	**.016**	[-0.18, -0.02]
SJB							0.19	0.04	380	4.47	**<.001**	[0.11, 0.28]
ZSB							0.07	0.04	379	1.77	.08	[-0.01, 0.14]
Political orientation							0.09	0.03	379	2.76	**.006**	[0.03, 0.15]
**Random Effects**												
σ^2	0.56						0.57					
$\tau_{00\ \text{Participant}}$	0.70						0.63					
$\tau_{00\ \text{Vignette}}$	0.01						0.01					
ICC	0.56						0.53					
$N_{\text{Participant}}$	387						387					
N_{Vignette}	3						3					
Observations	1161						1155					
Marginal R^2	0.007						0.061					
Conditional R^2	0.56						0.56					

Note. Results are from linear mixed regression models. All ideological variables were mean-centered. SDO = social dominance orientation. SJB = system justifying beliefs. ZSB = global zero-sum beliefs. Participants indicated explicit prejudice towards the disadvantaged group in each policy vignette they were presented.

Table S7. *Study 2 moderation results. Interactive effects of policy condition x ideological beliefs on perceived advantaged ingroup resource access.*

	**DV:** Perceived advantaged ingroup resource access					
Predictors	*Estimate*	*SE*	*df*	*t*	*p*	*95% CI*
(Intercept | Intergroup condition)	-0.33	0.09	8.78	-3.84	**<.001**	[-0.49, -0.16]
Ingroup condition	0.18	0.09	374	1.95	.052	[0.001, 0.36]
Policy condition X Explicit prejudice	-0.12	0.07	1128	-1.64	.10	[-0.26, 0.02]
Policy condition X SDO	0.08	0.08	407	1.00	.32	[-0.08, 0.24]
Policy condition X SJB	-0.04	0.09	376	-0.50	.62	[-0.21, 0.13]
Policy condition X ZSB	0.17	0.08	375	2.17	**.031**	[0.02, 0.32]
Policy condition X Political orientation	-0.07	0.06	375	-1.15	.25	[-0.20, 0.05]
**Random Effects**						
σ^2	0.57					
$\tau_{00 ResponseId}$	0.62					
$\tau_{00 vignette}$	0.01					
ICC	0.52					
$N_{ResponseId}$	385					
$N_{vignette}$	3					
Observations	1155					
Marginal R^2	0.08					
Conditional R^2	0.56					

Note. Results are from linear mixed regression models. All ideological variables were mean-centered and entered as simultaneous moderators. SDO = social dominance orientation. SJB = system justifying beliefs. ZSB = global zero-sum beliefs. Participants indicated explicit prejudice towards the disadvantaged group in each policy vignette they were presented.

Table S8. *Study 3 regression results. The effect of policy condition on perceived advantaged ingroup resource access when accounting for ideological beliefs as model covariates.*

	**DV:** Perceived advantaged ingroup resource access											
	**Model 1**						**Model 2**					
Predictors	*Estimates*	*SE*	*df*	*t*	*p*	*95% CI*	*Estimates*	*SE*	*df*	*t*	*p*	*95% CI*
(Intercept | Equality-enhancing condition)	-0.33	0.08	56.79	-4.39	**<.001**	[-0.48, -0.18]	-0.31	0.07	58.49	-4.18	**<.001**	[-0.45, -0.16]
Inequality-enhancing condition	0.35	0.10	391	3.61	**<.001**	[0.16, 0.54]	0.31	0.10	386	3.17	**.002**	[0.12, 0.50]
Explicit prejudice							-0.01	0.04	1105	-0.11	.91	[-0.09, 0.08]
SDO							-0.08	0.05	389	-1.58	.12	[-0.17, 0.02]
SJB							0.01	0.05	388	0.22	.83	[-0.09, 0.11]
ZSB							0.06	0.04	386	1.39	.17	[-0.02, 0.14]
Political orientation							0.06	0.04	386	1.69	.09	[-0.01, 0.13]
**Random Effects**												
σ^2	0.7						0.7					
τ_{00} Participant	0.70						0.66					
τ00 Vignette	0.01						0.004					
ICC	0.5						0.49					
N Participant	393						393					
N Vignette	6						6					
Observations	1178						1178					
Marginal R^2	0.02						0.06					
Conditional R^2	0.51						0.52					

Note. Results are from linear mixed regression models. All ideological variables were mean-centered. SDO = social dominance orientation. SJB = system justifying beliefs. ZSB = global zero-sum beliefs. Participants indicated explicit prejudice towards the disadvantaged group in each policy vignette they were presented.

Table S9. *Study 3 moderation results. Interaction between effects of policy condition x ideological beliefs on perceived advantaged ingroup resource access.*

	**DV:** Perceived advantaged ingroup resource access					
Predictors	*Estimates*	*SE*	*df*	*t*	*p*	*95% CI*
(Intercept | Equality-enhancing condition)	-0.30	0.07	59.50	-4.12	**<.001**	[-0.45, -0.16]
Inequality-enhancing condition	0.30	0.10	382	3.14	**.002**	[0.12, 0.49]
Policy condition X Explicit prejudice	-0.14	0.09	1103	-1.62	.10	[-0.31, 0.028]
Policy condition X SDO	0.17	0.10	386	1.72	.09	[-0.02, 0.36]
Policy condition X SJB	-0.06	0.11	385	-0.61	.54	[-0.27, 0.14]
Policy condition X ZSB	0.002	0.08	382	0.03	.98	[-0.16, 0.17]
Policy condition X Political orientation	-0.01	0.07	382	-0.07	.95	[-0.15, 0.14]
**Random Effects**						
σ^2	0.7					
$\tau_{00\text{ResponseId}}$	0.66					
$\tau_{00\text{vignette}}$	<.001					
ICC	0.49					
$N_{\text{ResponseId}}$	393					
N_{vignette}	6					
Observations	1178					
Marginal R^2	0.07					
Conditional R^2	0.52					

Notes. Results are from linear mixed regression models. All ideological variables were mean-centered and entered as simultaneous moderators. SDO = social dominance orientation. SJB = system justifying beliefs. ZSB = global zero-sum beliefs. Participants indicated explicit prejudice towards the disadvantaged group in each policy vignette they were presented.

Table S10. *Study 4 regression results. The effect of policy condition on perceived advantaged ingroup resource access when accounting for ideological beliefs as model covariates.*

	**DV:** Perceived advantaged ingroup resource access											
	**Model 1**						**Model 2**					
Predictors	*Estimates*	*SE*	*df*	*t*	*p*	*95% CI*	*Estimates*	*SE*	*df*	*t*	*p*	*95% CI*
(Intercept | Equality-enhancing, Limited resource condition)	-0.30	0.10	157	-3.12	**.002**	[-0.48, -0.11]	-0.30	0.09	156	-3.14	**.002**	[-0.48, -0.11]
Inequality-enhancing condition	0.82	0.13	389	6.27	**<.001**	[0.56, 1.08]	0.82	0.13	384	6.30	**<.001**	[0.57, 1.07]
Unlimited resource condition	-0.05	0.13	389	-0.35	.73	[-0.30, 0.21]	-0.05	0.13	384	-0.40	.69	[-0.30, 0.20]
Policy X Resource limit	0.29	0.18	389	1.57	.12	[-0.07, 0.65]	0.30	0.18	384	1.64	.10	[-0.05, 0.65]
Explicit prejudice							0.05	0.04	1085	1.33	.18	[-0.03, 0.13]
SDO							-0.05	0.05	404	-1.13	.26	[-0.15, 0.04]
SJB							0.02	0.05	385	0.45	.65	[-0.07, 0.12]
ZSB							-0.003	0.04	385	-0.07	.94	[-0.08, 0.08]
Political orientation							0.07	0.04	385	1.93	.055	[-0.001, 0.14]
**Random Effects**												
σ^2	0.69						0.69					
$\tau_{00\ \mathrm{ResponseId}}$	0.60						0.58					
$\tau_{00\ \mathrm{Vignette}}$	0.004						0.004					
ICC	0.47						0.46					
$N_{\mathrm{ResponseId}}$	393						393					
N_{Vignette}	6						6					
Observations	1179						1179					
Marginal R^2	0.16						0.18					
Conditional R^2	0.55						0.56					

Note. Results are from linear mixed regression models. All ideological variables were mean-centered. SJB = system justifying beliefs. ZSB = global zero-sum beliefs. Participants indicated explicit prejudice towards the disadvantaged group in each policy vignette they were presented.

Table S11. *Study 4 moderation results. Interaction between effects of policy condition x ideological beliefs on perceived advantaged ingroup resource access.*

	**DV:** Perceived advantaged ingroup resource access					
Predictors	*Estimates*	*SE*	*df*	*t*	*p*	*95% CI*
(Intercept | Equality-enhancing, Limited condition)	-0.33	0.09	1560	-3.45	**<.001**	[-0.51, -0.15]
Inequality-enhancing condition	0.88	0.13	372	6.70	**<.001**	[0.63, 1.13]
Unlimited resource condition	-0.02	0.13	372	-0.18	.86	[-0.27, 0.22]
Policy X Resource	0.23	0.18	372	1.24	.21	[-0.12, 0.58]
Policy X Explicit prejudice	0.02	0.11	1076	0.18	.86	[-0.20, 0.24]
Policy X SDO	-0.34	0.13	406	-2.58	**.010**	[-0.59, -0.09]
Policy X SJB	-0.04	0.14	372	-0.30	.77	[-0.32, 0.23]
Policy X ZSB	-0.21	0.12	374	-1.74	.08	[-0.44, 0.02]
Policy X Political orientation	-0.18	0.10	373	-1.78	.08	[-0.37, 0.01]
Resource X Explicit prejudice	0.14	0.12	1078	1.15	.25	[-0.10, 0.37]
Resource X SDO	-0.28	0.14	403	-1.98	**.048**	[-0.54, -0.01]
Resource X SJB	0.09	0.14	375	0.68	.50	[-0.17, 0.36]
Resource X ZSB	-0.01	0.11	373	-0.12	.90	[-0.23, 0.20]
Resource X Political Orientation	-0.16	0.10	373	-1.53	.13	[-0.36, 0.04]
Policy X Resource X Explicit Prejudice	-0.12	0.16	1075	-0.75	.45	[-0.43, 0.19]
Policy X Resource X SDO	0.32	0.19	393	1.67	.10	[-0.05, 0.70]
Policy X Resource X SJB	-0.03	0.20	374	-0.13	.90	[-0.40, 0.35]
Policy X Resource X ZSB	0.11	0.17	373	0.64	.52	[-0.21, 0.43]
Policy X Resource X Political orientation	0.11	0.14	372	0.76	.45	[-0.16, 0.38]
**Random Effects**						
σ^2	0.68					
$\tau_{00 ResponseId}$	0.57					
$\tau_{00 vignette}$	<.001					
ICC	0.46					
$N_{ResponseId}$	393					
$N_{vignette}$	6					
Observations	1179					
Marginal R^2	0.20					
Conditional R^2	0.56					

Notes. Results are from linear mixed regression models. All ideological variables were mean-centered and entered as simultaneous moderators. SDO = social dominance orientation. SJB = system justifying beliefs. ZSB = global zero-sum beliefs. Participants indicated explicit prejudice towards the disadvantaged group in each policy vignette they were presented.

Table S12. *Study 5 regression results. The effect of policy condition on perceived advantaged ingroup resource access when accounting for ideological beliefs as model covariates.*

	**DV:** Perceived advantaged ingroup resource access											
	**Model 1**						**Model 2**					
Predictors	*Estimates*	*SE*	*df*	*t*	*p*	*95% CI*	*Estimates*	*SE*	*df*	*t*	*p*	*95% CI*
(Intercept | Limited access condition)	-1.06	0.06	82.01	-17.23	**<.001**	[-1.18, -0.94]	-1.03	0.06	101.63	-17.47	**<.001**	[-1.14, -0.91]
Unlimited access condition	0.93	0.08	396	11.12	**<.001**	[0.77, 1.10]	0.87	0.08	389.06	10.48	**<.001**	[0.71, 1.04]
Explicit prejudice							0.01	0.04	1135.63	0.39	.69	[-0.06, 0.08]
SDO							0.02	0.04	404.72	0.43	.67	[-0.07, 0.10]
SJB							-0.04	0.04	390.97	-0.91	.37	[-0.12, 0.04]
ZSB							-0.01	0.04	389.77	-0.34	.74	[-0.08, 0.06]
Political orientation							0.12	0.03	389.39	3.80	**<.001**	[0.06, 0.18]
**Random Effects**												
σ^2	0.57						0.57					
$\tau_{00\ Participant}$	0.51						0.47					
$\tau_{00\ Vignette}$	0.001						<0.001					
ICC	0.47						0.45					
$N_{Participant}$	399						396					
$N_{Vignette}$	6						6					
Observations	1197						1188					
Marginal R^2	0.17						0.20					
Conditional R^2	0.56						0.57					

Note. Results are from linear mixed regression models. All ideological variables were mean-centered. SJB = system justifying beliefs. ZSB = global zero-sum beliefs. Participants indicated explicit prejudice towards the disadvantaged group in each policy vignette they were presented.

Table S13. *Study 5 moderation results. Interaction between effects of policy condition x ideological beliefs on perceived advantaged ingroup resource access.*

	**DV:** Perceived advantaged ingroup resource access				
Predictors	*Estimates*	*SE*	*t*	*p*	*95% CI*
(Intercept | Limited access condition)	-1.01	0.06	-17.10	**<.001**	[-1.13, -0.90]
Unlimited access condition	0.88	0.08	10.56	**<.001**	[0.71, 1.04]
Policy condition X Explicit prejudice	0.09	0.07	1.29	.20	[-0.05, 0.23]
Policy condition X SDO	0.05	0.09	0.51	.61	[-0.13, 0.22]
Policy condition X SJB	0.02	0.09	0.21	.84	[-0.15, 0.18]
Policy condition X ZSB	-0.07	0.07	-1.03	.31	[-0.21, 0.07]
Policy condition X Political orientation	-0.07	0.06	-1.10	.27	[-0.19, 0.05]
**Random Effects**					
σ^2	0.57				
$\tau_{00 \text{ResponseId}}$	0.47				
$\tau_{00 \text{vignette}}$	<.001				
ICC	0.45				
$N_{\text{ResponseId}}$	396				
N_{vignette}	6				
Observations	1188				
Marginal R^2	0.21				
Conditional R^2	0.57				

Note. Results are from linear mixed regression models. All ideological variables were mean-centered and entered as simultaneous moderator. SJB = system justifying beliefs. ZSB = global zero-sum beliefs. Participants indicated explicit prejudice towards the disadvantaged group in each policy vignette they were presented.

Table S14. *Study 6 regression results, White participant sub-analysis. The effect of changes in perceived advantaged ingroup resource access on changes in policy support when accounting for ideological beliefs.*

	**DV: Policy support**						**DV: Vote preference**					
Predictors	*Estimates*	*SE*	*df*	*t*	*p*	*95% CI*	*Estimates*	*SE*	*z*	*p*	*OR*	*95% CI*
(Intercept)	0.10	0.08	404	1.29	.20	[-0.05, 0.26]	0.40	0.13	3.10	**.002**	1.49	[0.15, 0.66]
Perceived ingroup resource access	0.57	0.06	404	9.75	**<.001**	[0.45, 0.68]	0.75	0.11	6.66	**<.001**	2.11	[0.54, 0.98]
Explicit prejudice	0.27	0.06	404	4.50	**<.001**	[0.15, 0.39]	0.41	0.10	4.04	**<.001**	1.50	[0.21, 0.61]
SDO	-0.29	0.08	404	-3.46	**.001**	[-0.46, -0.13]	-0.20	0.14	-1.40	.162	0.82	[-0.47, 0.08]
SJB	0.18	0.08	404	2.33	**.02**	[0.03, 0.34]	0.31	0.14	2.27	**.023**	1.36	[0.05, 0.58]
ZSB	0.13	0.07	404	1.90	.058	[-0.004, 0.27]	0.22	0.11	1.99	**.046**	1.25	[0.005, 0.45]
Political orientation	0.25	0.05	404	4.64	**<.001**	[0.14, 0.35]	0.37	0.09	4.11	**<.001**	1.45	[0.20, 0.56]
N	411						411					
R^2	0.412						—					
R^2 adjusted	0.403						—					
R^2 Tjur	—						0.342					

Note. White participant sub-sample (n = 411). The effect of perceived advantaged ingroup resource access on policy support remained significant controlling for ideological beliefs: explicit prejudice, social dominance orientation (SDO), system justifying beliefs (SJB), global zero-sum beliefs (ZSB), and political orientation. Similarly, the effect of perceived advantaged ingroup resource access on voting preferences remained significant controlling for ideological beliefs. Vote preference was coded such that 0=Against, 1=In favor. OR = Odds Ratio.

.

Table S15. *Study 6 regression results, East Asian participant sub-analysis. The effect of changes in perceived advantaged ingroup resource access on changes in policy support when accounting for ideological beliefs.*

	**DV: Policy support**						**DV: Vote preference**					
Predictors	*Estimates*	*SE*	*df*	*t*	*p*	*95% CI*	*Estimates*	*SE*	*z*	*p*	*OR*	*95% CI*
(Intercept)	0.10	0.10	309	1.03	.31	[-0.09, 0.29]	0.44	0.15	2.91	**.004**	1.55	[0.15, 0.74]
Perceived ingroup resource access	0.41	0.07	309	5.83	**<.001**	[0.27, 0.55]	0.52	0.12	4.36	**<.001**	1.69	[0.30, 0.77]
Explicit prejudice	0.26	0.07	309	3.88	**<.001**	[0.13, 0.39]	0.33	0.11	3.06	**.002**	1.39	[0.12, 0.54]
SDO	-0.28	0.10	309	-2.73	**.007**	[-0.49, -0.08]	-0.33	0.17	-1.98	**.048**	0.72	[-0.67, -0.01]
SJB	-0.09	0.11	309	-0.76	.45	[-0.31, 0.14]	-0.16	0.18	-0.87	.39	0.86	[-0.51, 0.20]
ZSB	0.22	0.08	309	2.78	**.006**	[0.07, 0.38]	0.32	0.13	2.44	**.015**	1.38	[0.07, 0.59]
Political orientation	0.11	0.08	309	1.28	.20	[-0.06, 0.27]	0.09	0.13	0.74	.46	1.10	[-0.15, 0.34]
N	316						316					
R^2	0.338						—					
R^2 adjusted	0.325						—					
R^2 Tjur	—						0.246					

Note. East Asian participant sub-sample (*n* = 316). The effect of perceived advantaged ingroup resource access on policy support remained significant controlling for ideological beliefs: explicit prejudice, social dominance orientation (SDO), system justifying beliefs (SJB), global zero-sum beliefs (ZSB), and political orientation. Similarly, the effect of perceived advantaged ingroup resource access on voting preferences remained significant controlling for ideological beliefs. Vote preference was coded such that 0=Against, 1=In favor. OR = Odds Ratio.

Table S16. *Study 6 regression results, South Asian participant sub-analysis. The effect of changes in perceived advantaged ingroup resource access on changes in policy support when accounting for ideological beliefs.*

	DV: Policy support						DV: Vote preference					
Predictors	*Estimates*	*SE*	*df*	*t*	*p*	*95% CI*	*Estimates*	*SE*	*z*	*p*	*OR*	*95% CI*
(Intercept)	-0.04	0.18	86	-0.25	.81	[-0.39, 0.31]	0.001	0.27	0.004	1.00	1.00	[-0.53, 0.52]
Perceived ingroup resource access	0.37	0.13	86	2.89	**.005**	[0.12, 0.63]	0.57	0.22	2.61	**0.009**	1.77	[0.17, 1.04]
Explicit prejudice	0.24	0.15	86	1.57	.12	[-0.06, 0.54]	0.34	0.22	1.52	0.13	1.40	[-0.09, 0.81]
SDO	0.16	0.19	86	0.82	.41	[-0.22, 0.53]	0.26	0.29	0.91	0.37	1.30	[-0.29, 0.87]
SJB	-0.12	0.18	86	-0.66	.51	[-0.49, 0.24]	-0.36	0.29	-1.27	0.21	0.70	[-0.95, 0.19]
ZSB	0.20	0.17	86	1.18	.24	[-0.14, 0.53]	0.15	0.25	0.6	0.55	1.16	[-0.35, 0.65]
Political orientation	0.25	0.18	86	1.39	.17	[-0.11, 0.60]	0.17	0.26	0.66	0.51	1.19	[-0.35, 0.70]
N	93						93					
R^2	0.257						—					
R^2 adjusted	0.205						—					
R^2 Tjur	—						0.206					

Note. South Asian participant sub-sample (n = 93). The effect of perceived advantaged ingroup resource access on policy support remained significant controlling for ideological beliefs: explicit prejudice, social dominance orientation (SDO), system justifying beliefs (SJB), global zero-sum beliefs (ZSB), and political orientation. Similarly, the effect of perceived advantaged ingroup resource access on voting preferences remained significant controlling for ideological beliefs. Vote preference was coded such that 0=Against, 1=In favor. OR = Odds Ratio.

Table S17. *Study 6 regression results. The effect of changes in perceived advantaged ingroup resource access on changes in policy support when accounting for ideological beliefs.*

	**Dependent variable: Δ** Policy support											
	**Model 1**						**Model 2**					
Predictors	*Estimates*	*SE*	*df*	*t*	*P*	*95% CI*	*Estimates*	*SE*	*df*	*t*	*p*	*95% CI*
(Intercept)	< 0.001	0.05	643	<0.001	1.00	[-0.10, 0.10]	-0.01	0.05	638	-0.14	.89	[-0.11, 0.09]
**Δ** Perceived advantaged ingroup resource access	0.30	0.04	643	6.86	**<.001**	[0.21, 0.38]	0.29	0.04	638	6.70	**<.001**	[0.21, 0.38]
Explicit prejudice							-0.07	0.04	638	-1.78	.08	[-0.15, 0.01]
SDO							-0.04	0.06	638	-0.69	.49	[-0.15, 0.07]
SJB							-0.06	0.06	638	-1.12	.26	[-0.18, 0.05]
ZSB							-0.05	0.05	638	-1.12	.26	[-0.14, 0.04]
Political orientation							0.06	0.04	638	1.34	.18	[-0.03, 0.14]
N	645						645					
R^2	0.068						0.081					
R^2 adjusted	0.067						0.072					

Note. Results are from linear regression models. **Δ** Perceived advantaged ingroup resource access denotes change in perceived advantaged ingroup resource access (T2 minus T1). All ideological variables were mean-centered. SDO = social dominance orientation; SJB = system justifying beliefs; ZSB = global zero-sum beliefs.

Table S18. *Study 6 regression results, White participant sub-analysis. The effect of changes in perceived advantaged ingroup resource access on changes in policy support when accounting for ideological beliefs.*

	**Dependent variable: Δ Policy support**											
	**Model 1**						**Model 2**					
Predictors	*Estimates*	*SE*	*df*	*t*	*p*	*95% CI*	*Estimates*	*SE*	*df*	*t*	*p*	*95% CI*
(Intercept)	0.02	0.07	320	0.23	.82	[-0.12, 0.15]	0.01	0.07	315	0.10	.92	[-0.14, 0.15]
Δ Perceived advantaged ingroup resource access	0.36	0.06	320	6.05	**<.001**	[0.24, 0.47]	0.35	0.06	315	5.76	**<.001**	[0.23, 0.46]
Explicit prejudice							0.001	0.06	315	0.03	.98	[-0.11, 0.11]
SDO							-0.07	0.08	315	-0.87	.39	[-0.22, 0.084]
SJB							-0.11	0.07	315	-1.60	.11	[-0.25, 0.026]
ZSB							-0.002	0.06	315	-0.03	.98	[-0.12, 0.12]
Political orientation							-0.05	0.05	315	-0.94	.35	[-0.15, 0.054]
N	322						322					
R^2	0.103						0.117					
R^2 adjusted	0.10						0.10					

Note. Results are from linear regression models. **Δ** Perceived advantaged ingroup resource access denotes change in perceived advantaged ingroup resource access (T2 minus T1). All ideological variables were mean-centered. SDO = social dominance orientation; SJB = system justifying beliefs; ZSB = global zero-sum beliefs.

Table S19. *Study 6 regression results, East Asian participant sub-analysis. The effect of changes in perceived advantaged ingroup resource access on changes in policy support when accounting for ideological beliefs.*

	**Dependent variable: Δ** Policy support											
	**Model 1**						**Model 2**					
Predictors	*Estimates*	*SE*	*df*	*t*	*p*	*95% CI*	*Estimates*	*SE*	*df*	*t*	*p*	*95% CI*
(Intercept)	-0.03	0.08	248	-0.36	.72	[-0.20, 0.13]	-0.08	0.09	243	-0.88	.38	[-0.25, 0.10]
**Δ** Perceived advantaged ingroup resource access	0.22	0.07	248	2.95	**.004**	[0.07, 0.36]	0.24	0.07	243	3.23	**.001**	[0.09, 0.38]
Explicit prejudice							-0.13	0.07	243	-2.01	**.045**	[-0.26, -0.003]
SDO							0.08	0.10	243	0.78	.43	[-0.12,0.27]
SJB							-0.13	0.11	243	-1.15	.25	[-0.34, 0.09]
ZSB							-0.14	0.08	243	-1.84	.067	[-0.30, 0.01]
Political orientation							0.16	0.08	243	2.03	**.043**	[0.01, 0.32]
N	250						250					
R^2	0.034						0.079					
R^2 adjusted	0.03						0.056					

Note. Results are from linear regression models. **Δ** Perceived advantaged ingroup resource access denotes change in perceived advantaged ingroup resource access (T2 minus T1). All ideological variables were mean-centered. SDO = social dominance orientation; SJB = system justifying beliefs; ZSB = global zero-sum beliefs.

Table S20. *Study 6 regression results, South Asian participant sub-analysis. The effect of changes in perceived advantaged ingroup resource access on changes in policy support when accounting for ideological beliefs.*

	**Dependent variable: Δ** Policy support											
	**Model 1**						**Model 2**					
Predictors	*Estimates*	*SE*	*df*	*t*	*p*	*95% CI*	*Estimates*	*SE*	*df*	*t*	*p*	*95% CI*
(Intercept)	0.01	0.19	71	0.03	.97	[-0.38, 0.39]	-0.02	0.21	66	-0.11	.91	[-0.44, 0.39]
**Δ** Perceived advantaged ingroup resource access	0.30	0.13	71	2.25	**.03**	[0.03, 0.57]	0.28	0.13	66	2.12	**.04**	[0.02, 0.55]
Explicit prejudice	—						-0.25	0.18	66	-1.41	.17	[-0.60, 0.11]
SDO							-0.34	0.25	66	-1.38	.17	[-0.84, 0.15]
SJB							0.37	0.21	66	1.74	.09	[-0.05, 0.77]
ZSB							-0.05	0.20	66	-0.26	.79	[-0.46, 0.35]
Political orientation	–						0.42	0.20	66	2.13	**.04**	[0.03, 0.82]
N	73						73					
R^2	0.066						0.165					
R^2 adjusted	0.053						0.089					

Note. Results are from linear regression models. **Δ** Perceived advantaged ingroup resource access denotes change in perceived advantaged ingroup resource access (T2 minus T1). All ideological variables were mean-centered. SDO = social dominance orientation; SJB = system justifying beliefs; ZSB = global zero-sum beliefs.

Table S21. *Study 6 regression results. The effect of changes in perceived advantaged ingroup resource access on changes in voting preference when accounting for ideological beliefs.*

	Dependent variable: **Δ** Vote preference													
	**Model 1**							**Model 2**						
Predictors	*Estimates*	*SE*	*z*	*p*	*OR*	*95% CI*	*Response*	*Estimates*	*SE*	*z*	*p*	*OR*	*95% CI*	*Response*
(Intercept)	-2.32	0.15	-15.81	**<.001**	0.10	[0.07, 0.13]	Y-N	-2.43	0.16	-15.15	**<.001**	0.09	[0.06, 0.12]	Y-N
	-2.65	0.17	-15.67	**<.001**	0.07	[0.05, 0.10]	N-Y	-2.69	0.18	-15.17	**<.001**	0.07	[0.05, 0.10]	N-Y
**Δ** Perceived advantaged ingroup resource access	-0.39	0.10	-3.89	**<.001**	0.68	[0.56, 0.82]	Y-N	-0.39	0.10	-3.69	**<.001**	0.68	[0.55, 0.83]	Y-N
	0.19	0.15	1.28	.20	1.21	[0.90, 1.62]	N-Y	0.19	0.15	1.23	.22	1.21	[0.89, 1.64]	N-Y
Explicit prejudice								-0.25	0.10	-2.55	**.011**	0.78	[0.64, 0.94]	Y-N
								0.03	0.14	0.19	.85	1.03	[0.78, 1.34]	N-Y
SDO								0.08	0.15	0.51	.61	1.08	[0.80, 1.46]	Y-N
								0.19	0.19	0.99	.33	1.21	[0.83, 1.75]	N-Y
SJB								0.02	0.15	0.16	.87	1.03	[0.76, 1.39]	Y-N
								-0.10	0.18	-0.57	.57	0.90	[0.63, 1.29]	N-Y
ZSB								0.36	0.13	2.73	**.006**	1.43	[1.11, 1.85]	Y-N
								0.13	0.15	0.88	.38	1.14	[0.85, 1.54]	N-Y
Political orientation								-0.04	0.11	-0.38	.71	0.96	[0.77, 1.20]	Y-N
								0.13	0.14	0.90	.37	1.14	[0.86, 1.51]	N-Y
N	645							645						
df	641							631						
R^2 Nagelkerke	0.039							0.085						
AIC	677.78							677.58						

Note. Results are from multinomial logistic regression models. **Δ** Perceived advantaged ingroup resource access denotes change in perceived advantaged ingroup resource access (T2 – T1). All ideological variables were mean-centered. SDO = social dominance orientation; SJB = system justifying beliefs; ZSB = global zero-sum beliefs. Y-N indicates a change from a "Yes" to a "No" vote. N-Y indicates a change from a "No" to a "Yes" vote. OR = Odds Ratio.

Table S22. *Study 6 regression results, White participant sub-analysis. The effect of changes in perceived advantaged ingroup resource access on changes in voting preference when accounting for ideological beliefs.*

	Dependent variable: **Δ** Vote preference													
	**Model 1**							**Model 2**						
Predictors	Estimates	*SE*	*OR*	*95% CI*	*z*	*p*	*Response*	*Estimates*	*SE*	*OR*	*95% CI*	*z*	*p*	*Response*
(Intercept)	-2.35	0.21	0.10	[0.06, 0.15]	-11.14	**< .001**	Y-N	-2.38	0.23	0.09	[0.06, 0.14]	-10.49	**< .001**	Y-N
	-2.86	0.26	0.06	[0.03, 0.10]	-10.94	**< .001**	N-Y	-3.00	0.32	0.05	[0.03, 0.09]	-9.34	**< .001**	N-Y
**Δ** Perceived advantaged ingroup outcome	-0.52	0.14	0.60	[0.45, 0.79]	-3.57	**< .001**	Y-N	-0.50	0.15	0.61	[0.45, 0.82]	-3.27	**.001**	Y-N
	0.06	0.24	1.06	[0.66, 1.69]	0.24	.81	N-Y	0.06	0.25	1.06	[0.65, 1.72]	0.24	.81	N-Y
Explicit prejudice								-0.38	0.14	0.69	[0.52, 0.91]	-2.66	**.008**	Y-N
								0.05	0.24	1.05	[0.66, 1.69]	0.22	.83	N-Y
SDO								0.10	0.22	1.11	[0.72, 1.71]	0.46	.65	Y-N
								-0.31	0.35	0.73	[0.37, 1.47]	-0.88	.38	N-Y
SJB								-0.04	0.21	0.96	[0.64, 1.45]	-0.18	.86	Y-N
								-0.07	0.27	0.93	[0.55, 1.58]	-0.27	.79	N-Y
ZSB								0.36	0.18	1.44	[1.01, 2.06]	2.00	**.047**	Y-N
								0.33	0.23	1.39	[0.88, 2.18]	1.41	.16	N-Y
Political orientation								0.04	0.14	1.04	[0.78, 1.38]	0.27	.78	Y-N
								-0.12	0.20	0.89	[0.60, 1.32]	-0.59	.55	N-Y
N	322							322						
df	318							308						
R^2 Nagelkerke	0.062							0.14						
AIC	318.7							321.71						

Note. Results are from multinomial logistic regression models. **Δ** Perceived advantaged ingroup resource access denotes change in perceived advantaged ingroup resource access (T2 – T1). All ideological variables were mean-centered. SDO = social dominance orientation; SJB = system justifying beliefs; ZSB = global zero-sum beliefs. Y-N indicates a change from a "Yes" to a "No" vote. N-Y indicates a change from a "No" to a "Yes" vote. OR = Odds Ratio.

Table S23. *Study 6 regression results, East Asian participant sub-analysis. The effect of changes in perceived advantaged ingroup resource access on changes in voting preference when accounting for ideological beliefs.*

	Dependent variable: **Δ** Vote preference													
	**Model 1**							**Model 2**						
Predictors	Estimates	*SE*	*OR*	*95% CI*	*z*	*p*	*Response*	*Estimates*	*SE*	*OR*	*95% CI*	*z*	*p*	*Response*
(Intercept)	-2.19	0.22	0.11	[0.07, 0.17]	-10.06	**<.001**	Y-N	-2.29	0.25	0.10	[0.06, 0.17]	-9.17	**<.001**	Y-N
	-2.86	0.31	0.06	[0.03, 0.11]	-9.17	**<.001**	N-Y	-3.61	0.50	0.03	[0.01, 0.07]	-7.27	**<.001**	N-Y
**Δ** Perceived advantaged ingroup outcome	-0.06	0.19	0.94	[0.65, 1.36]	-0.33	.74	Y-N	-0.07	0.19	0.93	[0.64, 1.36]	-0.38	.70	Y-N
	0.590	0.28	1.81	[1.05, 3.13]	2.14	**.03**	N-Y	0.59	0.29	1.80	[1.01, 3.19]	2.02	**.045**	N-Y
Explicit prejudice								-0.08	0.17	0.92	[0.66, 1.28]	-0.49	.63	Y-N
								0.55	0.33	1.73	[0.91, 3.29]	1.67	.10	N-Y
SDO								0.10	0.25	1.10	[0.67, 1.80]	0.39	.70	Y-N
								1.19	0.38	3.29	[1.56, 6.96]	3.13	**.002**	N-Y
SJB								0.25	0.29	1.28	[0.73, 2.26]	0.86	.39	Y-N
								-0.53	0.38	0.59	[0.28, 1.25]	-1.39	.17	N-Y
ZSB								0.31	0.21	1.36	[0.89, 2.08]	1.45	.15	Y-N
								-0.53	0.30	0.59	[0.33, 1.05]	-1.80	.07	N-Y
Political orientation								-0.06	0.21	0.94	[0.62, 1.42]	-0.30	.77	Y-N
								0.53	0.33	1.70	[0.89, 3.22]	1.63	.11	N-Y
Observations	250							250						
df	246							236						
R^2 Nagelkerke	0.031							0.141						
AIC	266.06							266.95						

Note. Results are from multinomial logistic regression models. **Δ** Perceived advantaged ingroup resource access denotes change in perceived advantaged ingroup resource access (T2 – T1). All ideological variables were mean-centered. SDO = social dominance orientation; SJB = system justifying beliefs; ZSB = global zero-sum beliefs. Y-N indicates a change from a "Yes" to a "No" vote. N-Y indicates a change from a "No" to a "Yes" vote. OR = Odds Ratio.

Table S24. *Study 6 regression results, South Asian participant sub-analysis. The effect of changes in perceived advantaged ingroup resource access on changes in voting preference when accounting for ideological beliefs.*

	Dependent variable: Δ Vote preference													
	**Model 1**							**Model 2**						
Predictors	Estimates	*SE*	*OR*	*95% CI*	*z*	*p*	*Response*	*Estimates*	*SE*	*OR*	*95% CI*	*z*	*p*	*Response*
(Intercept)	-3.14	0.69	0.04	[0.01, 0.17]	-4.57	**<.001**	Y-N	-4.76	1.48	0.009	[0.0004, 0.17]	-3.21	**.002**	Y-N
	-1.75	0.34	0.17	[0.09, 0.34]	-5.12	**<.001**	N-Y	-2.26	0.56	0.11	[0.03, 0.32]	-4.06	**<.001**	N-Y
Δ Perceived advantaged ingroup outcome	-0.89	0.36	0.41	[0.20, 0.83]	-2.51	**.02**	Y-N	-1.07	0.47	0.34	[0.14, 0.87]	-2.30	**.03**	Y-N
	-0.052	0.27	0.95	[0.55, 1.63]	-0.19	.85	N-Y	0.19	0.35	1.21	[0.60, 2.44]	0.55	.58	N-Y
Explicit prejudice								-0.03	0.51	0.97	[0.35, 2.68]	-0.05	.96	Y-N
								-0.75	0.31	0.47	[0.26, 0.88]	-2.44	**.02**	N-Y
SDO								1.46	1.32	4.32	[0.31, 60.43]	1.11	.27	Y-N
								-0.18	0.51	0.84	[0.30, 2.32]	-0.35	.73	N-Y
SJB								-1.20	0.90	0.30	[0.05, 1.83]	-1.33	.19	Y-N
								-0.07	0.45	0.94	[0.38, 2.32]	-0.15	.88	N-Y
ZSB								1.64	0.92	5.15	[0.82, 32.47]	1.78	.08	Y-N
								0.81	0.49	2.25	[0.84, 6.02]	1.65	.10	N-Y
Political orientation								-0.33	0.56	0.72	[0.24, 2.18]	-0.60	.55	Y-N
								0.26	0.42	1.29	[0.56, 2.97]	0.62	.54	N-Y
N	73							73						
df	69							59						
R^2 Nagelkerke	0.156							0.394						
AIC	92.52							96.86						

Note. Results are from multinomial logistic regression models. **Δ** Perceived advantaged ingroup resource access denotes change in perceived advantaged ingroup resource access (T2 – T1). All ideological variables were mean-centered. SDO = social dominance orientation; SJB = system justifying beliefs; ZSB = global zero-sum beliefs. Y-N indicates a change from a "Yes" to a "No" vote (*n* = 5). N-Y indicates a change from a "No" to a "Yes" vote (*n* = 10). Participants who did not change their vote across timepoints (*n* = 58) were used as the baseline in all models. Results from this analysis should be interpreted with caution given the lack of power due to the small sub-sample population. OR = Odds Ratio.

Table S25. *Study 7 regression results. The effect of policy condition on perceived advantaged ingroup resource access (left), policy support (middle), and vote (right) when accounting for ideological beliefs.*

	**DV:** Perceived advantaged ingroup resource access					**DV:** Policy support					**DV:** Vote					
Predictors	*Estimates*	*SE*	*t*	*p*	*95% CI*	*Estimates*	*SE*	*t*	*p*	*95% CI*	*Estimate*	*SE*	*z*	*p*	*OR*	*95% CI*
(Intercept | Equality-enhancing condition)	-0.98	0.11	-8.89	**<.001**	[-1.20, -0.77]	4.06	0.13	32.07	**<.001**	[3.81, 4.31]	0.24	0.15	1.59	.11	1.27	[0.95, 1.70]
Inequality-enhancing condition	1.68	0.16	10.68	**<.001**	[1.37, 1.99]	-0.56	0.18	-3.05	**.002**	[-0.93, -0.20]	-0.88	0.23	-3.90	**<.001**	0.42	[0.27, 0.65]
Perceived advantaged ingroup resource access	–	–	–	–	–	0.38	0.05	7.89	**<.001**	[0.28, 0.47]	0.41	0.06	6.91	**<.001**	1.51	[1.34, 1.69]
Ingroup identification	-0.003	0.004	-0.80	.42	[-0.01, 0.005]	0.01	0.00	1.61	.11	[-0.002, 0.02]	0.00	0.01	0.66	.51	1.00	[0.99, 1.01]
SDO	-0.15	0.08	-1.88	.06	[-0.31, 0.007]	0.13	0.09	1.48	.14	[-0.04, 0.29]	0.10	0.10	0.97	.33	1.10	[0.90, 1.35]
SJB	0.15	0.09	1.74	.08	[-0.02, 0.32]	0.18	0.09	1.96	.051	[-0.001, 0.36]	0.20	0.11	1.85	.07	1.22	[0.99, 1.51]
ZSB	0.08	0.07	1.24	.22	[-0.05, 0.21]	0.13	0.07	1.82	.07	[-0.01, 0.27]	0.18	0.08	2.10	**.035**	1.19	[1.01, 1.4]
Political orientation	-0.02	0.06	-0.35	.73	[-0.14, 0.10]	0.14	0.06	2.17	**.031**	[0.01, 0.26]	0.15	0.08	1.92	.055	1.16	[1.00, 1.34]
N	496					495					496					
df	489					487					488					
R^2	0.204					0.138					0.123					
R^2 adjusted	0.195					0.126										

Note. Results from linear regression (perceived advantaged ingroup resource access, policy support) and logistic regression (vote). R^2 for the model with vote as the dependent variable is R^2 Tjur. OR = Odds Ratio. Vote was coded such that 0=Against, 1=In favor. All ideological variables were mean-centered. SDO = social dominance orientation; SJB = system justifying beliefs; ZSB = global zero-sum beliefs.

Table S26. *Study 7 moderation results. Interaction between effects of policy condition x ideological beliefs on perceived advantaged ingroup resource access.*

	**DV:** Perceived advantaged ingroup resource access				
Predictors	*Estimates*	*SE*	*t*	*p*	*95% CI*
(Intercept | Win-win equality-enhancing condition)	-1.00	0.11	-9.12	**<.001**	[-1.22, -0.78]
Lose-lose inequality-enhancing condition	1.68	0.16	10.83	**<.001**	[1.38, 1.99]
SDO	-0.15	0.11	-1.43	.15	[-0.36, 0.06]
SJB	0.38	0.12	3.25	**.001**	[0.15, 0.60]
ZSB	0.20	0.09	2.15	**.032**	[0.02, 0.38]
Political orientation	-0.03	0.08	-0.32	.75	[-0.19, 0.14]
Policy condition X SDO	-0.02	0.16	-0.10	.92	[-0.33, 0.30]
Policy condition X SJB	-0.51	0.17	-3.06	**.002**	[-0.84, -0.18]
Policy condition X ZSB	-0.26	0.13	-1.98	**.048**	[-0.52, -0.002]
Policy condition X Political orientation	0.03	0.12	0.27	.79	[-0.20, 0.27]
N	496				
df	486				
R^2	0.23				
R^2_{adj}	0.21				

Note. Results are from linear mixed regression models. All ideological variables were mean-centered and entered as simultaneous moderator. SJB = system justifying beliefs. ZSB = global zero-sum beliefs. Participants indicated explicit prejudice towards the disadvantaged group in each policy vignette they were presented.

Table S27. *Study 8 regression results. The effect of policy condition on perceived advantaged ingroup resource access (left), policy support (middle), and vote (right) when accounting for ideological beliefs.*

	**DV:** Perceived advantaged ingroup resource access					**DV:** Policy support					**DV:** Vote					
Predictors	*Estimates*	*SE*	*t*	*p*	*95% CI*	*Estimates*	*SE*	*t*	*p*	*95% CI*	*Estimate*	*SE*	*z*	*p*	*OR*	*95% CI*
(Intercept | Joint evaluation condition)	-0.21	0.08	-2.65	**.008**	[-0.36, -0.05]	5.30	0.11	47.82	**<.001**	[5.08, 5.52]	2.16	0.21	10.38	**<.001**	8.64	[5.75, 12.98]
Separate evaluation condition	-0.13	0.11	-1.20	.23	[-0.35, 0.08]	-0.58	0.16	-3.66	**<.001**	[-0.88, -0.27]	-1.34	0.25	-5.47	**<.001**	0.26	[0.16, 0.42]
Perceived advantaged ingroup resource access	—	—	—	—	—	0.49	0.06	7.61	**<.001**	[0.36, 0.62]	0.40	0.09	4.28	**<.001**	1.49	[1.24, 1.79]
Ingroup identification	0.01	0.00	1.75	.08	[-0.001, 0.01]	0.00	0.00	-0.91	.36	[-0.01, 0.01]	-0.009	0.01	-1.31	.19	0.99	[0.98, 1.00]
SDO	0.06	0.05	1.21	.23	[-0.04, 0.17]	-0.32	0.07	-4.26	**<.001**	[-0.47, -0.17]	-0.28	0.11	-2.60	**.009**	0.76	[0.61, 0.93]
SJB	0.11	0.06	1.73	.08	[-0.01, 0.23]	-0.06	0.09	-0.73	.48	[-0.23, 0.11]	-0.08	0.13	-0.66	.51	0.92	[0.72, 1.18]
ZSB	0.14	0.05	2.87	**.004**	[0.04, 0.24]	-0.06	0.07	-0.89	.38	[-0.20, 0.08]	-0.13	0.11	-1.23	.22	0.88	[0.72, 1.08]
Political orientation	0.01	0.04	0.30	.76	[-0.07, 0.09]	0.08	0.06	1.38	.17	[-0.03, 0.19]	0.09	0.08	1.07	.29	1.09	[0.93, 1.28]
N	490					485					490					
df	483					477					482					
R^2	0.03					0.18					0.143					
R^2 adjusted	0.022					0.17					–					

Note. Results from linear regression (perceived advantaged ingroup resource access, policy support) or logistic regression (vote) models. R^2 for the model with vote as the dependent variable is R^2 Tjur. OR = Odds Ratio. Vote was coded such that 0=Against, 1=In favor. All ideological variables were mean-centered. SDO = social dominance orientation; SJB = system justifying beliefs; ZSB = global zero-sum beliefs.

Table S28. *Study 8 moderation results. Interaction between effects of policy condition x ideological beliefs on perceived advantaged ingroup resource access.*

	**DV:** Perceived ingroup resource access				
Predictors	*Estimates*	*SE*	*t*	*p*	*95% CI*
(Intercept | Joint evaluation condition)	-0.19	0.08	-2.48	**.014**	[-0.34, -0.04]
Separate evaluation condition	-0.15	0.11	-1.40	.16	[-0.37, 0.06]
Policy condition X SDO	0.15	0.10	1.41	.16	[-0.06, 0.35]
Policy condition X SJB	0.15	0.12	1.27	.20	[-0.08, 0.39]
Policy condition X ZSB	0.30	0.10	3.05	**.002**	[0.11, 0.49]
Policy condition X Political orientation	0.002	0.08	0.02	.98	[-0.16, 0.16]
N	490				
df	480.00				
R^2	0.065				
R^2 adjusted	0.044				

Note. Results are from linear mixed regression models. All ideological variables were mean-centered and entered as simultaneous moderator. SJB = system justifying beliefs. ZSB = global zero-sum beliefs. Participants indicated explicit prejudice towards the disadvantaged group in each policy vignette they were presented.

Table S29. *Study 1a order effects analysis results.*

	DV: Perceived advantaged ingroup resource access											
	Model 1						Model 2					
Predictors	*Estimates*	*SE*	*df*	*t*	*p*	*95% CI*	*Estimates*	*SE*	*df*	*t*	*p*	*95% CI*
(Intercept | Equality-enhancing condition)	-0.73	0.11	225	-6.89	**<.001**	[-0.94, -0.52]	-0.70	0.11	220	-6.60	**<.001**	[-0.91, -0.49]
Status quo condition	0.80	0.14	1756	5.60	**<.001**	[0.52, 1.08]	0.75	0.14	1751	5.30	**<.001**	[0.47, 1.03]
Inequality-enhancing condition	1.39	0.14	1758	9.79	**<.001**	[1.11, 1.67]	1.34	0.14	1752	9.42	**<.001**	[1.06, 1.62]
Vignette order	0.11	0.04	1180	2.87	**.004**	[0.04, 0.19]	0.11	0.04	1177	2.89	**.004**	[0.04, 0.19]
Status quo condition X Vignette order	-0.02	0.06	1180	-0.38	.70	[-0.13, 0.09]	-0.02	0.06	1176	-0.38	.70	[-0.13, 0.09]
Inequality-enhancing condition X Vignette order	-0.21	0.06	1182	-3.72	**<.001**	[-0.32, -0.10]	-0.21	0.06	1178	-3.72	**<.001**	[-0.32, -0.10]
**Random Effects**												
σ^2	0.62						0.62					
$\tau_{00 \text{ResponseId}}$	0.55						0.52					
$\tau_{00 \text{vignette}}$	0.01						0.01					
ICC	0.47						0.46					
$N_{\text{ResponseId}}$	594						593					
N_{vignette}	6						6					
Observations	1782						1779					
Marginal R^2	0.133						0.157					
Conditional R^2	0.542						0.543					

Note. Results are from linear mixed regression models. Models included vignette order as a fixed effect and participant ID as a random effect. Model 1 includes policy condition and vignette order as predictors. Model 2 includes ideological beliefs (SDO, SJB, ZSB, explicit prejudice, and political orientation) as model covariates.

Table S30. *Study 1b order effects analysis results.*

	**DV:** Perceived advantaged ingroup resource access											
	**Model 1**						**Model 2**					
Predictors	*Estimates*	*SE*	*df*	*t*	*p*	*95% CI*	*Estimates*	*SE*	*df*	*t*	*p*	*95% CI*
(Intercept | Equality-enhancing condition)	-0.39	0.11	1155	-3.65	**<.001**	[-0.60, -0.18]	-0.38	0.11	1150	-3.61	**<.001**	[-0.59, -0.18]
Inequality-enhancing condition	1.17	0.15	1155	7.72	**<.001**	[0.87, 1.46]	1.14	0.15	1149	7.60	**<.001**	[0.85, 1.44]
Vignette order	0.02	0.041	796	0.49	.62	[-0.06, 0.10]	0.02	0.04	793	0.53	.59	[-0.06, 0.10]
Policy condition X Vignette order	-0.05	0.058	796	-0.83	.41	[-0.16, 0.07]	-0.05	0.06	793	-0.85	.40	[-0.16, 0.06]
**Random Effects**												
σ^2	0.66						0.66					
$\tau_{00\text{ResponseId}}$	0.73						0.71					
$\tau_{00\text{vignette}}$	<.001						<.001					
$N_{\text{ResponseId}}$	399						398					
N_{vignette}	6						6					
Observations	1197						1194					
Marginal R^2	0.302						0.328					

Note. Results are from linear mixed regression models. Models included vignette order as a fixed effect and participant ID as a random effect. Model 1 includes policy condition and vignette order as predictors. Model 2 includes ideological beliefs (SDO, SJB, ZSB, explicit prejudice, and political orientation) as model covariates.

Table S31. *Study 2 order effects analysis results*

	**DV:** Perceived advantaged ingroup resource access											
	**Model 1**						**Model 2**					
Predictors	*Estimates*	*SE*	df	*t*	*p*	*95% CI*	*Estimates*	*SE*	df	*t*	*p*	*95% CI*
(Intercept | Intergroup policy condition)	-0.45	0.11	31.7	-3.89	**<.001**	[-0.67, -0.22]	-0.45	0.11	30.1	-3.92	**<.001**	[-0.67, -0.23]
Ingroup policy condition	0.30	0.14	1099	2.08	**.037**	[0.02, 0.58]	0.29	0.14	1101	2.03	**.043**	[0.01, 0.57]
Vignette order	0.06	0.04	771	1.69	.09	[-0.01, 0.14]	0.07	0.04	766	1.71	.09	[-0.01, 0.14]
Policy X Vignette order	-0.05	0.05	771	-1.01	.31	[-0.16, 0.05]	-0.06	0.05	766	-1.03	.30	[-0.16, 0.05]
**Random Effects**												
σ^2	0.56						0.57					
$\tau_{00 \text{ResponseId}}$	0.7						0.63					
$\tau_{00 \text{ vignette}}$	0.01						0.01					
ICC	0.56						0.53					
$N_{\text{ResponseId}}$	387						385					
N_{vignette}	3						3					
Observations	1161						1155					
Marginal R^2	0.01						0.062					
Conditional R^2	0.56						0.56					

Note. Results are from linear mixed regression models. Models included vignette order as a fixed effect and participant ID as a random effect. Model 1 includes policy condition and vignette order as predictors. Model 2 includes ideological beliefs (SDO, SJB, ZSB, explicit prejudice, and political orientation) as model covariates.

Table S32. *Study 3 order effects analysis results*

	**DV:** Perceived advantaged ingroup resource access											
	**Model 1**						**Model 2**					
Predictors	*Estimates*	*SE*	*df*	*t*	*p*	*95% CI*	*Estimates*	*SE*	*df*	*t*	*p*	*95% CI*
(Intercept | Equality-enhancing condition)	-0.43	0.11	236	-3.77	**<.001**	[-0.65, -0.21]	-0.41	0.11	253	-3.61	**<.001**	[-0.63, -0.19]
Inequality-enhancing condition	0.61	0.15	1151	3.97	**<.001**	[0.31, 0.92]	0.57	0.15	1147	3.70	**<.001**	[0.27, 0.87]
Vignette order	0.05	0.04	781	1.17	.24	[-0.03, 0.13]	0.05	0.04	780	1.18	.24	[-0.03, 0.13]
Policy X Vignette order	-0.13	0.06	782	-2.18	**.029**	[-0.25, -0.01]	-0.13	0.06	783	-2.19	**.029**	[-0.25, -0.01]
**Random Effects**												
σ^2	0.7						0.7					
$\tau_{00 \text{ResponseId}}$	0.7						0.66					
$\tau_{00 \text{vignette}}$	0.01						0.01					
ICC	0.5						0.49					
$N_{\text{ResponseId}}$	393						393					
N_{vignette}	6						6					
Observations	1178						1178					
Marginal R^2	0.02						0.06					
Conditional R^2	0.52						0.52					

Note. Results are from linear mixed regression models. Models included vignette order as a fixed effect and participant ID as a random effect. Model 1 includes policy condition and vignette order as predictors. Model 2 includes ideological beliefs (SDO, SJB, ZSB, explicit prejudice, and political orientation) as model covariates.

Table S33. *Study 4 order effects analysis results.*

	**DV:** Perceived advantaged ingroup resource access											
	**Model 1**						**Model 2**					
Predictors	*Estimates*	*SE*	*df*	*t*	*p*	*95% CI*	*Estimates*	*SE*	*df*	*t*	*p*	*95% CI*
(Intercept | Equality-enhancing, Limited condition)	-0.28	0.15	642	-1.82	.07	[-0.58, 0.02]	-0.28	0.15	651	-1.81	.07	[-0.57, 0.02]
Inequality-enhancing condition	0.89	0.21	1160	4.15	**<.001**	[0.47, 1.30]	0.88	0.21	1156	4.15	**<.001**	[0.47, 1.30]
Unlimited resource condition	-0.11	0.21	1162	-0.51	.61	[-0.52, 0.31]	-0.11	0.21	1157	-0.52	.61	[-0.52, 0.30]
Vignette order	-0.01	0.06	782	-0.17	.87	[-0.13, 0.11]	-0.01	0.06	781	-0.18	.86	[-0.13, 0.11]
Policy X Resource	0.50	0.30	1159	1.66	.10	[-0.09, 1.08]	0.49	0.30	1154	1.63	.10	[-0.09, 1.07]
Policy X Vignette order	-0.03	0.08	781	-0.39	.70	[-0.20, 0.13]	-0.03	0.08	779	-0.39	.70	[-0.20, 0.13]
Resource X Vignette order	0.03	0.08	782	0.37	.71	[-0.13, 0.19]	0.03	0.08	781	0.34	.73	[-0.14, 0.19]
Policy X Resource X Vignette order	-0.10	0.12	781	-0.88	.38	[-0.34, 0.13]	-0.09	0.12	780	-0.80	.43	[-0.33, 0.14]
**Random Effects**												
σ^2	0.69						0.69					
$\tau_{00 \text{ResponseId}}$	0.6						0.58					
$\tau_{00 \text{vignette}}$	<.001						<.001					
ICC	0.47						0.46					
$N_{\text{ResponseId}}$	393						393					
N_{vignette}	6						6					
Observations	1179						1179					
Marginal R^2	0.16						0.18					
Conditional R^2	0.55						0.56					

Note. Results are from linear mixed regression models. Models included vignette order as a fixed effect and participant ID as a random effect. Model 1 includes policy condition and vignette order as predictors. Model 2 includes ideological beliefs (SDO, SJB, ZSB, explicit prejudice, and political orientation) as model covariates.

Table S34. *Study 5 order effects analysis results*

	**DV:** Perceived advantaged ingroup resource access											
	**Model 1**						**Model 2**					
Predictors	*Estimates*	*SE*	*df*	*t*	*p*	*95% CI*	*Estimates*	*SE*	*df*	*t*	*p*	*95% CI*
(Intercept | Limited access condition)	-1.11	0.10	430	-11.39	**<.001**	[-1.30, -0.92]	-1.08	0.10	545	-11.19	**<.001**	[-1.27, -0.89]
Unlimited access condition	0.97	0.14	1180	7.13	**<.001**	[0.70, 1.24]	0.91	0.14	1168	6.66	**<.001**	[0.64, 1.17]
Vignette order	0.03	0.04	796	0.72	.47	[-0.05, 0.10]	0.02	0.04	790	0.65	.52	[-0.05, 0.10]
Policy condition X Vignette order	-0.02	0.05	796	-0.34	.73	[-0.12, 0.09]	-0.02	0.05	790	-0.31	.76	[-0.12, 0.09]
**Random Effects**												
σ^2	0.57						0.57					
$\tau_{00 \text{ResponseId}}$	0.51						0.47					
$\tau_{00 \text{vignette}}$	<.001						<.001					
ICC	0.47						0.45					
$N_{\text{ResponseId}}$	399						396					
N_{vignette}	6						6					
Observations	1197						1188					
Marginal R^2	0.17						0.20					
Conditional R^2	0.56						0.57					

Note. Results are from linear mixed regression models. Models included vignette order as a fixed effect and participant ID as a random effect. Model 1 includes policy condition and vignette order as predictors. Model 2 includes ideological beliefs (SDO, SJB, ZSB, explicit prejudice, and political orientation) as model covariates.

Supplemental Figures

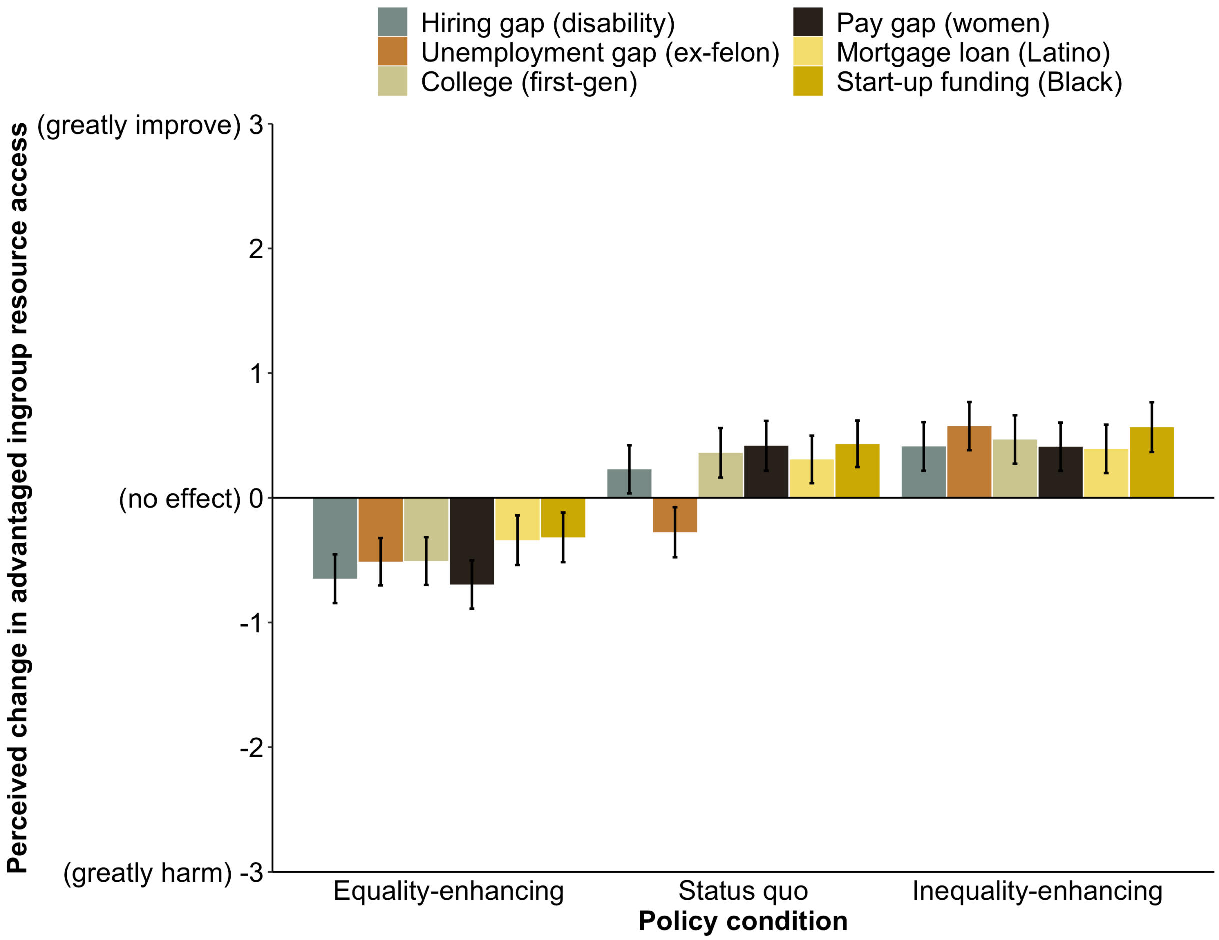

Figure S1. Perceived advantaged ingroup resource access by policy vignette across Study 1a conditions. Hiring gap (disability), Unemployment gap (ex-felon), College (first-gen) were representation-based disparities. Pay gap (women), Mortgage loan (Latino), Start-up funding (Black) were monetary-based disparities. Means are adjusted based on the participant random effect included in the linear mixed model. Bars indicate 95% CIs around the mean.

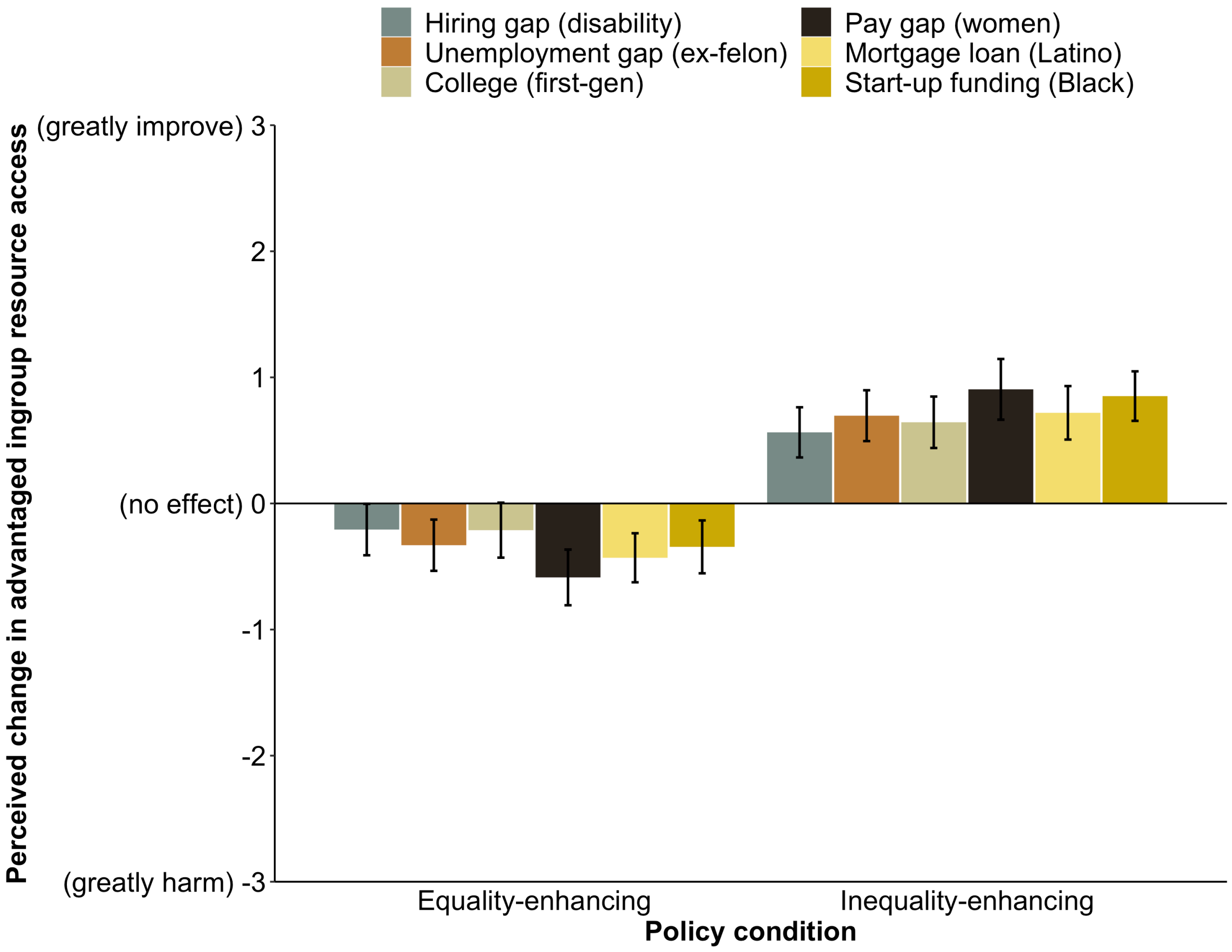

Figure S2. Perceived advantaged ingroup resource access by policy vignette across Study 1b conditions. All vignettes involved representation-based disparities. Means are adjusted based on the participant random effect included in the linear mixed model. Bars indicate 95% CIs around the mean.

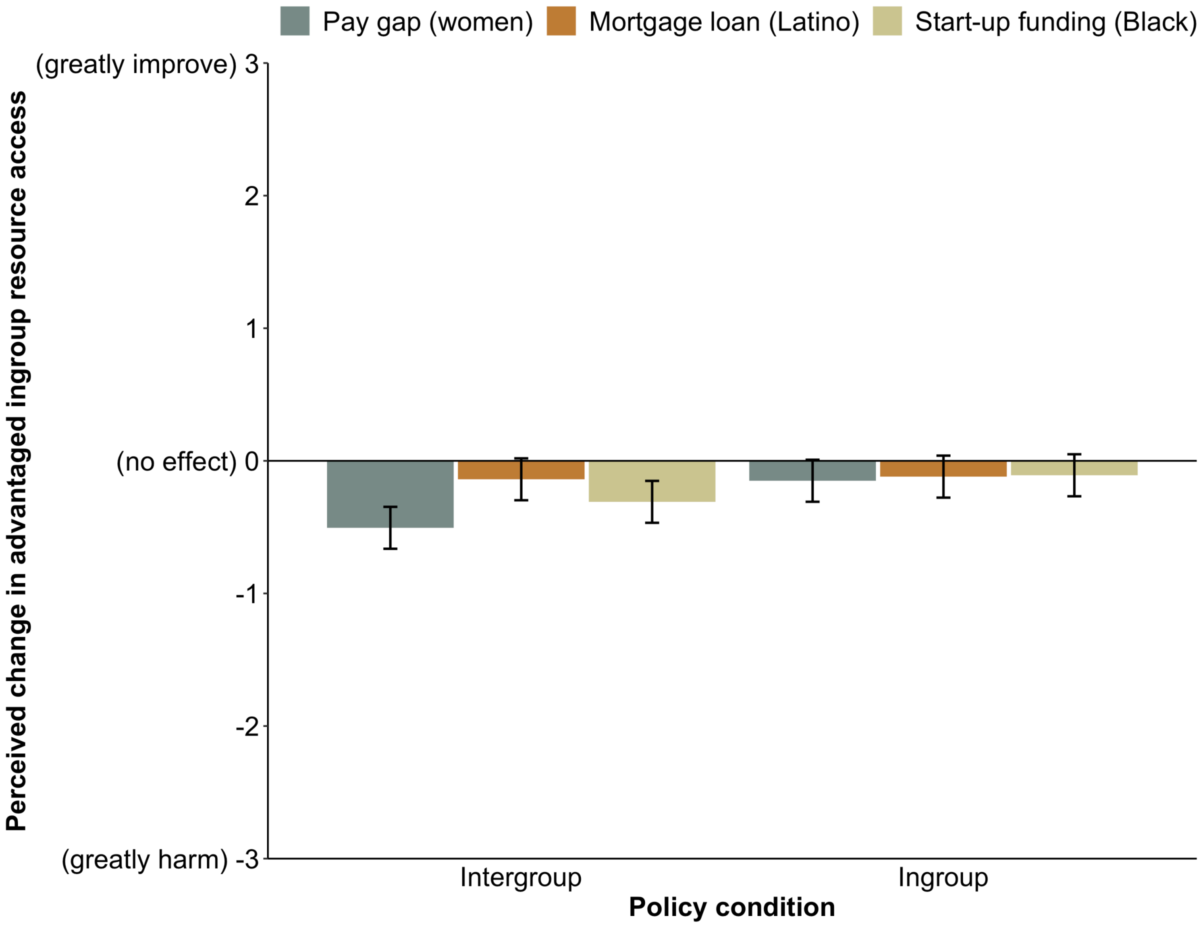

Figure S3. Perceived advantaged ingroup resource access by policy vignette across Study 2 conditions. All vignettes involved monetary-based disparities. Means are adjusted based on the participant random effect included in the linear mixed model. Bars indicate 95% CIs around the mean.

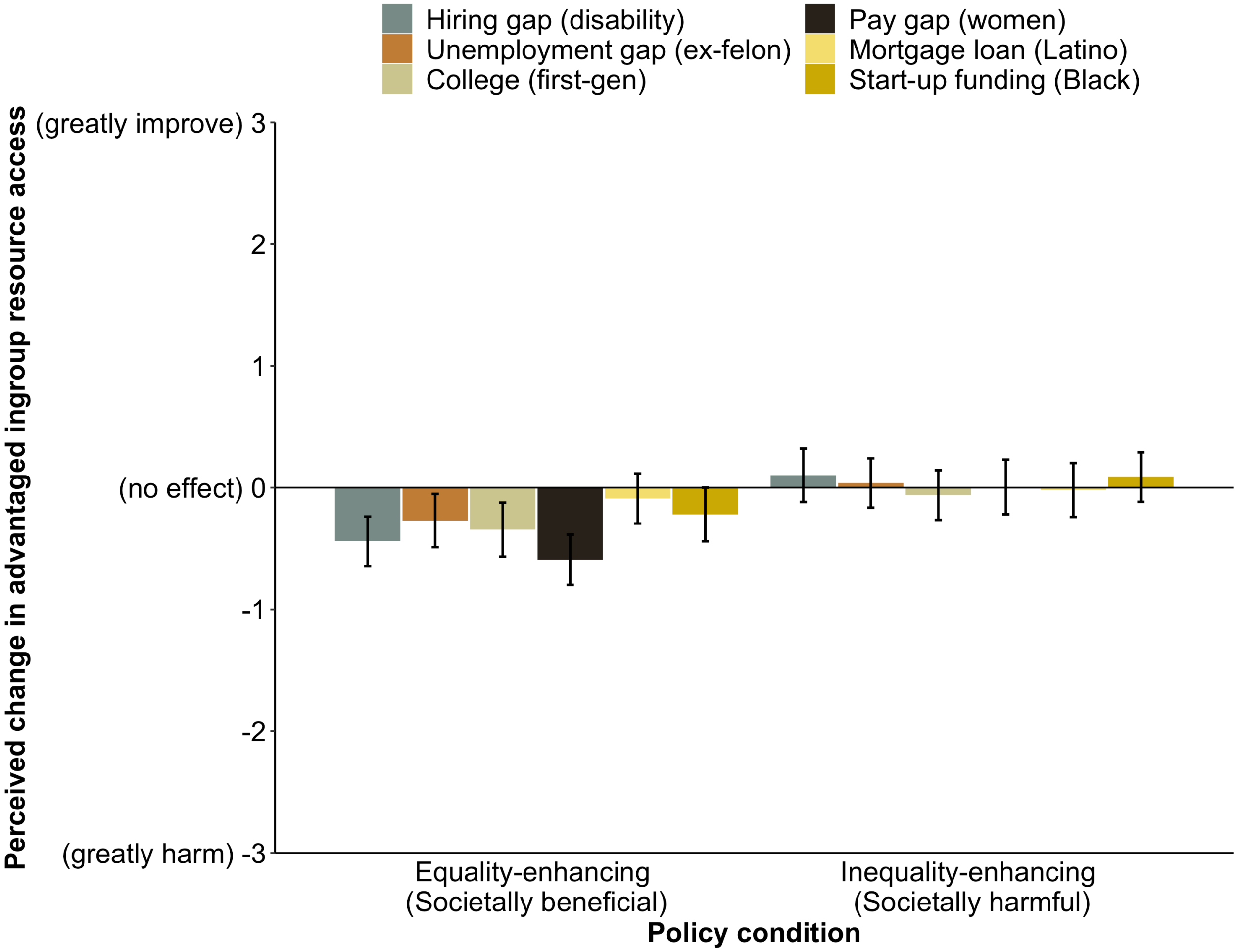

Figure S4. Perceived advantaged ingroup resource access by policy vignette across Study 3 conditions. Hiring gap (disability), Unemployment gap (ex-felon), College (first-gen) were representation-based disparities. Pay gap (women), Mortgage loan (Latino), Start-up funding (Black) were monetary-based disparities. Means are adjusted based on the participant random effect included in the linear mixed model. Bars indicate 95% CIs around the mean.

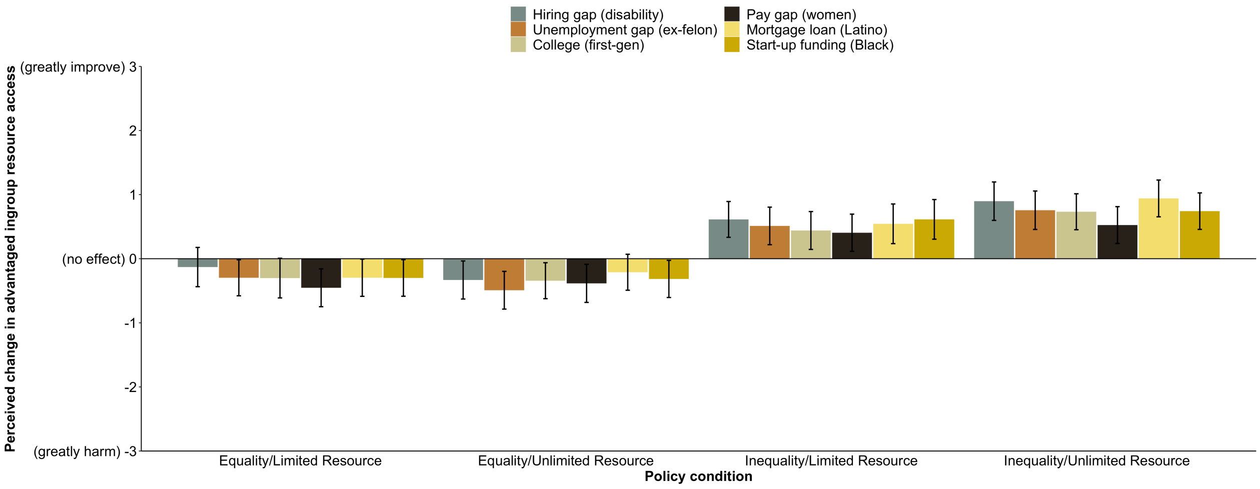

Figure S5. Perceived advantaged ingroup resource access by policy vignette across Study 4 conditions. Hiring gap (disability), Unemployment gap (ex-felon), College (first-gen) were representation-based disparities. Pay gap (women), Mortgage loan (Latino), Start-up funding (Black) were monetary-based disparities. Means are adjusted based on the participant random effect included in the linear mixed model. Bars indicate 95% CIs around the mean.

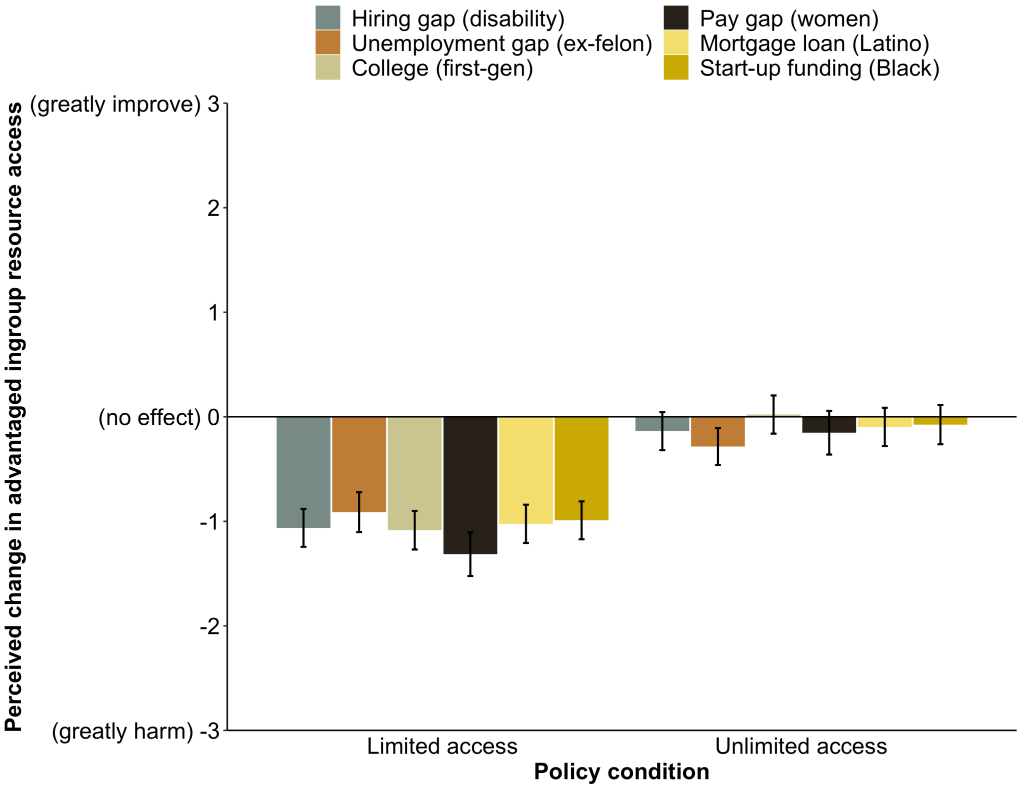

Figure S6. Perceived advantaged ingroup resource access by policy vignette across Study 5 conditions. Hiring gap (disability), Unemployment gap (ex-felon), College (first-gen) were representation-based disparities. Pay gap (women), Mortgage loan (Latino), Start-up funding (Black) were monetary-based disparities. Means are adjusted based on the participant random effect included in the linear mixed model. Bars indicate 95% CIs around the mean.

Supplementary Materials: Policy condition text

Study 1

Mortgage lending gap (White homebuyers | Latino homebuyers)

Equality-enhancing. According to a recent report, in 2018 White homebuyers received roughly $386.4 billion in mortgage loans from banks while Latino buyers only received around $12.6 billion in mortgage loans overall. Several banks propose increasing the total amount of mortgage loans to Latino homebuyers by $7.3 billion and not changing the total amount of mortgage loan funding to White homebuyers. Ultimately, these banks predict that this proposal will narrow the gap in mortgage loans between Latino and White homebuyers over the next year.

Inequality-enhancing. According to a recent report, in 2018 White homebuyers received roughly $386.4 billion in mortgage loans from banks while Latino buyers only received around $12.6 billion in mortgage loans overall. Several banks propose decreasing the total amount of mortgage loans to Latino homebuyers by $7.3 billion and not changing the total amount of mortgage loan funding to White homebuyers. Ultimately, these banks predict that this proposal will widen the gap in mortgage loans between Latino and White homebuyers over the next year.

Status quo. According to a recent report, in 2018 White homebuyers received roughly $386.4 billion in mortgage loans from banks while Latino buyers only received around $12.6 billion in mortgage loans overall. However, several banks propose not changing mortgage loan funding over the next year.

Pay gap (Men | Women)

Equality-enhancing. This week, a joint report from the top tech firms in Silicon Valley revealed that female employees across the industry cumulatively earn $1.93 billion compared to their male employee counterparts who earn an estimated cumulative $25.6 billion. Leaders across these tech firms propose increasing the pay to female employees by $500 million and not changing the pay to male employees. Industry leaders expect that this proposal will reduce the gender pay gap over the next year.

Inequality-enhancing. This week, a joint report from the top tech firms in Silicon Valley revealed that female employees across the industry cumulatively earn $1.93 billion compared to their male employee counterparts who earn an estimated cumulative $25.6 billion. Leaders across these tech firms propose decreasing the pay to female employees by $500 million and not changing the pay to male employees. Industry leaders expect that this proposal will widen the gender pay gap over the next year.

Status quo. This week, a joint report from the top tech firms in Silicon Valley revealed that female employees across the industry cumulatively earn $1.93 billion compared to their male employee counterparts who earn an estimated cumulative $25.6 billion. However, leaders across these firms propose not changing pay to employees over the next year.

Hiring gap (Non-disabled job seekers | Disabled job seekers):

Equality-enhancing. According to the U.S. Department of Labor (DOL), there are roughly 1.4 million non-disabled U.S. federal government employees and only about 405,000 disabled U.S. employees. The DOL proposes to increase the number of disabled employees who are hired across these agencies by 260,000 and not changing the number of non-disabled employees. The DOL predicts that this proposal will decrease the hiring gap between disabled and non-disabled people within government agencies over the next year.

Inequality-enhancing. According to the U.S. Department of Labor (DOL), there are roughly 1.4 million non-disabled U.S. federal government employees and only about 405,000 disabled U.S. employees. The DOL proposes to decrease the number of disabled employees who are hired across these agencies by 260,000 and not changing the number of non-disabled employees. The DOL predicts that this proposal will increase the hiring gap between disabled and non-disabled people within government agencies over the next year.

Status quo. According to the U.S. Department of Labor (DOL), there are roughly 1.4 million non-disabled U.S. federal government employees and only about 405,000 disabled U.S. employees. However, the DOL proposes not changing hiring over the next year.

Unemployment gap (People with no criminal history | People with criminal history)

Equality-enhancing. A recent Prison Policy Initiative (PPI) report revealed that, in the U.S., roughly 21.6 million ex-felon job seekers are unable to find a job compared to the 13.1 million job seekers without a criminal record who are unable to find a job. Various companies across the U.S. propose increasing the number of jobs available for ex-felon job seekers by 3.1 million and not changing the number of jobs available for non-felon job seekers. These companies predict that this proposal will reduce the gap in unemployment between ex-felon and non-felon job seekers over the next year.

Inequality-enhancing. A recent Prison Policy Initiative (PPI) report revealed that, in the U.S., roughly 21.6 million ex-felon job seekers are unable to find a job compared to the 13.1 million job seekers without a criminal record who are unable to find a job. Various companies across the U.S. propose reducing the number of jobs available for ex-felon job seekers by 3.1 million and not changing the number of jobs available for non-felon job seekers. These companies predict that this proposal will increase the gap in unemployment between ex-felons and non-felon job seekers over the next year.

Status quo. A recent Prison Policy Initiative (PPI) report revealed that, in the U.S., roughly 21.6 million ex-felon job seekers are unable to find a job compared to the 13.1 million job seekers without a criminal record who are unable to find a job. However, various companies across the U.S. propose not changing the number of jobs available to job seekers over the next year.

Startup funding gap (White entrepreneur | Black entrepreneur)

Equality-enhancing. A recent report from the U.S. Small Businesses Association (SBA) detailed that Black entrepreneurs receive significantly less funding—an estimated \$12.4 million––to start their businesses compared to White entrepreneurs, who received an estimated \$241.8 million. Several venture capital firms propose increasing their total investments in startups founded by Black entrepreneurs by \$8.2 million and not changing their total investments in startups founded by White entrepreneurs. Ultimately, these firms predict that this proposal will narrow the investment gap in Black and White businesses over the next year.

Inequality-enhancing. A recent report from the U.S. Small Businesses Association (SBA) detailed that Black entrepreneurs receive significantly less funding—an estimated \$12.4 million—to start their businesses compared to White entrepreneurs, who received an estimated \$241.8 million. Several venture capital firms propose decreasing their total investments in startups founded by Black entrepreneurs by \$8.2 million and not changing their total investments in startups founded by White entrepreneurs. Ultimately, these firms predict that this proposal will widen the investment gap in Black and White businesses over the next year.

Status quo. A recent report from the U.S. Small Businesses Association (SBA) detailed that Black entrepreneurs receive significantly less funding—an estimated \$12.4 million—to start their businesses compared to White entrepreneurs, who received an estimated \$241.8 million. However, several venture capital firms propose not changing startup funding over the next year.

College admissions gap (Continuing-generation students | First-generation students)

Equality-enhancing. A recent report from eight accredited U.S. universities revealed that of the 510,000 college freshmen accepted to these universities, 173,000 were first-generation college students and about 337,000 were continuing-generation college students. Administrators from these eight universities propose increasing the number of first-generation students who are admitted by 86,000 and not changing the number of continuing-generation students who are admitted. University leadership predicts that this proposal will decrease the gap in admissions between first-generation and continuing-generation college applicants in the next year.

Inequality-enhancing. A recent report from eight accredited U.S. universities revealed that of the 510,000 college freshmen accepted to these universities, 173,000 were first-generation college students and about 337,000 were continuing-generation college students. Administrators from these eight universities propose decreasing the number of first-generation students who are admitted by 86,000 and not changing the number of continuing-generation students who are admitted. University leadership predicts that this proposal will increase the gap in admissions between first-generation and continuing-generation college applicants in the next year.

Status quo. A recent report from eight accredited U.S. universities revealed that of the 510,000 college freshmen accepted to these universities, 173,000 were first-generation college students and about 337,000 were continuing-generation college students. However, administrators from these eight universities propose not changing their admissions in the next year.

Study 1b

Homeownership gap (White homebuyers | Latino homebuyers)

Equality-enhancing. According to a recent report, in 2018, 144.6 million White people owned their homes while 28.8 million Latino people owned their homes. Several banks propose increasing the number of Latino homeowners by 7.2 million and not changing the proportion of White people who are homeowners. Ultimately, these banks predict that this proposal will narrow the gap in homeownership between Latino and White people over the next year.

Inequality-enhancing. According to a recent report, in 2018, 144.6 million White people owned their homes while 28.8 million Latino people owned their homes. Several banks propose decreasing the number of Latino homeowners by 7.2 million and not changing the proportion of White people who are homeowners. Ultimately, these banks predict that this proposal will increase the gap in homeownership between Latino and White people over the next year.

Hiring gap (men | women)

Equality-enhancing. This week, a joint report from the top tech firms in Silicon Valley revealed that there are 212,000 female employees in the industry compared to their 620,000 male employee counterparts throughout the industry. Leaders across these tech firms propose increasing the number of women who are hired in the industry by 53,000 and not changing the proportion of men who are hired. Industry leaders expect that this proposal will reduce the gender hiring gap over the next year.

Inequality-enhancing. This week, a joint report from the top tech firms in Silicon Valley revealed that there are 212,000 female employees in the industry compared to their 620,000 male employee counterparts throughout the industry. Leaders across these tech firms propose decreasing the number of women who are hired in the industry by 53,000 and not changing the proportion of men who are hired. Industry leaders expect that this proposal will widen the gender hiring gap over the next year.

Startup funding gap (White entrepreneur | Black entrepreneur)

Equality-enhancing. A recent report from the U.S. Small Businesses Association (SBA) detailed that an estimated 837 Black entrepreneurs received funding from venture capital firms compared to 7,533 White entrepreneurs who received capital funding. Several venture capital firms propose investing in 468 additional Black entrepreneurs and not changing the proportion of White entrepreneurs that they invest in. Ultimately, these firms predict that this proposal will narrow the investment gap in Black and White businesses over the next year.

Inequality-enhancing. A recent report from the U.S. Small Businesses Association (SBA) detailed that an estimated 837 Black entrepreneurs received funding from venture capital firms compared to 7,533 White entrepreneurs who received capital funding. Several venture capital firms propose investing in 468 fewer Black entrepreneurs and not changing the

proportion of White entrepreneurs that they invest in. Ultimately, these firms predict that this proposal will widen the investment gap in Black and White businesses over the next year.

Hiring gap (People w/o disability | People w/ disability)

Equality-enhancing. According to the U.S. Department of Labor (DOL), there are roughly 1.4 million non-disabled U.S. federal government employees and only about 405,000 disabled U.S. employees. The DOL proposes to increase the number of disabled employees who are hired across these agencies by 260,000 and not changing the proportion of non-disabled employees. The DOL predicts that this proposal will decrease the hiring gap between disabled and non-disabled people within government agencies over the next year.

Inequality-enhancing. According to the U.S. Department of Labor (DOL), there are roughly 1.4 million non-disabled U.S. federal government employees and only about 405,000 disabled U.S. employees. The DOL proposes to decrease the number of disabled employees who are hired across these agencies by 260,000 and not changing the proportion of non-disabled employees. The DOL predicts that this proposal will increase the hiring gap between disabled and non-disabled people within government agencies over the next year.

Unemployment gap (People w/o criminal history | People w/ criminal history)

Equality-enhancing. A recent Prison Policy Initiative (PPI) report revealed that, in the U.S., roughly 21.6 million ex-felon job seekers are unable to find a job compared to the 13.1 million job seekers without a criminal record who are unable to find a job. Various companies across the U.S. propose increasing the number of jobs available for ex-felon job seekers by 3.1 million and not changing the proportion of jobs available for non-felon job-seekers. These companies predict that this proposal will reduce the gap in unemployment between ex-felon and non-felon job seekers over the next year.

Inequality-enhancing. A recent Prison Policy Initiative (PPI) report revealed that, in the U.S., roughly 21.6 million ex-felon job seekers are unable to find a job compared to the 13.1 million job seekers without a criminal record who are unable to find a job. Various companies across the U.S. propose reducing the number of jobs available for ex-felon job seekers by 3.1 million and not changing the proportion of jobs available for non-felon job seekers. These companies predict that this proposal will increase the gap in unemployment between ex-felons and non-felon job seekers over the next year.

College admissions gap (Continuing-generation students | First-generation students)

Equality-enhancing. A recent report from eight accredited U.S. universities revealed that of the 510,000 college freshmen accepted to these universities, 173,000 were first-generation college students and about 337,000 were continuing-generation college students. Administrators from these eight universities propose increasing the number of first-generation students who are admitted by 86,000 and not changing the proportion of continuing-generation students who are admitted. University leadership predicts that this proposal will decrease the gap in admissions between first-generation and continuing-generation college applicants in the next year.

Inequality-enhancing. A recent report from eight accredited U.S. universities revealed that of the 510,000 college freshmen accepted to these universities, 173,000 were first-generation college students and about 337,000 were continuing-generation college students. Administrators from these eight universities propose decreasing the number of first-generation students who are admitted by 86,000 and not changing the proportion of continuing-generation students who are admitted. University leadership predicts that this proposal will increase the gap in admissions between first-generation and continuing-generation college applicants in the next year.

Study 2

Mortgage lending gap (White homebuyers | Latino homebuyers)

Intergroup equality-enhancing. According to a 2019 report, while White homebuyers in a neighborhood received an average of $273,000 in mortgage loans from banks, comparable Latino homebuyers in the same neighborhood received an average of $249,000 in mortgage loans. There was no available explanation for this gap. Several banks propose increasing mortgage loans by an average of $24,000 to Latino homebuyers who tend to receive less and not changing the total amount of mortgage loan funding to White homebuyers. Ultimately, these banks predict that this proposal will erase the gap in mortgage loans between these homebuyers over the next year.

Ingroup equality-enhancing. According to a 2019 report, while most White homebuyers in a neighborhood received an average of $273,000 in mortgage loans from banks, a group of comparable White homebuyers in the same neighborhood received an average of $249,000 in mortgage loans. There was no available explanation for this gap. Several banks propose increasing mortgage loans by an average of $24,000 to the group of White homebuyers who tend to receive less and not changing the total amount of mortgage loan funding to the other White homebuyers. Ultimately, these banks predict that this proposal will erase the gap in mortgage loans between these homebuyers over the next year.

Pay gap (Men | Women)

Intergroup equality-enhancing. This week, a joint report from the top tech firms in Silicon Valley revealed that female software engineers earned an average salary of $112,000 in 2019, compared to male software engineers with the same work experience who earned $129,000 on average. Leaders across these tech firms propose increasing the salary by an average of $17,000 for female software engineers who tend to receive less and not changing the salary for male software engineers. Industry leaders expect that this proposal will wipe out the pay gap between these software engineers over the next year.

Ingroup equality-enhancing. This week, a joint report from the top tech firms in Silicon Valley revealed that a group of male software engineers earned an average salary of $112,000 compared to other male software engineers with the same work experience who earned $129,000 on average. Leaders across these tech firms propose increasing the salary by an average of $17,000 for the group of male software engineers who tend to receive less and not changing the salary to the other male software engineers. Industry leaders expect that this proposal will wipe out the pay gap between these software engineers over the next year.

Startup funding gap (White entrepreneur | Black entrepreneur)

Intergroup equality-enhancing. A recent report from the U.S. Small Businesses Association (SBA) detailed that White entrepreneurs received an average of $15.8 million in venture capital funding in 2019 compared to an average of $9.48 million to comparable Black entrepreneurs. The report showed that there were no differences between the funded companies.

Several venture capital firms propose increasing investments by an average of $6.32 million for companies founded by Black entrepreneurs who tend to receive less and not changing investments in companies founded by White entrepreneurs. Ultimately, these firms predict that this proposal will close the investment gap between these entrepreneurs over the next year.

Ingroup equality-enhancing. A recent report from the U.S. Small Businesses Association (SBA) detailed that White entrepreneurs received an average of $15.8 million in venture capital funding in 2019 compared to an average of $9.48 million to comparable Black entrepreneurs. The report showed that there were no differences between the funded companies. Several venture capital firms propose increasing investments by an average of $6.32 million for companies founded by the group of White entrepreneurs who tend to receive less and not changing investments in companies founded by the other White entrepreneurs. Ultimately, these firms predict that this proposal will close the investment gap between these entrepreneurs over the next year.

Study 3

Mortgage lending gap (White homebuyers | Latino homebuyers)

Equality-enhancing (societally beneficial). According to a recent report, in 2018 White homebuyers received roughly $386.4 billion in mortgage loans from banks while Latino buyers only received around $12.6 billion in mortgage loans overall.

Several banks propose increasing the total amount of mortgage loans to Latino homebuyers by $7.3 billion and not changing the total amount of mortgage loan funding to White homebuyers. Ultimately, these banks predict that this proposal will narrow the gap in mortgage loans between Latino and White homebuyers over the next year. These banks stated that this policy will have the additional effect of stimulating greater mortgage investment nationwide, increasing the total benefits for homebuyers of all racial groups.

Inequality-enhancing (societally harmful). According to a recent report, in 2018 White homebuyers received roughly $386.4 billion in mortgage loans from banks while Latino buyers only received around $12.6 billion in mortgage loans overall.

Several banks propose decreasing the total amount of mortgage loans to Latino homebuyers by $7.3 billion and not changing the total amount of mortgage loan funding to White homebuyers. Ultimately, these banks predict that this proposal will widen the gap in mortgage loans between Latino and White homebuyers over the next year. These banks stated that this policy will have the additional effect of reducing mortgage investment nationwide, decreasing the total benefits for homebuyers of all racial groups.

Pay gap (Men | Women)

Equality-enhancing (societally beneficial). This week, a joint report from the top tech firms in Silicon Valley revealed that female employees across the industry cumulatively earn $1.93 billion compared to their male employee counterparts who earn an estimated cumulative $25.6 billion.

Leaders across these tech firms propose increasing the total pay to female employees by $500 million and not changing total pay to male employees. Industry leaders expect that this proposal will reduce the gender pay gap over the next year. These leaders stated that this policy will have the additional effect of boosting employee salaries across the industry, increasing the total pay for employees regardless of gender.

Inequality-enhancing (societally harmful). This week, a joint report from the top tech firms in Silicon Valley revealed that female employees across the industry cumulatively earn $1.93 billion compared to their male employee counterparts who earn an estimated cumulative $25.6 billion.

Leaders across these tech firms propose decreasing the total pay to female employees by $500 million and not changing total pay to male employees. Industry leaders expect that this proposal will widen the gender pay gap over the next year. These leaders stated that this policy will have the additional effect of reducing employee salaries across the industry, decreasing the total pay for employees regardless of gender.

Hiring gap (Non-disabled job seekers | Disabled job seekers)

Equality-enhancing (societally beneficial). According to the U.S. Department of Labor (DOL), there are roughly 1.4 million non-disabled U.S. federal government employees and only about 405,000 disabled U.S. employees. The DOL proposes to increase the number of disabled employees who are hired across these agencies by 260,000 and not changing the number of non-disabled employees. The DOL predicts that this proposal will reduce the hiring gap between disabled and non-disabled people within government agencies over the next year. The DOL stated that this policy will have the additional effect of stimulating hiring across government agencies, increasing the total number of new employees regardless of disability status.

Inequality-enhancing (societally harmful). According to the U.S. Department of Labor (DOL), there are roughly 1.4 million non-disabled U.S. federal government employees and only about 405,000 disabled U.S. employees. The DOL proposes to decrease the number of disabled employees who are hired across these agencies by 260,000 and not changing the number of non-disabled employees. The DOL predicts that this proposal will increase the hiring gap between disabled and non-disabled people within government agencies over the next year. The DOL stated that this policy will have the additional effect of reducing hiring across government agencies, decreasing the total number of new employees regardless of disability status.

Unemployment gap (People with no criminal history | People with criminal history)

Equality-enhancing (societally beneficial). A recent Prison Policy Initiative (PPI) report revealed that, in the U.S., roughly 21.6 million ex-felon job seekers are unable to find a job compared to the 13.1 million job seekers without a criminal record who are unable to find a job. Various companies across the U.S. propose increasing the number of jobs available for ex-felon job seekers by 3.1 million and not changing the number of jobs available for non-felon job-seekers. These companies predict that this proposal will reduce the gap in unemployment between ex-felon and non-felon job seekers over the next year. These companies stated that this policy will have the additional effect of stimulating job growth nationwide, increasing the total number of new hires regardless of criminal history.

Inequality-enhancing (societally harmful). A recent Prison Policy Initiative (PPI) report revealed that, in the U.S., roughly 21.6 million ex-felon job seekers are unable to find a job compared to the 13.1 million job seekers without a criminal record who are unable to find a job. Various companies across the U.S. propose reducing the number of jobs available for ex-felon job seekers by 3.1 million and not changing the number of jobs available for non-felon job seekers. These companies predict that this proposal will increase the gap in unemployment between ex-felons and non-felon job seekers over the next year. These companies stated that this policy will have the additional effect of reducing job growth nationwide, decreasing the total number of new hires regardless of criminal history.

Startup funding gap (White entrepreneur | Black entrepreneur)

Equality-enhancing (societally beneficial). A recent report from the U.S. Small Businesses Association (SBA) detailed that Black entrepreneurs receive significantly less

funding—an estimated $12.4 million—to start their businesses compared to White entrepreneurs, who received an estimated $241.8 million. Several venture capital firms propose increasing their total investments in startups founded by Black entrepreneurs by $8.2 million and not changing their total investments in startups founded by White entrepreneurs. Ultimately, these firms predict that this proposal will narrow the investment gap between Black and White businesses over the next year. These firms stated that this policy will have the additional effect of stimulating capital investments nationwide, increasing the total benefits for entrepreneurs of all racial groups.

Inequality-enhancing (societally harmful). A recent report from the U.S. Small Businesses Association (SBA) detailed that Black entrepreneurs receive significantly less funding—an estimated $12.4 million—to start their businesses compared to White entrepreneurs, who received an estimated $241.8 million. Several venture capital firms propose decreasing their total investments in startups founded by Black entrepreneurs by $8.2 million and not changing their total investments in startups founded by White entrepreneurs. Ultimately, these firms predict that this proposal will widen the investment gap between Black and White businesses over the next year. These firms stated that this policy will have the additional effect of reducing capital investments nationwide, decreasing the total benefits for entrepreneurs of all racial groups.

College admissions gap (Continuing-generation students | First-generation students)

Equality-enhancing (societally beneficial). A recent report from eight accredited U.S. universities revealed that of the 510,000 college freshmen accepted to these universities, 173,000 were first-generation college students and about 337,000 were continuing-generation college students. Administrators from these eight universities propose increasing the number of first-generation students who are admitted by 86,000 and not changing the number of continuing-generation students who are admitted. University leadership predicts that this proposal will decrease the gap in admissions between first-generation and continuing-generation college applicants in the next year. University leadership stated that this policy will lead other U.S. colleges to increase overall college admissions, increasing the total benefits for both first- and continuing-generation applicants.

Inequality-enhancing (societally harmful). A recent report from eight accredited U.S. universities revealed that of the 510,000 college freshmen accepted to these universities, 173,000 were first-generation college students and about 337,000 were continuing-generation college students. Administrators from these eight universities propose decreasing the number of first-generation students who are admitted by 86,000 and not changing the number of continuing-generation students who are admitted. University leadership predicts that this proposal will increase the gap in admissions between first-generation and continuing-generation college applicants in the next year. University leadership stated that this policy will lead other U.S. colleges to decrease overall college admissions, decreasing the total benefits for both first- and continuing-generation applicants.

Study 4

Mortgage lending gap (White homebuyers | Latino homebuyers)

Equality-enhancing, Limited [*Unlimited*] resource. According to a recent report, in 2018 White homebuyers received roughly $386.4 billion in mortgage loans from banks while Latino buyers only received around $12.6 billion in mortgage loans overall. Several banks propose increasing the total amount of mortgage loans to Latino homebuyers by $7.3 billion and not changing the total amount of mortgage loan funding to White homebuyers. Ultimately, these banks predict that this proposal will narrow the gap in mortgage loans between Latino and White homebuyers in the next year. These banks reported there will be no change in profits for many years, and they will need to reorganize their budgets to fund these mortgage loans. [*These banks reported there have been large and consistent increases in profits that will continue for many years, allowing them to fund mortgage loans for as many people as they want.*]

Inequality-enhancing, Limited [*Unlimited*] resource. According to a recent report, in 2018 White homebuyers received roughly $386.4 billion in mortgage loans from banks while Latino buyers only received around $12.6 billion in mortgage loans overall. Several banks propose decreasing the total amount of mortgage loans to Latino homebuyers by $7.3 billion and not changing the total amount of mortgage loan funding to White homebuyers. Ultimately, these banks predict that this proposal will widen the gap in mortgage loans between Latino and White homebuyers in the next year. These banks reported there will be no change in profits for many years, and they will need to reorganize their budgets to fund these mortgage loans. [*These banks reported there have been large and consistent increases in profits that will continue for many years, allowing them to fund mortgage loans for as many people as they want.*]

Pay gap (Men | Women)

Equality-enhancing, Limited [*Unlimited*] resource. This week, a joint report from the top tech firms in Silicon Valley revealed that female employees across the industry cumulatively earn $1.93 billion compared to their male employee counterparts who earn an estimated cumulative $25.6 billion. Leaders across these tech firms propose increasing the total pay to female employees by $500 million and not changing the total pay to male employees. Industry leaders expect that this proposal will reduce the gender pay gap over the next year. These firms reported there will be no change in profits for many years, and they will need to reorganize their budgets to pay employees. [*These firms reported there have been large and consistent increases in profits that will continue for many years, allowing them to pay employees any amount they want.]*

Inequality-enhancing, Limited [*Unlimited*] resource. This week, a joint report from the top tech firms in Silicon Valley revealed that female employees across the industry cumulatively earn $1.93 billion compared to their male employee counterparts who earn an estimated cumulative $25.6 billion. Leaders across these tech firms propose decreasing the total pay to female employees by $500 million and not changing the total pay to male employees. Industry leaders expect that this proposal will widen the gender pay gap over the next year. These firms reported there will be no change in profits for many years, and they will need to reorganize their

budgets to pay employees. [*These firms reported there have been large and consistent increases in profits that will continue for many years, allowing them to pay employees any amount they want.]*

Hiring gap (Non-disabled job seekers | Disabled job seekers)

Equality-enhancing, Limited [*Unlimited*] resource. According to the U.S. Department of Labor (DOL), there are roughly 1.4 million non-disabled U.S. federal government employees and only about 405,000 disabled U.S. employees. The DOL proposes to increase the number of disabled employees who are hired across these agencies by 260,000 and not changing the number of non-disabled employees. The DOL predicts that this proposal will reduce the hiring gap between disabled and non-disabled people within government agencies over the next year. The DOL reported there will be no change in available resources for many years, and they will need to reorganize their budget to meet their hiring goals. [*The DOL reported there have been large and consistent increases in available resources that will continue for many years, allowing them to hire as many people as they want.]*

Inequality-enhancing, Limited [*Unlimited*] resource. According to the U.S. Department of Labor (DOL), there are roughly 1.4 million non-disabled U.S. federal government employees and only about 405,000 disabled U.S. employees. The DOL proposes to decrease the number of disabled employees who are hired across these agencies by 260,000 and not changing the number of non-disabled employees. The DOL predicts that this proposal will increase the hiring gap between disabled and non-disabled people within government agencies over the next year. The DOL reported there will be no change in available resources for many years, and they will need to reorganize their budget to meet their hiring goals. [*The DOL reported there have been large and consistent increases in available resources that will continue for many years, allowing them to hire as many people as they want*.]

Unemployment gap (People with no criminal history | People with criminal history)

Equality-enhancing, Limited [*Unlimited*] resource. A recent Prison Policy Initiative (PPI) report revealed that, in the U.S., roughly 21.6 million ex-felon job seekers are unable to find a job compared to the 13.1 million job seekers without a criminal record who are unable to find a job. Various companies across the U.S. propose increasing the number of jobs available for ex-felon job seekers by 3.1 million and not changing the number of jobs available for non-felon job-seekers. The companies predict that this proposal will reduce the gap in unemployment between ex-felon and non-felon job seekers over the next year. These companies reported there will be no change in available resources for many years, and they will need to reorganize their budgets to meet their hiring goals. [*These companies reported there have been large and consistent increases in available resources that will continue for many years, allowing them to hire as many people as they want.*]

Inequality-enhancing, Limited [*Unlimited*] resource. A recent Prison Policy Initiative (PPI) report revealed that, in the U.S., roughly 21.6 million ex-felon job seekers are unable to find a job compared to the 13.1 million job seekers without a criminal record who are unable to find a job. Various companies across the U.S. propose reducing the number of jobs available for ex-felon job seekers by 3.1 million and not changing the number of jobs available for non-felon

job seekers. The companies predict that this proposal will increase the gap in unemployment between ex-felons and non-felon job seekers over the next year. These companies reported there will be no change in available resources for many years, and they will need to reorganize their budgets to meet their hiring goals. [*These companies reported there have been large and consistent increases in available resources that will continue for many years, allowing them to hire as many people as they want.*]

Startup funding gap (White entrepreneur | Black entrepreneur)

Equality-enhancing, Limited [*Unlimited*] resource. A recent report from the U.S. Small Businesses Association (SBA) detailed that Black entrepreneurs receive significantly less funding—an estimated $12.4 million—to start their businesses compared to White entrepreneurs, who received an estimated $241.8 million. Several venture capital firms propose increasing their total investments in startups founded by Black entrepreneurs by $8.2 million and not changing their total investments in startups founded by White entrepreneurs. Ultimately, these firms predict that this proposal will narrow the investment gap in Black and White businesses over the next year. These firms reported there will be no change in their available capital for many years, and they will need to reorganize their budgets to fund these startup investments. [*These firms reported there have been large and consistent increases in available capital that will continue for many years, allowing them to invest in as many startups as they want.]*

Inequality-enhancing, Limited [*Unlimited*] resource. A recent report from the U.S. Small Businesses Association (SBA) detailed that Black entrepreneurs receive significantly less funding—an estimated $12.4 million—to start their businesses compared to White entrepreneurs, who received an estimated $241.8 million. Several venture capital firms propose decreasing their total investments in startups founded by Black entrepreneurs by $8.2 million and not changing their total investments in startups founded by White entrepreneurs. Ultimately, these firms predict that this proposal will widen the investment gap in Black and White businesses over the next year. These firms reported there will be no change in their available capital for many years, and they will need to reorganize their budgets to fund these startup investments. [*These firms reported there have been large and consistent increases in available capital that will continue for many years, allowing them to invest in as many startups as they want.]*

College admissions gap (Continuing-generation students | First-generation students)

Equality-enhancing, Limited [*Unlimited*] resource. A recent report from eight accredited U.S. universities revealed that of the 510,000 college freshmen accepted to these universities, 173,000 were first-generation college students and about 337,000 were continuing-generation college students. Administrators from these eight universities propose increasing the number of first-generation students who are admitted by 86,000 and not changing the number of continuing-generation students who are admitted. Ultimately, these administrators predict that this proposal will decrease the gap in admissions between first-generation and continuing-generation college applicants. These universities reported there will be no change in available resources for many years, and they will need to reorganize their budgets to meet their student enrollment goals. *[These universities reported there have been large and consistent increases in*

available resources that will continue for many years, allowing them to enroll as many students as they want.]

Inequality-enhancing, Limited [*Unlimited*] resource. A recent report from eight accredited U.S. universities revealed that of the 510,000 college freshmen accepted to these universities, 173,000 were first-generation college students and about 337,000 were continuing-generation college students. Administrators from these eight universities propose decreasing the number of first-generation students who are admitted and not changing the number of continuing-generation students who are admitted. Ultimately, these administrators predict that this proposal will increase the gap in admissions between first-generation and continuing-generation college applicants in the next year. These universities reported there will be no change in available resources for many years, and they will need to reorganize their budgets to meet their student enrollment goals. *[These universities reported there have been large and consistent increases in available resources that will continue for many years, allowing them to enroll as many students as they want.]*

Study 5

Mortgage lending gap (White homebuyers | Latino homebuyers)

Limited [*Unlimited*] access. According to a recent report, in 2018 White homebuyers received roughly $386.4 billion in mortgage loans from banks while Latino buyers only received around $12.6 billion in mortgage loans overall.

Several banks propose increasing the total amount of mortgage loans to Latino homebuyers by $7.3 billion and not changing the total amount of mortgage loan funding to White homebuyers. Ultimately, these banks predict that this proposal will narrow the gap in mortgage loans between Latino and White homebuyers in the next year. These banks reported there will be no change in profits for many years, and they will need to reorganize their budgets to fund these mortgage loans. Therefore, these banks will provide a limited number of mortgage loans and the proposal will cause some White applicants to not receive funding. [*These banks reported there have been large and consistent increases in profits that will continue for many years, allowing them to fund mortgage loans for as many people as they want. Therefore, anyone who wants a mortgage loan can receive one.]*

Pay gap (Men | Women)

Limited [*Unlimited*] access. This week, a joint report from the top tech firms in Silicon Valley revealed that female employees across the industry cumulatively earn $1.93 billion compared to their male employee counterparts who earn an estimated cumulative $25.6 billion.

Leaders across these tech firms propose increasing the total pay to female employees by $500 million and not changing total pay to male employees. Industry leaders expect that this proposal will reduce the gender pay gap over the next year. These firms reported there will be no change in profits for many years, and they will need to reorganize their budgets to pay employees. Therefore, these firms will increase the salaries of a limited number of employees, and the proposal will cause some male employees to not receive salary increases. [*These firms reported there have been large and consistent increases in profits that will continue for many years, allowing them to pay employees any amount they want. Therefore, all employees can receive a salary raise.]*

Hiring gap (Non-disabled job seekers | Disabled job seekers)

Limited [*Unlimited*] access. According to the U.S. Department of Labor (DOL), there are roughly 1.4 million non-disabled U.S. federal government employees and only about 405,000 disabled U.S. employees.

The DOL proposes to increase the number of disabled employees who are hired across these agencies by 260,000 and not changing the number of non-disabled employees. The DOL predicts that this proposal will reduce the hiring gap between disabled and non-disabled people within government agencies over the next year. The DOL reported there will be no change in available resources for many years, and they will need to reorganize their budget to meet their hiring goals Therefore, these companies will have a limited number of job openings available and this proposal will cause some non-disabled applicants to not be hired. [*The DOL reported there have been large and consistent increases in available resources that will continue for many*

years, allowing them to hire as many people as they want. Therefore, all applicants can be hired.]

Unemployment gap (People with no criminal history | People with criminal history)

Limited [*Unlimited*] access. A recent Prison Policy Initiative (PPI) report revealed that, in the U.S., roughly 21.6 million ex-felon job seekers are unable to find a job compared to the 13.1 million job seekers without a criminal record who are unable to find a job.

Various companies across the U.S. propose increasing the number of jobs available for ex-felon job seekers by 3.1 million and not changing the number of jobs available for non-felon job-seekers. The companies predict that this proposal will reduce the gap in unemployment between ex-felon and non-felon job seekers over the next year. These companies reported there will be no change in available resources for many years, and they will need to reorganize their budgets to meet their hiring goals. Therefore, these companies will have a limited number of job openings available and this proposal will cause some non-felon applicants to not be hired. [*These companies reported there have been large and consistent increases in available resources that will continue for many years, allowing them to hire as many people as they want. Therefore, all applicants can be hired.]*

Startup funding gap (White entrepreneur | Black entrepreneur)

Limited [*Unlimited*] access. A recent report from the U.S. Small Businesses Association (SBA) detailed that Black entrepreneurs receive significantly less funding—an estimated $12.4 million—to start their businesses compared to White entrepreneurs, who received an estimated $241.8 million.

Several venture capital firms propose increasing their total investments in startups founded by Black entrepreneurs by $8.2 million and not changing their total investments in startups founded by White entrepreneurs. Ultimately, these firms predict that this proposal will narrow the investment gap in Black and White businesses over the next year. These firms reported there will be no change in their available capital for many years, and they will need to reorganize their budgets to fund these startup investments. Therefore, these firms will invest in a limited number of companies and this proposal will cause some White entrepreneurs to not receive investments. [*These firms reported there have been large and consistent increases in available capital that will continue for many years. Therefore, all entrepreneurs in need of investment can receive it.]*

College admissions gap (Continuing-generation students | First-generation students)

Limited [*Unlimited*] access. A recent report from eight accredited U.S. universities revealed that of the 510,000 college freshmen accepted to these universities, 173,000 were first-generation college students and about 337,000 were continuing-generation college students.

Administrators from these eight universities propose increasing the number of first-generation students who are admitted by 86,000 and not changing the number of continuing-generation students who are admitted. Ultimately, these administrators predict that this proposal will decrease the gap in admissions between first-generation and continuing-generation college applicants. These universities reported there will be no change in available resources for many

years, and they will need to reorganize their budgets to meet their student enrollment goals. Therefore, these universities will admit a limited number of students and the proposal will cause some continuing-generation applicants to not be admitted. [*These universities reported there have been large and consistent increases in available resources that will continue for many years, allowing them to admit as many students as they want. Therefore, all applicants can be admitted.]*

Study 7

Win-win equality-enhancing. After teams complete the tasks each week, we select participants to receive a bonus for their efforts. In previous weeks, RATTLERS have been selected to receive bonuses more often than EAGLES. We are implementing a new procedure to allocate bonuses more equally across the groups. With this change, we will provide 50 additional bonuses to EAGLES and only 5 more bonuses to RATTLERS.

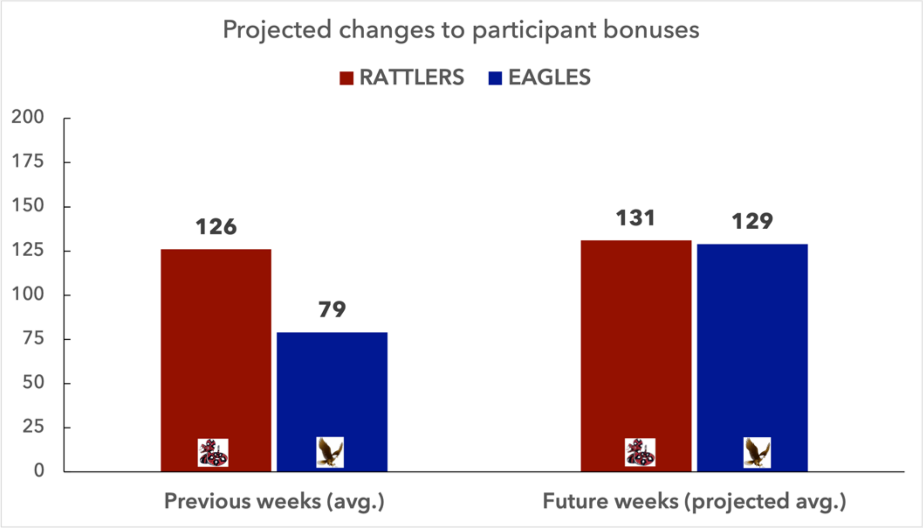

Lose-lose inequality-enhancing. After teams complete the tasks each week, we select participants to receive a bonus for their efforts. In previous weeks, RATTLERS have been selected to receive bonuses more often than EAGLES. We are implementing a new procedure to allocate more bonuses to whichever group previously received more bonuses. With this change, we will provide 50 fewer bonuses to EAGLES and only 5 fewer bonuses to RATTLERS.

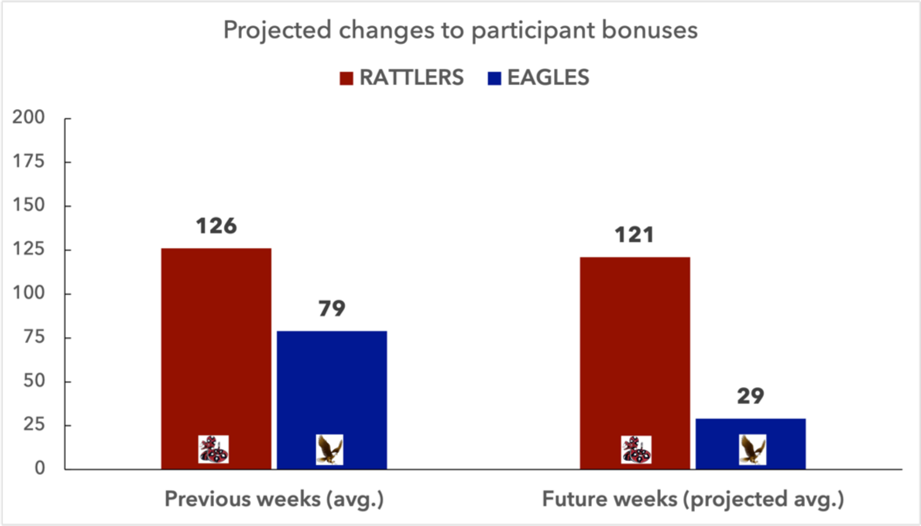

Study 8

Joint evaluation. After teams complete the tasks each week, we randomly select participants to receive a bonus for their efforts. In previous weeks, RATTLERS have been selected to receive bonuses more often than EAGLES. We are considering implementing a new procedure to allocate bonuses more equally across the groups. We are considering two options:

> Option A is to provide 50 additional bonuses to EAGLES and not change the number of bonuses to RATTLERS.
>
> Option B is to provide 50 fewer bonuses to RATTLERS and not change the number of bonuses to EAGLES.

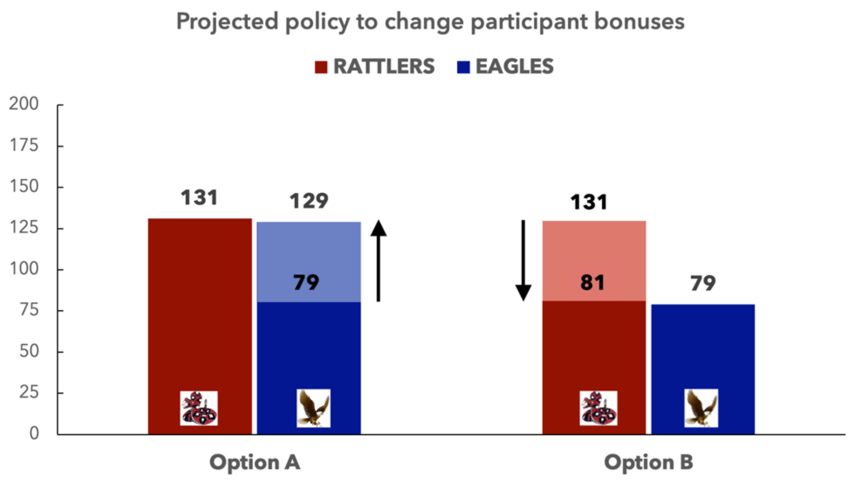

Separate evaluation. After teams complete the tasks each week, we randomly select participants to receive a bonus for their efforts. In previous weeks, RATTLERS have been selected to receive bonuses more often than EAGLES. We are considering implementing a new procedure to allocate bonuses more equally across the groups. With this change, we will provide 50 additional bonuses to EAGLES and not change the number of bonuses to RATTLERS.

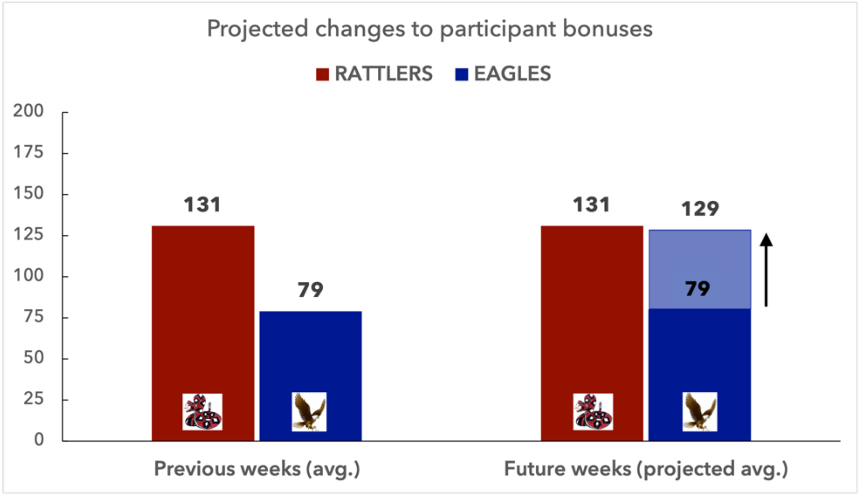

Supplement: Supplementary file 1 — Tables S1 to S34 Figs. S1 to S6 Policy conditions for Studies 1 to 5, 7, and 8 [file sciadv.abm2385_sm.pdf]
